# Stabilizing Solid-State Battery Interfaces with a Polyelectrolyte Complex Nanocoating

**DOI:** 10.1021/acsnano.6c05725

**Published:** 2026-08-03

**Authors:** Bing-Xuan Shi, Daniil R. Nosov, Timo Weintraut, Mariapaola Staropoli, Thomas Demuth, Felix Schnaubelt, Sebastian Leonard Benz, Kilian Vettori, Xiuxia Zuo, Jingui Yang, Florian Strauss, Kerstin Volz, Anja Henss, Alexander S. Shaplov, Felix H. Richter

**Affiliations:** † Institute of Physical Chemistry & Center for Materials Research (LaMa), 54250 Justus-Liebig-University Giessen, Heinrich-Buff-Ring 17, Giessen 35392, Germany; ‡ 87145 Luxembourg Institute of Science and Technology, Functional Polymeric and Particulate Materials Unit, 5 Avenue Des Hauts-Fourneaux, Esch-sur-Alzette 4362, Luxembourg; § Institute of Experimental Physics 1 & Center for Materials Research (LaMa), 9175 Justus-Liebig-University Giessen, Heinrich-Buff-Ring 17, Giessen 35392, Germany; ∥ Philipps-Universität Marburg, mar.quest | Marburg Center for Quantum Materials and Sustainable Technologies, Marburg 35032, Germany; ⊥ School of New Energy, 74748 Ningbo University of Technology, No. 201 Fenghua Road, Jiangbei District, Ningbo, Zhejiang 315211, P. R. China; # Institute of Nanotechnology (INT), 28335 Karlsruhe Institute of Technology (KIT), Kaiserstr. 12, Karlsruhe 76131, Germany; □ Philipps-Universität Marburg, Department of Physics, Marburg 35032, Germany

**Keywords:** solid-state batteries, solid electrolyte, polymer coating, polyelectrolyte complex, interface degradation

## Abstract

Sulfide-based solid electrolytes (SEs) are promising enablers of next-generation solid-state batteries (SSBs), yet their practical implementation is limited by interfacial instability with anode and cathode active materials. Current interfacial mitigation strategies rely primarily on inorganic coatings, which are often unable to accommodate chemo-mechanical strain. Here, we introduce a new dynamic ion gel nanocoating that stabilizes both cathode and anode interfaces in sulfide-based SSBs. It is a type of ion-conductive polyelectrolyte complex (PEC), which is formed via complex coacervation between oppositely charged polyelectrolytes. Controlled mixing of a polycation bearing ammonium groups with TFSI^–^ counteranions and a polyanion featuring pendant TFSI^–^ groups with Li^+^ countercations yields a homogeneous, solution-processable dynamic ion gel accompanied by LiTFSI release. This enables uniform, thin (≈1–3 nm), and scalable PEC nanocoatings on active material particles via spray drying. Dynamic ionic cross-linking endows the PEC with enhanced viscoelasticity and improved ionic conductivity relative to the parent polycation, allowing it to adapt to solid–solid interfaces while maintaining efficient ion transport. SSBs employing PEC-coated silicon anodes and PEC-coated LiNiO_2_ cathodes exhibit improved cycling stability through suppression of interfacial side reactions. Thus, the PEC nanocoating acts as a scalable stabilizer of solid–solid interfaces in SSBs.

## Graphical abstract

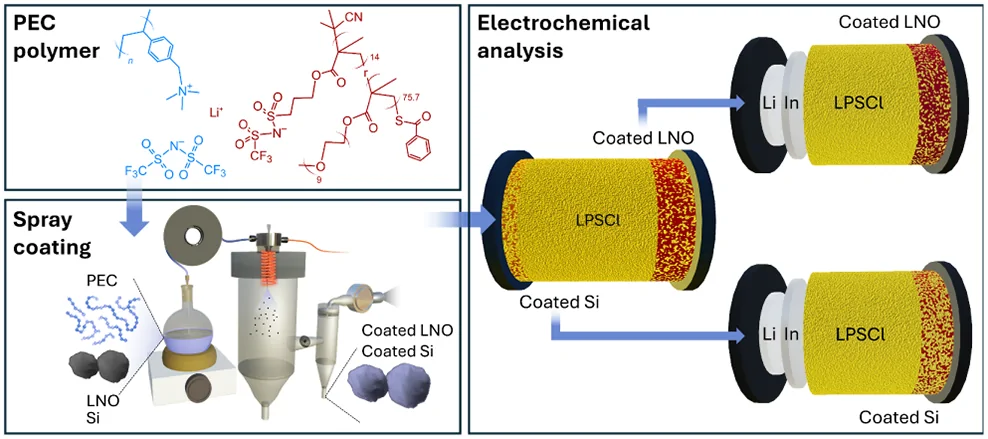

## 1 Introduction

Solid electrolyte batteries (SEBs) are a specific subtype of solid-state batteries that use inorganic solid electrolytes (SEs) as a catholyte and separator.^1^ They potentially offer significant benefits over liquid electrolyte batteries (LEBs), including leak prevention, enhanced safety, cost savings, and greater energy density.^2–7^ Sulfide-based SEs, such as Li_6_PS_5_Cl (LPSCl), have sufficient ionic conductivity of ≈2 mS·cm^–1^ for realizing model SEBs;^8,9^ however, severe interfacial degradation between electrode active materials (AMs) and the sulfide-based SEs^10,11^ compromises their electrochemical performance.^2,12^

Nickel-rich cathode active materials (CAMs) are widely used as CAMs.^13,14^ The trend toward higher Ni content in LiNi_*x*_Mn_*y*_Co_*1‑x‑y*_O_2_ (NCM), such as LiNi_0.9_Mn_0.05_Co_0.05_O_2_ (NCM90), aims to increase energy density.^14^ LiNiO_2_ (LNO), with the highest Ni content, is preferred to maximize the practical capacity and reduce cost.^15^ However, LNO undergoes more severe cathode-electrolyte interphase (CEI) degradation with LPSCl than NCMs.^16^ Therefore, LNO serves as a challenging model for studying interfacial degradation in nickel-rich CAMs.

The oxidation of LPSCl above 2.2 V vs Li^+^/Li results in the formation of polysulfide species, such as −[S]_*n*_–.^17,18^ These side products are insulating and reduce first-cycle Coulomb efficiency.^17,19–21^ Additionally, at a 0% state of charge (SOC), chemical reactions already take place at the CEI, causing a decrease in first-cycle capacity.^22^ When the SOC increases to ≈4.2 V vs Li^+^/Li, oxygenated reactions cause the formation of a passivation layer, hindering charge transfer.^19,23,24^ Moreover, nickel-rich CAMs undergo chemo-mechanical fracture due to oxygen loss and volumetric changes, especially above 4.2 V vs Li^+^/Li.^25,26^ To study CEI degradation, the present study focuses on single-crystal LNO, which minimizes particle fracture^15,25^ but exhibits pronounced CEI passivation during cycling,^13,15^ while corroborating the results with NCM90.

When brought in contact with anode materials, LPSCl reduces below 1.7 V vs Li^+^/Li, forming a solid electrolyte interphase (SEI).^18^ Silicon is an attractive anode material due to its low cost, air stability, and exceptionally high theoretical capacity of 3579 mAh·g^–1^, approaching the theoretical capacity of lithium metal (3860 mAh·g^–1^).^27,28^ In addition, silicon undergoes the alloying process at ≈0.3 V vs Li^+^/Li and provides higher energy density compared to other alloy-type anode materials.^29^ Despite all these advantages, silicon undergoes substantial volume expansion during lithiation/delithiation (≈300%).^30^ This repeated expansion and contraction mechanically degrade the SEI layer and promote further reduction of LPSCl, ultimately accelerating interfacial instability and deteriorating electrochemical performance.^28,31–34^

Surface coatings on nickel-rich CAMs^35,36^ and silicon anodes^37,38^ are applied to improve the interfacial stability at the CEI and SEI, respectively. Coating materials should be electronically insulating, ionically conducting, and electrochemically stable.^39^ To keep lithium-ion transport resistance below 1 Ω·cm^2^ with a 1 nm thick coating, an ionic conductivity of above 0.1 μS·cm^–1^ is necessary.^40^ Consequently, optimization of the coating thickness and coverage is critical to avoid impeding lithium-ion transport. However, inorganic coatings with high Young’s modulus (such as LiNbO_3_ ≈ 195 GPa) can crack or detach during volume change.^40,41^ In contrast, the inherent flexibility of polymers (such as poly­(ethylene oxide) ≈ 700 MPa)^42^ minimizes coating fractures.

Our previous work demonstrated that polycations (PCs), such as poly­((4-vinylbenzyl)­trimethylammonium bis­(trifluoromethanesulfonylimide)) (PVBTA-TFSI), can effectively induce surface coatings on nickel-rich CAMs through strong electrostatic interaction.^43^ However, because these PC-based coatings do not supply lithium ions, they may lead to a reduction in capacity, even though they stabilize the cathode interface.^44^ In contrast, polyanions (PAs), such as sulfonated polyphenylene sulfone in its lithiated form (sPPSLi), can provide lithium ions but exhibit insufficient ionic conductivity and modest electrostatic attraction to form robust coatings.^44^ These complementary limitations motivate the exploration of polyelectrolyte complexes (PECs) that integrate a PC to drive coating formation with a PA that simultaneously contributes lithium ions, thereby uniting the advantages of both ionic polymers.

However, before applying PECs as coatings, several challenges must be addressed:

1 Poor solubility of the PECs. Typically, when solutions of oppositely charged polyelectrolytes are mixed in low-boiling-point solvents at stoichiometric ionic ratios, the ion-exchange reactions trigger coacervation and PEC precipitation, which usually renders PECs insoluble and unsuitable for wet-coating processes.^45,46^ It is also worth noting that the majority of known PECs are almost exclusively based on water-soluble polyelectrolytes. Switching to high-boiling-point polar aprotic solvents in rare examples^47^ can improve solubility, but it introduces practical limitations, such as slow solvent evaporation and more difficult drying of the resultant coatings. Alternatively, adding salts to aqueous polyelectrolyte solutions can screen polymeric charges and yield colloidal dispersions.^48^ However, a true molecular solution is required for high-quality coatings, as colloidal systems containing micrometer-sized aggregates cannot form uniform coatings.^48,49^

2 High glass-transition temperature (*T*_g_) of polyelectrolytes participating in PEC formation. A widely studied polyelectrolyte pair for PEC formation is poly­(sodium 4-styrenesulfonate) (NaPSS) combined with poly­(diallyldimethylammonium chloride) (PDADMAC).^50^ However, both polymers exhibit high *T*_g_: approximately 228 °C for NaPSS^51^ and 195–310 °C for PDADMAC, depending on the associated counterion.^52^ These elevated *T*_g_ values result in PEC films that are intrinsically rigid, mechanically stiff in the dry state, and low in conductivity. Furthermore, the strong electrostatic interactions between the oppositely charged chains^53^ render the dry coatings brittle,^46^ prone to fracture, and loss of functionality.^40^
3 Low ionic conductivity of the PECs. Most polyelectrolytes used to prepare self-standing PEC films exhibit high *T*_g_ values and inherently low ionic conductivities under dry conditions. In the absence of water, these rigid ionic matrices significantly hinder lithium-ion mobility, limiting their suitability for battery applications. Therefore, there is a clear need to design and synthesize new polyelectrolytes capable of forming solvent-soluble PECs that are compatible with wet-coating processes and can yield nanometer-sized, flexible films with high ionic conductivity.

Previous studies have not yet established a strategy for producing processable nanometer-scale polyelectrolyte coatings that rely solely on dense ionic interactions while maintaining mechanical flexibility under dry conditions.^46^ Recently, our group developed an alternative strategy for synthesizing PECs based on the complex coacervation of oppositely charged poly­(ionic liquids), notably without removing the low-molecular-weight salts generated during ion exchange.^54^ Mixing acetone solutions of these two poly­(ionic liquids) with ion pairs in a stoichiometric ratio initiates coacervation through ion exchange. As the solvent evaporates, a new subclass of PECs is formed, namely dynamic ion gels, which offer ionic conductivity, mechanical flexibility, and structural stability.

Building on this methodology, in the present study, we combine two suitable polyelectrolytes at a 60:40 wt % ratio to form a homogeneous, solvent-soluble dynamic ion gel, while the ion-exchange process releases free lithium bis­(trifluoromethanesulfonyl)­imide (LiTFSI) that remains entrapped within the material. This stable dynamic ion gel is applied to fabricate uniform 1∼3 nm-thick PEC coatings on silicon anode particles and nickel-rich CAM particles (LNO, NCM90), which demonstrate improved electrochemical performance in LPSCl-based ^Si^SEB^LNO^. Time-of-flight secondary ion mass spectrometry (TOF-SIMS) and X-ray photoelectron spectroscopy (XPS) confirm significantly reduced degradation in both the CEI and SEI when the novel PEC coatings are employed. This paper first presents the preparation and characterization of the materials in Sections 2.1∼[Sec sec2.3], followed by the full-cell demonstration using LNO and a Si anode in Section 2.4. To further distinguish the contributions from the cathode and anode, we then separately analyze the LNO half-cell in Sections 2.5∼[Sec sec2.7] and the Si half-cell in Sections 2.8∼[Sec sec2.10].

## 2 Results and Discussion

### 2.1 Synthesis of PEC and its Characterization

To meet the targeted PEC performance requirements, the study begins with the molecular design of polyelectrolytes serving as the PA and PC components (Figure 1). PVBTA-TFSI was selected as the PC component for several reasons: (i) its moderate glass-transition temperature, *T*_g_ (84 °C, Figure S1 and Table S1), (ii) its ability to form self-supporting films with good mechanical properties, (iii) its sufficient solubility in common organic solvents, and (iv) its proven capability to generate surface coatings on nickel-rich CAMs through strong electrostatic interaction.^43^ The synthesis of the PC was carried out in two steps, following the procedure previously reported by our group:^43^ first, VBTA-Cl was polymerized via free-radical polymerization of VBTA-Cl in an aqueous medium using Na_2_S_2_O_8_ as the initiator. This was followed by ion exchange with LiTFSI to afford the target polymer. The isolated PC exhibited an *M*_n_ of 29500 g·mol^–1^ and a moderate dispersity (*M*_w_/*M*_n_ = 2.96) (Figure S4 and Table S1).

**Figure 1:**
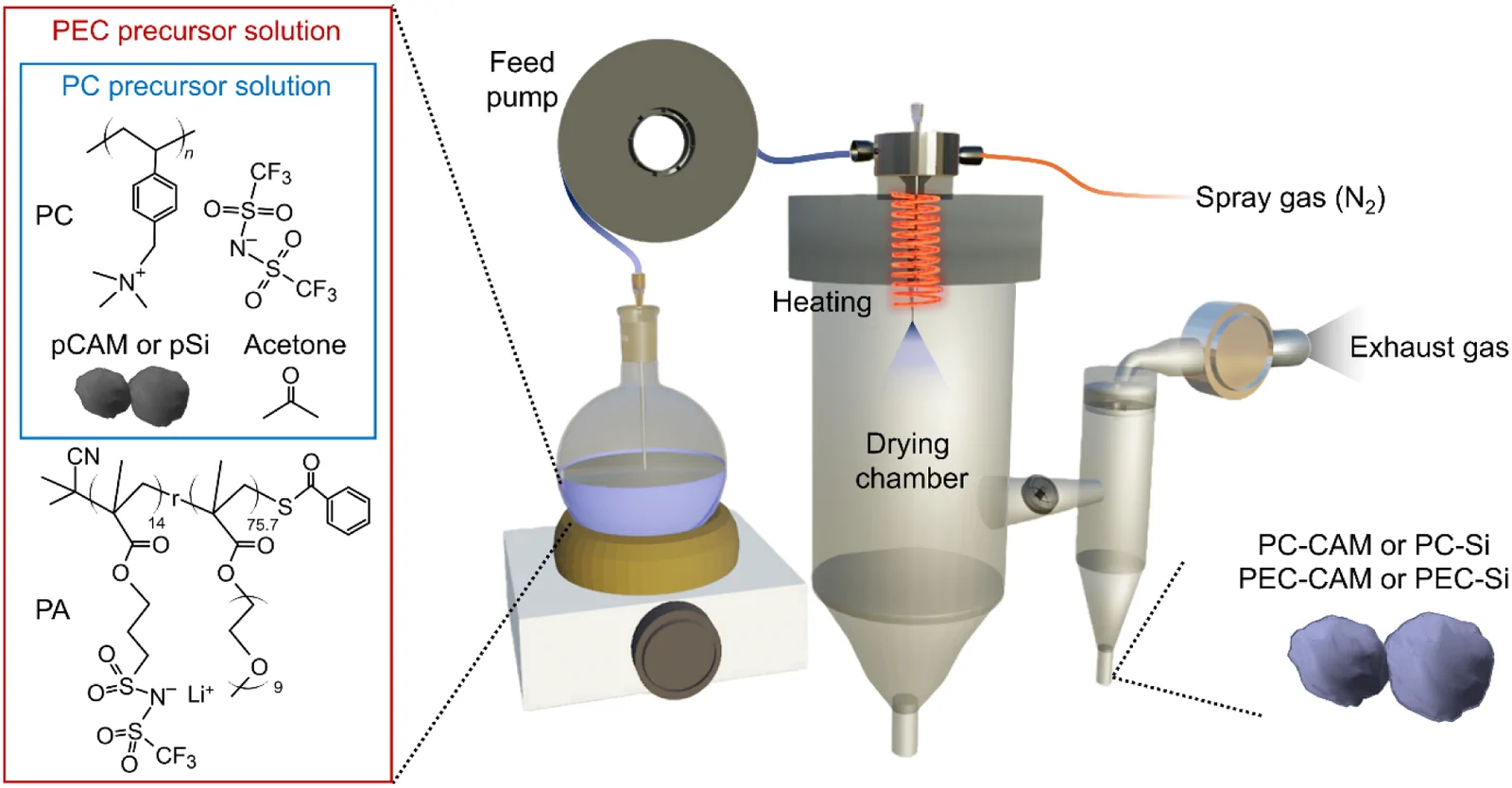
Schematic illustration of the spray drying process used to coat AM particles, including LNO, NCM90, and silicon with PEC or PC polymers (left). A precursor dispersion of polymers and AMs in acetone is atomized into fine droplets, which dry rapidly in the chamber to form coated particles. The resulting coated AM particles are collected with a productivity of approximately 70 wt %.

Poly­[(lithium1-[3-(methacryloyloxy)­propylsulfonyl]-1-(trifluoromethanesulfonyl)­imide)-r-(poly­(ethylene glycol) methyl ether methacrylate)] was chosen as the PA counterpart owing to several favorable characteristics: (i) a relatively high ionic conductivity (8.2 × 10^–7^ S·cm^–1^ at 25 °C; Table S1); (ii) a single-ion-conducting architecture in which Li^+^ ions are the dominant mobile species; (iii) excellent solubility in common organic solvents;^55^ and (iv) efficient ion dissociation resulting from the highly delocalized charge distribution of the tethered TFSI groups, together with the plasticizing effect of the oxyethylene segments, which reduce the polymer *T*_g_ (−54 °C, Table S1) and facilitate lithium-ion transport.The synthesis of PA was performed via controlled random reversible addition–fragmentation chain-transfer (RAFT) copolymerization of lithium 1-[3-(methacryloyloxy)­propylsulfonyl]-1-(trifluoromethanesulfonyl)­imide and PEGM monomers, using azobis­(isobutyronitrile) (AIBN) as the initiator and 2-cyano-2-propyl benzodithioate (CPDB) as the chain-transfer agent, following the procedure previously reported by our group.^55^ The resulting PA exhibited an *M*_n_ of 41000 g·mol^–1^ and a narrow dispersity (*M*_w_/*M*_n_ = 1.50) (Figure S4 and Table S1).

For spray drying onto AM particles, achieving a transparent true polymer solution (solute particle size < 1 nm) in a low-boiling solvent is essential to ensure uniform coating formation.^43,44^ Consequently, the PEC must remain fully soluble during the mixing of the PA and PC, without undergoing coacervation or phase separation. This requires sufficiently weak electrostatic interactions between the oppositely charged polyelectrolytes. The use of highly delocalized TFSI^–^ anions in both the PC and PA successfully reduces these interactions, as evidenced by only minimal shifts in the TFSI^–^ stretching bands in the FTIR spectrum of the PEC compared to the spectra of the individual components (Figure S5). Despite the excellent solubility of both polyelectrolytes in acetone and their expected weak interchain ionic attraction, mixing them at a stoichiometric ratio of ionic pairs still leads to PEC coacervation and partial precipitation. To overcome this, a series of PC:PA ratios was systematically investigated. A clear, stable solution is obtained at a 60:40 wt % ratio, avoiding phase separation. This homogeneous solution was subsequently evaporated, and the properties of the resulting PEC were evaluated and compared with those of the individual PC and PA components.

The temperature dependence of ionic conductivity for the PEC is further examined by electrochemical impedance spectroscopy (EIS) (Figure S6). The conductivity increases monotonically with temperature; however, the relationship is nonlinear and deviates from the classical Arrhenius relationship. Instead, the data follow a Vogel–Fulcher–Tammann (VFT) trend (Table S2) as is characteristic of polymer electrolytes, where ionic transport arises from a combination of free ions hopping, coupled with segmental motions of the polyelectrolyte backbone.

The thermal properties of the polyelectrolytes are investigated by differential scanning calorimetry (DSC) (Figures S1∼S3) and thermogravimetric analysis (TGA) (Figure S7). Both PC and PA exhibit a single DSC transition at −54 and 84 °C, respectively, corresponding to their *T*_g_. In contrast, the DSC trace of the PEC (Figure S3) displays two distinct thermal events: a *T*_g_ at −14 °C, representing an intermediate value between those of the parent polymers, and an endothermic peak at 183 °C, assigned to the melting of in situ-generated LiTFSI. The thermal stability of the PEC is further assessed by TGA, revealing an onset of mass loss temperature (*T*_onset_) at 230 °C (Figure S7). This decomposition onset lies between those of PA and PC (Figure S7 and Table S1) and is sufficiently high to support the applicability of the PEC in battery systems.

The viscoelasticity of PC, PA, and PEC in the linear viscoelastic regime is analyzed by small-amplitude oscillatory shear rheology (Figure S8 and Table S1). Measurements are performed at 25 °C using a parallel-plate geometry with a constant strain amplitude of 1%. The storage modulus (G′) and loss modulus (G″) are recorded as functions of angular frequency. PA exhibits a rheological profile characterized by a pronounced separation between G′ and G″ and the absence of a terminal flow region (i.e., no G′ ∼ ω^2^ and G″ ∼ ω scaling). The strong increase in G′ with frequency indicates enhanced segmental mobility, while the condition G″ > G′ across the entire frequency range confirms its predominantly viscous, liquid-like flow. In contrast, PC, being in the glassy state at 25 °C, displays G′ > G″ throughout the full frequency window, indicative of a rigid, solid-like response. Formation of the PEC results in a substantial increase in both G′ and G″ compared to PA. Importantly, the ionic interactions between the oppositely charged polyelectrolytes further enhance G′ relative to PC. The PEC shows G′ > G″ at low to intermediate frequencies (ω < 60 rad s^–1^), transitioning to G′ ≤ G″ at higher frequencies. Such a relationship is characteristic of a soft, viscoelastic solid, making the PEC a promising candidate for forming mechanically robust yet flexible coatings on active material particles to improve the stability of solid-state battery interfaces.

Following the preparation of the soluble PEC and the evaluation of its properties (Table S1), several key advantages of its formation and use can be identified:

i While the PC component alone is capable of forming mechanically robust films and coatings, its ionic conductivity is too low for practical electrochemical applications. Moreover, the conductivity is primarily mediated by mobile TFSI^–^ anions, which may partially hinder lithium-ion transport.
ii Incorporation of LiTFSI into the PC matrix leads to only a modest increase in ionic conductivity and remains insufficient to achieve the conductivity levels required for applications in batteries. Moreover, even the addition of a relatively small amount of LiTFSI to the PC matrix, corresponding to the quantity generated *in situ* during PEC formation (PC:LiTFSI = 15:1 by weight, equivalent to a molar ratio of 9.4:1), results in pronounced phase separation (Figure S9). This phase instability compromises the homogeneity, film quality, and long-term reliability of the material, rendering such salt-filled PC systems unsuitable for use in high-performance electrochemical devices.
iii In contrast, the PA component alone provides the desired level of ionic conductivity, with charge transport occurring exclusively through the migration of Li^+^ ions. However, because of its low *T*_g_, the polymer exhibits pronounced cold flow and lacks the mechanical stability required for durable coatings.
iv Formation of the PEC results in an ionic conductivity of 8.8 × 10^–7^ S·cm^–1^ at 25 °C, exceeding that of both the individual PC and PA components (2.1 × 10^–9^ and 8.2 × 10^–7^ S·cm^–1^ at 25 °C, respectively). This enhancement can be attributed to several synergistic effects, including a substantial reduction in *T*_g_ relative to the PC, improved ion dissociation promoted by the oxyethylene segments, and the *in situ* formation of LiTFSI. The latter facilitates ion transport by partially screening the electrostatic interactions between oppositely charged polymer chains.^56,57^

v Finally, the weak ionic interactions established between the PA and PC chains give rise to flexible, yet mechanically robust coatings. This unique combination of high ionic conductivity and favorable mechanical properties cannot be achieved with a PA component alone. In summary, the PEC is particularly attractive because it combines the single-ion-conducting nature of the PA component with the excellent film-forming and coating capabilities of the PC component while simultaneously mitigating the key limitations associated with each individual polymer.

### 2.2 Preparation and Characterization of PEC-Coated Active Materials

After optimizing the PEC composition and obtaining an acetone-soluble PEC, solutions of PC and PEC in acetone are prepared at a concentration of 0.67 mg·mL^–1^. PA alone is excluded as its cold-flowing nature and unsuitable electrostatic interaction with the particle surface forms a non-uniform NCM coating.^44^ The AM particles are then added to these polyelectrolyte solutions such that the polyelectrolyte content in the resulting dispersions does not exceed 1 wt % relative to the mass of the AMs. This concentration is selected because previous studies demonstrated that such a loading produces an approximately 2 nm coating thickness.^43,44^ Finally, using a spray drying method, several AMs coated with PC or PEC are obtained: PC-coated LNO (PC-LNO), PEC-coated LNO (PEC-LNO), PEC-coated NCM90 (PEC-NCM90), PC-coated silicon (PC-Si), and PEC-coated silicon (PEC-Si). The pristine AMs are designated as pLNO, pNCM90, and pSi.

(Scanning) transmission electron microscopy ((S)­TEM) coupled with energy-dispersive X-ray spectroscopy (EDX) is used to evaluate the coverage and thickness of PC and PEC coatings on LNO and silicon. Figure 2 presents high-resolution TEM images, revealing that pLNO and pSi exhibit exposed crystalline lattice planes, whereas PC- and PEC-coated samples display a uniform 1∼3 nm thin amorphous layer covering the crystalline layers. These findings are consistent with our prior study on PC-coated NCM.^43^ It is worth noting that the ionic conductivity of the PEC polymer is ≈8.8 × 10^–7^ S·cm^–1^ at 25 °C (Figure S6 and Table S1), suggesting a 1∼3 nm coating may add less than 1 Ω·cm^2^ lithium-ion transport resistance to the SEB.^40^

**Figure 2:**
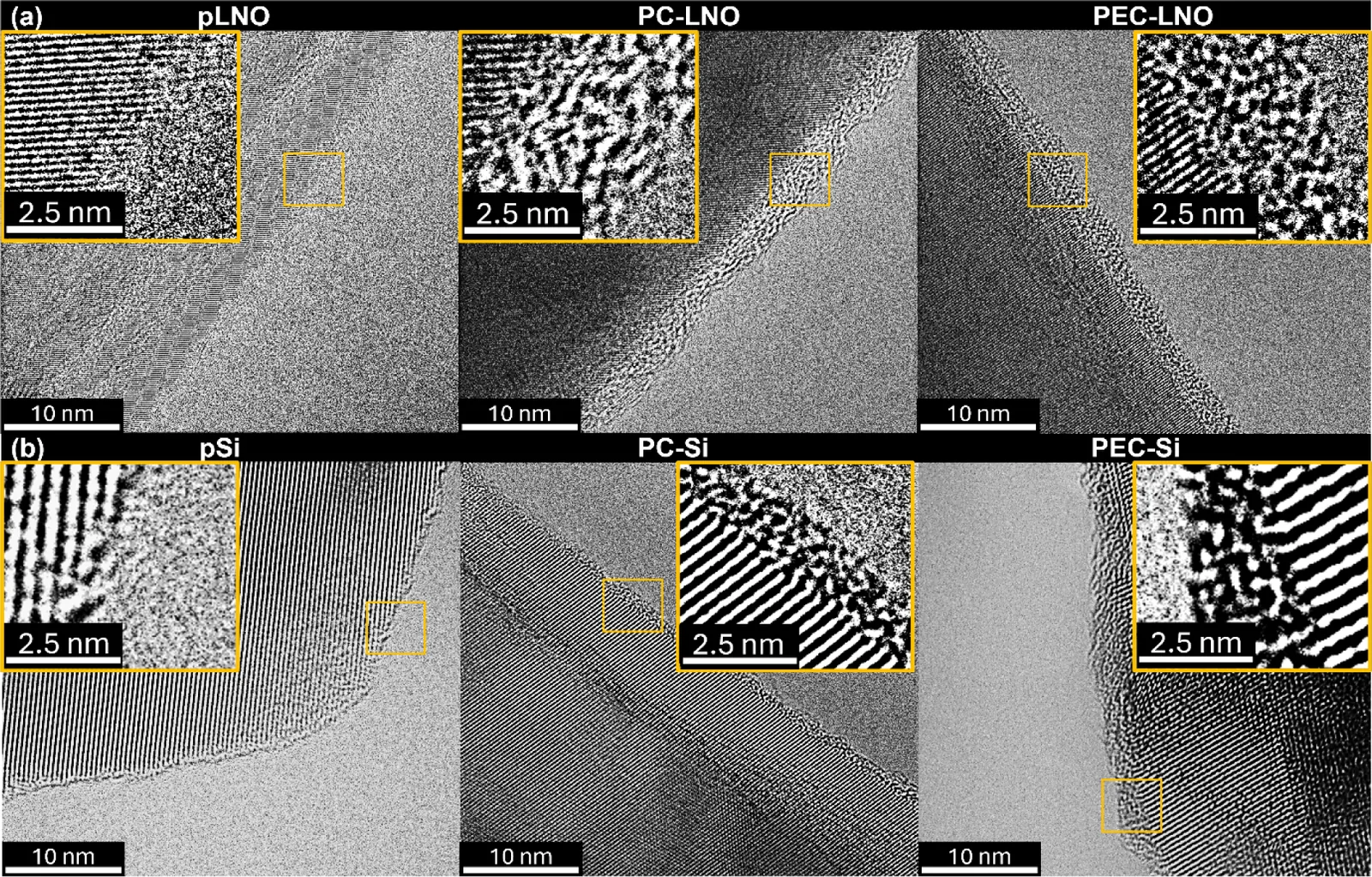
High-resolution TEM images of (a) pLNO, PC-LNO, and PEC-LNO, and (b) pSi, PC-Si, and PEC-Si. The insets provide magnified views of the coating layer on the crystalline lattice planes, clearly resolving a uniform coating layer with an estimated thickness of approximately 1∼3 nm.

STEM-EDX analysis, shown in Figures S10∼S13, confirms a uniform coating in the case of PEC-LNO, PC-LNO, PEC-Si, and PC-Si surfaces. Coated AM particles exhibit significantly stronger carbon signals arising from the polymer coating compared to the uncoated AM particles, with the highest carbon intensity consistently observed at the AM particle edges. In addition, oxygen can be used as a complementary indicator to verify the presence of the coating on PEC-Si and PC-Si surfaces. In contrast, oxygen cannot serve as a reliable indicator for LNO-based samples, as LNO inherently contains a high oxygen content that substantially exceeds the amount contributed by the coating.

Scanning electron microscopy (SEM) using an energy-selective backscattered electron detector (ESB) (Figure S14) shows no evidence of polymer aggregation on PEC-LNO particles, supporting the TEM observations of a uniform coating and indicating uniform coating formation over a larger length scale. Detecting such aggregates on silicon-based samples, however, is more challenging due to the similar densities of silicon and carbon, which reduce ESB contrast. Overall, taken together, the SEM, TEM, and EDX analyses confirm that both PEC and PC form uniform coatings on LNO and silicon particles, with a thickness of approximately 1∼3 nm, sufficient to prevent direct physical contact between the AM and LPSCl particles.

### 2.3 Electrochemical Stability of the Polymer Coating

Cyclic voltammetry (CV) is employed to evaluate the electrochemical stability window (ESW) of the PC and PEC in contact with inactive materials, such as vapor-grown carbon fibers (VGCFs) and stainless steel stamps (SS), using pellet-type cells with a scan range from 0 to 4.5 V vs In/LiIn. PC-coated and PEC-coated VGCFs, prepared via spray drying, serve as working electrodes to enhance the interfacial contact area with LPSCl, while pristine VGCF is used as the reference.^17^ A LiIn alloy is selected as the counter electrode because of its well-established kinetic stability with LPSCl.^17,58^ The resulting cell configuration was SS|LiIn|LPSCl|VGCF|SS.

Figure S15 shows that both PEC- and PC-coated VGCFs exhibit a diminished current density and have no additional side reaction peaks compared to pristine VGCF, confirming the stability of PEC and PC against LPSCl. Moreover, the lower current density observed with PEC-coated VGCF compared to that with PC-coated VGCF suggests that the rubber-like mechanical properties of PEC confer superior protection. This elasticity helps maintain continuous coverage on the VGCF, preventing the coating from being detached during cell preparation.

However, because VGCF does not fully replicate the surface chemistry or operating potentials of LNO or Si, the CV results should be considered an indirect stability assessment. Therefore, they cannot conclusively determine whether the coating on CAMs remains stable or partially decomposes into a beneficial passivation layer during cell cycling. Nevertheless, the galvanostatic charge–discharge profiles shown in Figures S15, S16, and S24 show no obvious additional side reactions attributable to the polymers. Overall, both PEC- and PC-coated VGCF have a stabilized interface with LPSCl and record fewer side reactions at the VGCF|LPSCl interface.

### 2.4 Impact of Coatings on the Cycling Stability of ^Si^SEB^LNO^ Full Cell

Cycling stability of ^Si^SEB^LNO^ (an area-specific capacity of 3.3 mAh·cm^–2^ based on 200 mAh·g^–1^ of LNO, N/P ratio: 1.3) with an SS|VGCF|Si|LPSCl|LNO|VGCF|SS configuration (Figure 3a) is conducted under 60 MPa at different C-rates (0.1C, 0.25C, 0.5C, 1C), followed by 0.1C for continuous cycling. ^Si^SEB^LNO^ using pristine and PEC-coated AMs is denoted as ^pSi^SEB^pLNO^ and ^PEC‑Si^SEB^PEC‑LNO^, respectively. The specific discharge capacity (*q*_dis_) and C-rates are calculated based on the amount of LNO.

**Figure 3:**
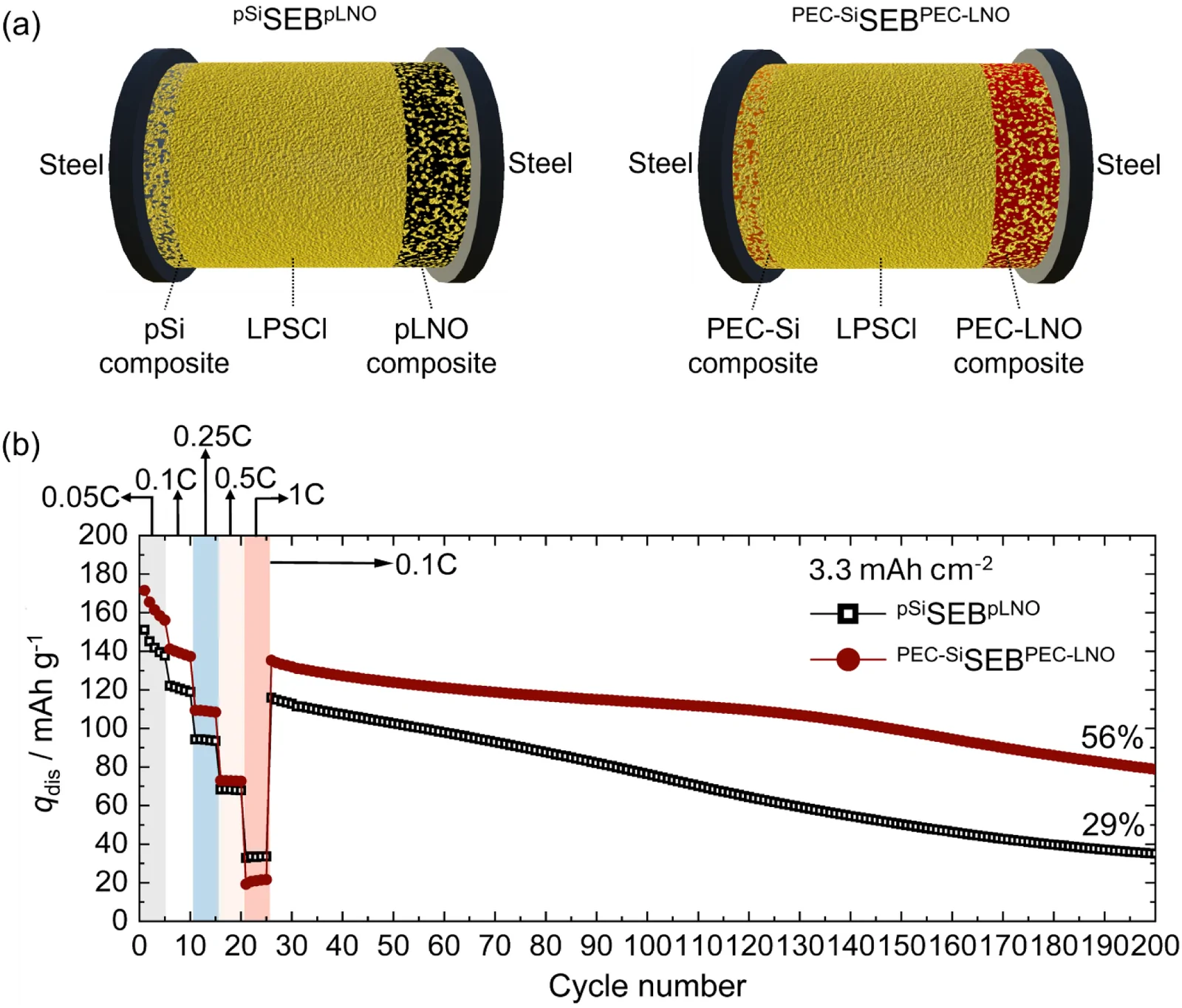
(a) Schematic representations of the ^pSi^SEB^pLNO^ and ^PEC‑Si^SEB^PEC‑LNO^ full-cell configurations (area-specific capacity: 3.3 mAh·cm^–2^, LNO: 13.2 mg, Si: 1 mg, N/P ratio: 1.3). (b) Discharge capacity (*q*_dis_) plotted vs cycle number of cells operated at 25 °C. Cells were cycled at 0.05C, 0.1C, 0.25C, 0.5C, and 1C for five cycles each, followed by long-term cycling at a consistent rate of 0.1C.

^PEC‑Si^SEB^PEC‑LNO^ shows better performance than ^pSi^SEB^pLNO^ across low and middle C-rates (Figure S16) and in the cycling test (Figure 3b). In the first cycle at 0.05C, ^PEC‑Si^SEB^PEC‑LNO^ shows an improved *q*_dis_ ≈ 172 mAh·g^–1^, which is 21 mAh·g^–1^ more than ^pSi^SEB^pLNO^ (151 mAh·g^–1^). After 200 cycles, the retention (based on the sixth and 200th cycles) of ^PEC‑Si^SEB^PEC‑LNO^ is 56%, about double that of ^pSi^SEB^pLNO^ (29%).

### 2.5 Rate Capability of ^LiIn^SEB^LNO^ with PEC and PC Coating on LNO

To corroborate the impact of the PEC coating on cycling performance at the LNO cathode and silicon anode, ^LiIn^SEB^PEC‑LNO^ and ^LiIn^SEB^PEC‑Si^ half-cells are investigated next (Figure 4a). Pellet-type half-cells with the configuration SS|LiIn|LPSCl|LNO|VGCF|SS (^LiIn^SEB^LNO^, an area-specific capacity of 2.1 mAh·cm^–2^ based on 200 mAh·g^–1^ of LNO, an area-specific mass loading of 10.6 mg·cm^–2^ based on LNO) compare the rate capabilities of pLNO-, PC-LNO-, and PEC-LNO-coated cathodes, denoted as ^LiIn^SEB^pLNO^, ^LiIn^SEB^PC‑LNO^, and ^LiIn^SEB^PEC‑LNO^, respectively. To focus on the impact of CEI degradation, we prevent contact loss in ^LiIn^SEB^LNO^ by applying 60 MPa during cycling and using single-crystal LNO to reduce particle fracture.^15,25^

**Figure 4:**
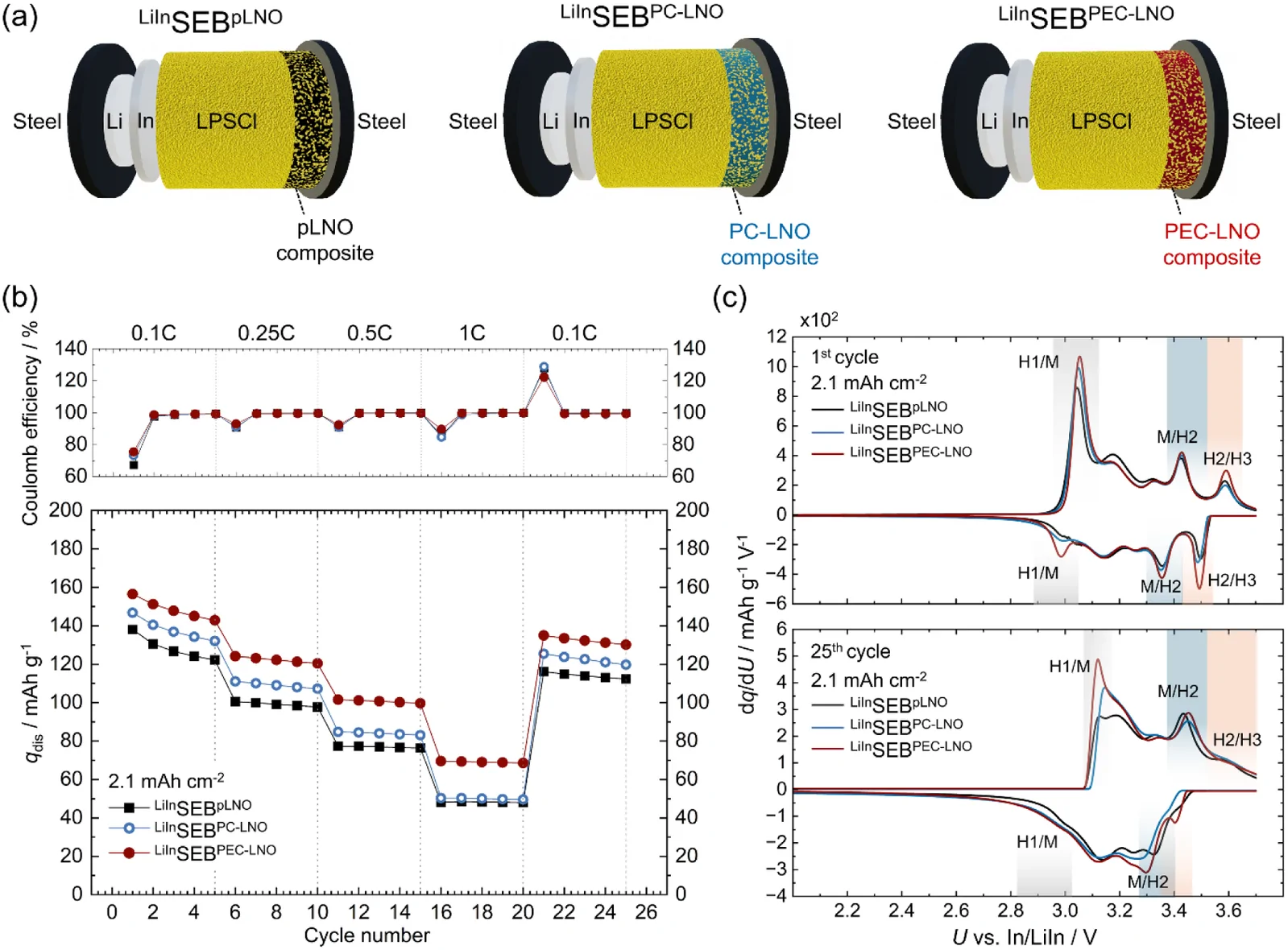
Rate capability of ^LiIn^SEB^LNO^ cells (2.1 mAh·cm^–2^, 10.6 mg·cm^–2^ of LNO), including ^LiIn^SEB^pLNO^, ^LiIn^SEB^PC‑LNO^, and ^LiIn^SEB^PEC‑LNO^, evaluated using pellet-type cells at 25 °C. (a) Schematic representations of the respective cell configurations. (b) Coulomb efficiency (top) and *q*_dis_ (bottom) at various C-rates (0.1C, 0.25C, 0.5C, 1C, and back to 0.1C). (c) d*q*/d*U* plots for the first (top) and 25th (bottom) cycles at 0.1C, highlighting the characteristic LNO phase transitions (H1 ↔ M↔ H2 ↔ H3).

Figure 4b shows that ^LiIn^SEB^PEC‑LNO^ significantly improves *q*_dis_ compared to ^LiIn^SEB^PC‑LNO^ and ^LiIn^SEB^pLNO^ across all C-rates. The galvanic plots of the first and final cycles are shown in Figure S17. In the first cycle at 0.1C, ^LiIn^SEB^pLNO^ delivers 140 mAh·g^–1^ with 67% Coulomb efficiency, ^LiIn^SEB^PC‑LNO^ gives 148 mAh·g^–1^ with 73%, and ^LiIn^SEB^PEC‑LNO^ achieves 157 mAh·g^–1^ with 76%. The improved performance of ^LiIn^SEB^PEC‑LNO^ compared with ^LiIn^SEB^PC‑LNO^ is related to the higher ionic conductivity and flexibility of the PEC coating compared with the rigid PC coating. Two primary factors affecting the first-cycle Coulomb efficiency are the diffusion within the LNO bulk materials^59^ and interfacial degradation at the CEI.^60^ As the coating mainly alters surface rather than bulk properties, the improved first-cycle *q*_dis_ and Coulomb efficiency for ^LiIn^SEB^PEC‑LNO^ are due to reduced CEI degradation.

Figure 4c shows that ^LiIn^SEB^PEC‑LNO^ exhibits a similar differential capacity (d*q*/d*U*) with respect to potential (*U*) of M/H2 (≈3.4 V vs In/LiIn) as both ^LiIn^SEB^PC‑LNO^ and ^LiIn^SEB^pLNO^ in the first charge cycle. However, in the first discharge and 25th cycles, ^LiIn^SEB^PEC‑LNO^ demonstrates better d*q*/d*U* of M/H2 than ^LiIn^SEB^PC‑LNO^ and ^LiIn^SEB^pLNO^. The M/H2 is majorly affected by active mass (*m*_act_) utilization,^25^ which can decrease if LNO becomes isolated due to contact loss or an insulating CEI.^25^ The improved d*q*/d*U* of M/H2 in the first discharge cycle indicates higher *m*_act_ utilization in ^LiIn^SEB^PEC‑LNO^ compared to ^LiIn^SEB^pLNO^ and ^LiIn^SEB^pLNO^. This improvement may result from the PEC coating reducing insulating CEI formation, such as −[S]_*n*_–,^17,18^ improving first-cycle Coulomb efficiency. Notably, these insulating CEI products dominate in the first cycle but diminish thereafter.^60^

On the other hand, ^LiIn^SEB^PEC‑LNO^ has improved d*q*/d*U* at H1/M (≈3 V vs In/LiIn)^61^ compared to ^LiIn^SEB^pLNO^ in the first and 25th cycles, with ^LiIn^SEB^PC‑LNO^ exhibiting intermediate performance. The H1/M transition is affected by both *m*_act_ utilization and kinetic limitations.^25^ A partially conductive, rather than fully insulating, CEI can contribute to kinetic limitations.^25^ During the first charging process, as M/H2 related to *m*_act_ utilization is similar across all samples, ^LiIn^SEB^PEC‑LNO^ improves the interface kinetics of the CEI during the H1/M transition. Subsequent cycle improvement of H1/M stems from a combined influence of enhanced *m*_act_ utilization and interfacial kinetics. After the H1/M and M/H2 transitions, the improved H2/H3 (≈3.6 V vs In/LiIn) is observed for ^LiIn^SEB^PEC‑LNO^ in the first and 25th cycles. The improved H2/H3 suggests reduced oxygenated reactions at the CEI over cycling, affecting both *m*_act_ utilization and kinetics.^24,25^ In conclusion, ^LiIn^SEB^PEC‑LNO^ shows the best performance of all tested SEBs, mainly due to reduced CEI degradation. This observation is further discussed in Section 2.7 (XPS and TOF-SIMS), where it is shown that the decomposition of LPSCl is significantly suppressed in ^LiIn^SEB^PEC‑LNO^ compared to ^LiIn^SEB^pLNO^.

### 2.6 Cycling Stability of ^LiIn^SEB^LNO^ with the PEC Coating on LNO

^LiIn^SEB^PEC‑LNO^ and ^LiIn^SEB^pLNO^ using the same cell setups as in the rate capability test, evaluated LNO cathode cycling performance under 0.1C at 25 °C. EIS measurements were taken at 3.15 V vs In/LiIn during the first, second, 50th, and 100th cycles by the following steps. First, cells were charged to 3.15 V vs In/LiIn, followed by chronoamperometry at 3.15 V vs In/LiIn until the current decreased to 2% of 0.1C. This step ensured a consistent SOC.^43,62^ EIS was then performed from 1 MHz to 100 μHz to evaluate the cathode resistance (*R*_cathode_). 3.15 V vs In/LiIn ensured EIS was conducted at low *R*_cathode_ while avoiding degradation at higher potentials.^63,64^ Subsequently, cell charging was continued to 3.7 V vs In/LiIn, relaxed for 2 h, then discharged to 2.0 V vs In/LiIn with another 2 h relaxation. Open circuit potential (OCP) after 2 h relaxation determined *m*_act_.^25,43^ Figure 5a displays the first-cycle galvanic plots for both SEBs, with chronoamperometry and EIS measurements marked.

**Figure 5:**
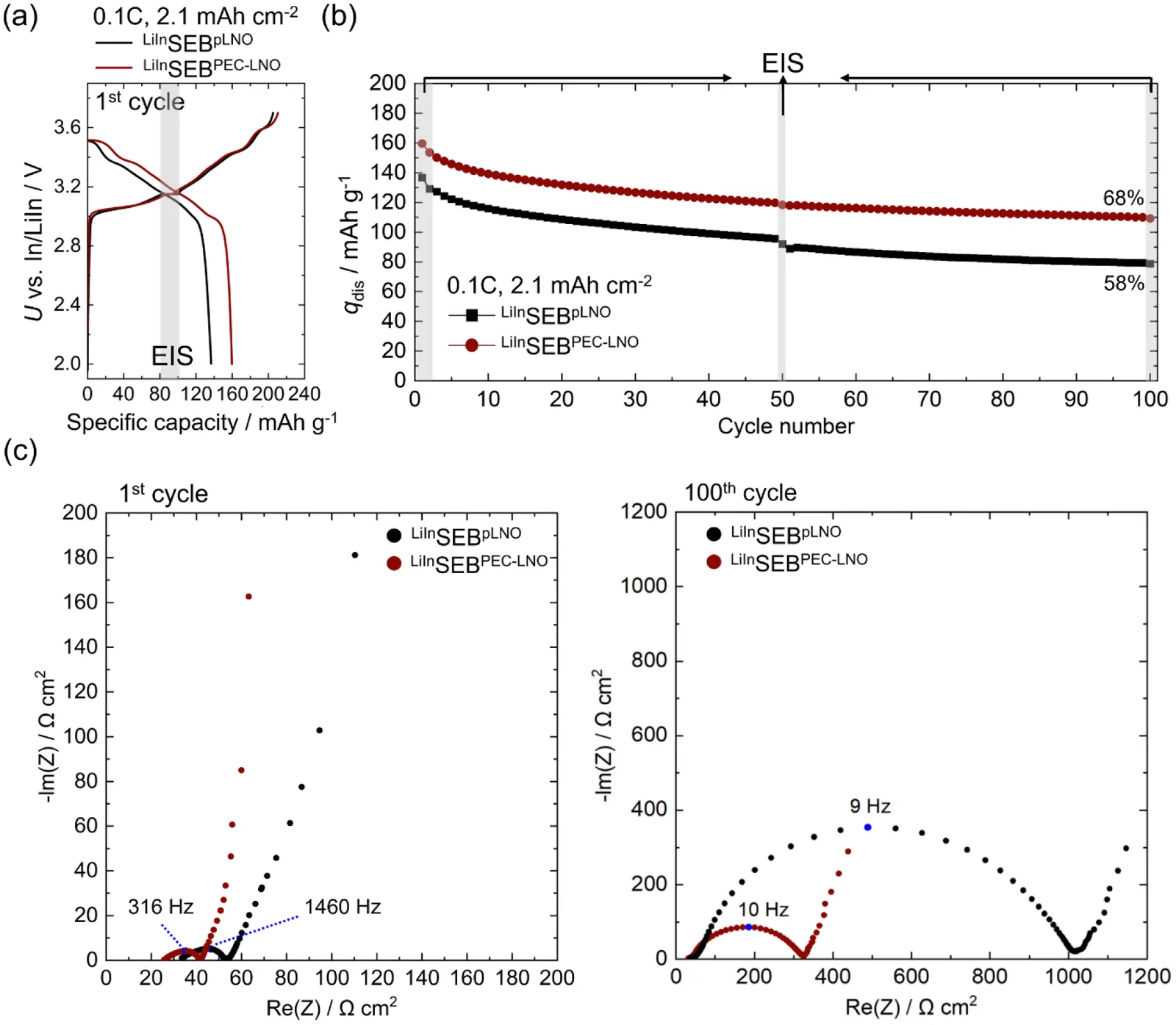
Cycling performance of ^LiIn^SEB^LNO^ cells (2.1 mAh·cm^–2^, 10.6 mg·cm^–2^ of LNO), including ^LiIn^SEB^pLNO^ and ^LiIn^SEB^PEC‑LNO^, tested at 0.1C at 25 °C. EIS and chronoamperometry were performed at 3.15 V vs In/LiIn during the first, second, 50th, and 100th cycles. (a) Galvanostatic profile of the first cycle: the cells were charged to 3.15 V vs In/LiIn, followed by the chronoamperometry (gray area) and EIS. Subsequently, cells were charged to 3.7 V vs In/LiIn, allowed to relax for 2 h, discharged to 2.0 V vs In/LiIn, and relaxed again for 2 h. (b) Cycling stability presented as *q*_dis_ vs cycle number. (c) Nyquist plots of the first and 100th cycles (the apex frequency for both cells is highlighted in blue).

Figure 5b shows that for the first and 100th cycles, ^LiIn^SEB^PEC‑LNO^ has a higher *q*_dis_ and capacity retention (first: 160 mAh·g^–1^, 100th: 109 mAh·g^–1^, retention: 68%) than ^LiIn^SEB^pLNO^ (first: 137 mAh·g^–1^, 100th: 79 mAh·g^–1^, retention: 58%). After 100 cycles, there is no contact loss or LNO particle cracking in both SEBs, as investigated via ion beam milling and SEM (see Figure S18). As a result, the improved performance of ^LiIn^SEB^PEC‑LNO^ can be attributed to mitigated CEI degradation. Additionally, to demonstrate the applicability of the PEC coating strategy to NCM-based cathodes, PEC-coated NCM90 is cycled using an LPSCl catholyte, a Li_5.5_PS_4.5_Cl_0.8_Br_0.7_ (LPSClBr, 9.1 mS·cm^–1^)^65^ separator layer, and a LiIn anode. LPSClBr is used to enhance separator ionic conductivity, while the same LPSCl catholyte was retained to maintain comparable CEI chemistry with LiIn|SE|LNO and LiIn|SE|NCM90 cells. As shown in Figure S19, the PEC coating improves the rate capability and cycling performance of NCM90.

Figure S20 presents *m*_act_ results for ^LiIn^SEB^LNO^ obtained via open-circuit potential (OCP) measurements. The detailed calculation is reported in our previous publication.^25,43^ Generally, the OCP-SOC relationship reveals the lithium-ion content (i.e., the “*x*” in Li_*x*_NiO_2_). To establish a reference for OCP vs SOC, an ^Li^LEB^pLNO^ cell composed of a lithium anode and pLNO is utilized (see Figure S21). The LE helps mitigate capacity loss by minimizing degradation at the CEI, contact loss, and cracking.^66^ Based on the OCP-SOC reference, the specific discharge capacity at a given SOC (*q*_SOC_) can be determined. According to eq 1, *m*_act_ is calculated by the measured discharge capacity (*Q*_meas_) and *q*_SOC_.^25,43^

1 $$m_{\mathrm{act}}\left(g\right)=\frac{Q_{\mathrm{meas}}\;\left(\mathrm{mAh}\right)}{q_{\mathrm{SOC}}\;\left(\frac{\mathrm{mAh}}{g}\right)}$$

Ideally, the SEB should have a *m*_act_ of 8.3 mg. In the first cycle, ^LiIn^SEB^PEC‑LNO^ shows a higher *m*_act_ of 8.1 mg than ^LiIn^SEB^pLNO^ (7.7 mg). The difference between the ideal and actual *m*_act_ arises from cathode distribution inefficiencies and SEB internal resistance. The improved first-cycle *m*_act_ of ^LiIn^SEB^PEC‑LNO^ aligns with the better M/H2 results in the rate capability test (see Figure 4), confirming that the PEC coating can reduce the formation of an insulating CEI in the first cycle, such as −[S]_*n*_–.^17,18^ However, after 100 cycles, both ^LiIn^SEB^PEC‑LNO^ and ^LiIn^SEB^pLNO^ have similar *m*_act_ retention ≈74%, indicating that the mitigation of the kinetic limitations at the CEI plays a role in ^LiIn^SEB^PEC‑LNO^ during cycling, thereby giving rise to improved capacity retention. Oxygenated side reaction products such as Li_2_SO_4_ and Li_3_PO_4_ with ionic conductivities ≈10^–4^∼10^–5^ S·cm^‑1^ ^67,68^ do not directly isolate LNO particles, resulting in similar *m*_act_ retention in both cells. As a result, improved capacity retention in ^LiIn^SEB^PEC‑LNO^ arises from improved kinetic limitations at the CEI during cycling.

To verify this CEI-stabilization mechanism, EIS results are shown in Figure 5c for the first and 100th cycles and in Figure S22 for the second and 50th cycles. *R*_cathode_, including charge transfer resistance (*R*_ct_) and electronic (*R*_ele_) and ionic (*R*_ion_) transport resistances, can be influenced by the effective contact area at the CEI (impacting *m*_act_)^25,43^ and CEI kinetics.^69^ Although *R*_ct_ is normally used to assess CEI degradation over cycling, the Gerischer-type impedance makes it difficult to accurately separate *R*_ct_ from *R*_ele_ and *R*_ion_ in the cathode composite using the transmission line model.^70^ Distribution of relaxation times (DRT) analysis (Figure S23) is used to deconvolute these processes. Based on literature reported, first-cycle *R*_cathode_ can be assigned to approximately 10^2^∼10^5^ Hz, with *R*_ct_ appearing at 10^2^∼10^3^ Hz.^71,72^ *R*_ele_ and *R*_ion_ manifest as two asymmetric shoulders on the high-frequency side (10^3^∼10^5^ Hz).^71,73^ Additionally, the LiIn/LPSCl interphase impedance appears at about 0.1∼10 Hz, with the Warburg diffusion process around 0.1 Hz.^71,74^ Minnmann et al., using the same LPSCl separator and LiIn alloy, also reported that the LiIn/LPSCl interphase impedance during the first cycle should be below 10 Ω·cm^2^ at ≈5 Hz apex frequency.^75^

During cycling, isolating *R*_cathode_ in the DRT becomes increasingly difficult, although *R*_ct_ still clearly indicates a shift toward lower frequencies. This shift arises because cathode degradation shifts the characteristic frequency of *R*_ct_ from the high-frequency region toward lower frequencies, where it increasingly overlaps with the LiIn/LPSCl interphase impedance. However, based on the literature reported, *R*_cathode_ increases much more significantly than the LiIn/LPSCl interphase impedance during cycling,^76,77^ as the LiIn alloy exhibits well-established kinetic stability with LPSCl.^17,58^ Therefore, *R*_cathode_ can be estimated from the difference between the two Re­(Z)-axis intercepts in the Nyquist plot.

The similar apex frequency in ^LiIn^SEB^PEC‑LNO^ and ^LiIn^SEB^pLNO^ indicates that impedance growth during cycling for both cells stems from the same CEI degradation process.^24,66^ In the first cycle at 3.15 V vs In/LiIn, the *R*_cathode_ of ^LiIn^SEB^PEC‑LNO^ is 11 Ω·cm^2^, 7 Ω·cm^2^ lower than that of ^LiIn^SEB^pLNO^ (18 Ω·cm^2^). This aligns with the better *m*_act_ utilization for ^LiIn^SEB^PEC‑LNO^ than ^LiIn^SEB^pLNO^ in the initial cycle. After 100 cycles, the *R*_cathode_ for ^LiIn^SEB^pLNO^ (939 Ω·cm^2^) shows a more pronounced increase compared to ^LiIn^SEB^PEC‑LNO^ (218 Ω·cm^2^). As a result, the PEC coating mitigates the increase in *R*_cathode_ during cycling. Given the similar *m*_act_ retention of ^LiIn^SEB^PEC‑LNO^ and ^LiIn^SEB^pLNO^ after 100 cycles, EIS results indicate that the PEC coating can improve CEI kinetics during cycling, likely by suppressing oxygenated degradation at CEI.

### 2.7 Analysis of Interfacial Degradation in ^LiIn^SEB^LNO^ by XPS and TOF-SIMS

XPS is used to semiquantitatively analyze the CEI of ^LiIn^SEB^pLNO^ and ^LiIn^SEB^PEC‑LNO^, focusing on SS|LPSCl, VGCF|LPSCl, and LNO|LPSCl interfaces. The S 2p region contains two signals associated with LPSCl: PS_4_^3–^ at 161.5 eV and S^2–^-containing species at approximately 160.4 eV, which may originate from the LPSCl lattice and/or residual precursor phases such as Li_2_S in pLPSCl. Because these signals have closely overlapping binding energies, they cannot be unambiguously resolved by S 2p XPS alone.^78,79^ Additionally, −[S]_*n*_– are major CEI degradation products. Therefore, two sulfur components are included in the S 2p spectrum to give a better fitting after cycling: bridging sulfur species (S_*x*_), typically observed at binding energies of 163.1∼163.9 eV, and terminal sulfur species (S_*x*_^T^), which appear at 161.7∼162.3 eV.^80^ On the other hand, the P 2p spectra show signals at 131.9 eV, assigned to PS_4_^3–^ in LPSCl, and at 132.9∼133.2 eV, attributed to oxidized phosphorus species (P_ox_).^78^ However, the Cl 2p spectrum is excluded because LiCl remains stable once formed,^18^ and there are minimal binding energy differences between Cl^–^ in LiCl and LPSCl, making it difficult to distinguish these signal contributions.^81^

Figure S24 shows that the XPS spectra for S 2p and P 2p before cycling are similar for ^LiIn^SEB^pLNO^ and ^LiIn^SEB^PEC‑LNO^. After cycling, ^LiIn^SEB^pLNO^ exhibits more degradation products than ^LiIn^SEB^PEC‑LNO^. In particular, the −[S]_*n*_– species (S_*x*_ and S_*x*_^T^) account for ∼59 mol % in the S 2p spectrum of ^LiIn^SEB^pLNO^, compared with ∼38 mol % for ^LiIn^SEB^PEC‑LNO^. A similar trend is observed in the P 2p spectra, where oxidized phosphorus species (P_ox_) reach ∼72 mol % for ^LiIn^SEB^pLNO^ but ∼58 mol % for ^LiIn^SEB^PEC‑LNO^. However, sulfoxides (SO_*x*_^–^, e.g., Li_2_SO_4_ at ∼169 eV),^82^ which are oxygenated CEI products formed during oxygen evolution from LNO,^25,26^ are not evident from the XPS S 2p spectra. This is likely due to the detection limit of XPS, consistent with the results reported by Walther et al.^83^ Therefore, ToF-SIMS is used to analyze LPSCl/LNO interfacial degradation because it is several orders of magnitude more sensitive than XPS for readily ionized fragments and can semiquantitatively detect reaction products below the XPS detection limit.^83^

TOF-SIMS semiquantitatively^84^ detects CEI decomposition product fragments in the cathode composite of ^LiIn^SEB^LNO^ (see Figure 6), including −[S]_*n*_– (S_4_^–^), phosphoxides (PO_*x*_^–^, including PO^–^, PO_2_^–^, and PO_3_^–^), and sulfoxides (SO_*x*_^–^, including SO^–^, SO_2_^–^, and SO_3_^–^), before and after cycling.^78,83,85,86^ To examine interfaces within the cathode composite (SS|LPSCl, VGCF|LPSCl, and LNO|LPSCl),^85^ wedges are created by gradually increasing the sputter dose density from left (close to SS|LPSCl) to right across a sputtered rectangle. This approach allows visualization of the depth distribution of degradation products in the surface spectrum, as schematically shown in Figure S25. Following the formation of wedge-shaped craters, TOF-SIMS mappings of the cathode composite before and after cycling are displayed as linear scans. Due to the consistent overlap of LNO intensities (NiO_2_^–^) across samples (Figure S26), the average NiO_2_^–^ intensity is used in each figure to mark where sputtering reaches the LNO particles.

**Figure 6:**
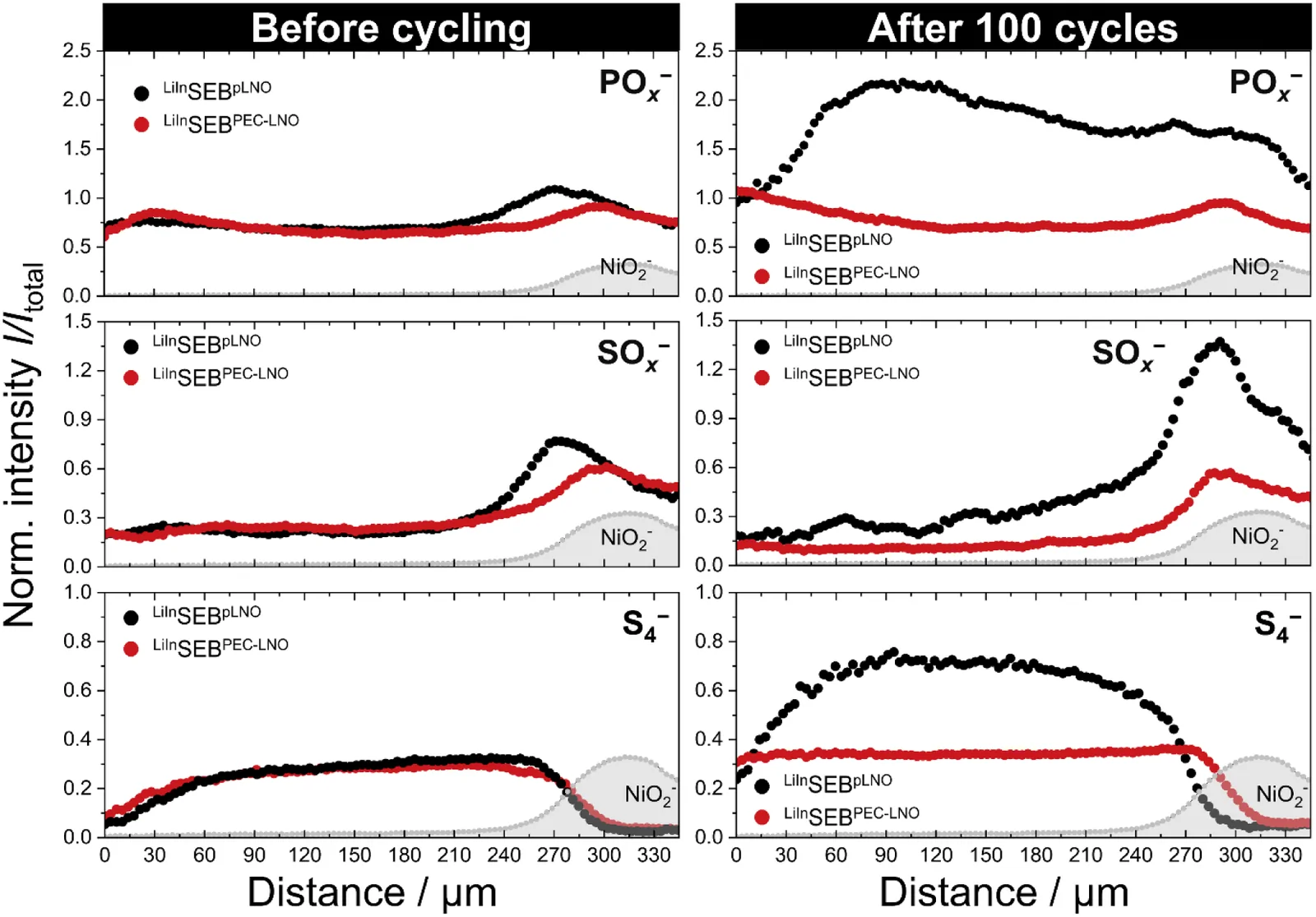
TOF-SIMS analysis performed on wedge-shaped craters of cathode composites before (left) cycling and after (right) 100 cycles. Phosphoxide fragments (PO*_x_* ^–^, including PO^–^, PO_2_^–^, and PO_3_^–^) are shown at the top, sulfoxide fragments (SO*_x_* ^–^, including SO^–^, SO_2_^–^, and SO_3_^–^) in the middle, and −[S]*_n_*– fragments (S_4_^–^) are at the bottom. The average NiO_2_^–^ signal is indicated by the gray area in each plot. All fragment intensities are normalized to the total ion intensity.

Before cycling, S_4_^–^ overlaps nicely across sputtering regions for both ^LiIn^SEB^PEC‑LNO^ and ^LiIn^SEB^pLNO^. However, PO_*x*_^–^ and SO_*x*_^–^ show slightly higher intensity near the LNO particles in ^LiIn^SEB^pLNO^ than in ^LiIn^SEB^PEC‑LNO^ before cycling. Notably, the contact between LNO (fully lithiated) and LPSCl during sample preparation can already lead to the formation of PO_*x*_^–^ and SO_*x*_^–^.^83^ Therefore, the PEC coating helps suppress such chemical reactions during sample preparation.

After 100 cycles, ^LiIn^SEB^pLNO^ has higher S_4_^–^, SO_*x*_^–^, and PO_*x*_^–^ intensity than ^LiIn^SEB^PEC‑LNO^ across the wedge profile. This indicates that the PEC coating can mitigate CEI degradation between LNO and LPSCl during cycling. In addition, the PEC coating can inhibit the first-cycle side reaction that forms −[S]_*n*_–, aligning with the ESW results measured by CV and supporting the first-cycle *m*_act_ result in the rate capability and cycling stability test. Interestingly, the SO_*x*_^–^ intensity is low at the start of sputtering (near SS|LPSCl) and increases as sputtering reaches the LNO surface. In contrast, PO_*x*_^–^ intensity is observed across the entire range. This result aligns with Walther et al., who observed similar outcomes near the SS|LPSCl interface.^85^ Overall, TOF-SIMS and XPS confirm that the PEC coating improves CEI stability in comparison to uncoated LNO.

On the other hand, detecting PEC-derived signals by XPS, particularly in the N 1s and F 1s regions, is challenging because the PEC coating layer is extremely thin and its concentration is below the detection limit of the instrument. Moreover, X-ray exposure during XPS may induce LiF formation from LiTFSI decomposition, potentially complicating the F 1s analysis.^87^ TOF-SIMS shows that the PEC-related signals in ^LiIn^SEB^PEC‑LNO^, including characteristic TFSI^–^ fragments, remain similar before and after cycling. However, LiF_2_^–^ is not observed before and after cycling, suggesting no obvious PEC degradation. Nevertheless, it should be noted that the TFSI-rich PEC layer may still promote the in situ formation of a LiF-rich interphase with LPSCl, which has been demonstrated to improve CEI stability.^88^

### 2.8 Rate Capability of ^LiIn^SEB^Si^ with the PEC Coating on Si

To study the PEC coating’s influence on the silicon anode, pellet-type half-cells with the configuration of SS|LiIn|LPSCl|Si|VGCF|SS (^LiIn^SEB^Si^, an area-specific capacity of 4.5 mAh·cm^–2^ based on 3500 mAh·g^–1^ of silicon, an area-specific mass loading of 1.3 mg·cm^–2^ based on silicon) were used to compare the rate capability of pSi, PC-Si, and PEC-Si composites, denoted as ^LiIn^SEB^pSi^, ^LiIn^SEB^PC‑Si^, and ^LiIn^SEB^PEC‑Si^, respectively (see Figure 7a). The rate capability test was conducted under 60 MPa at different C-rates, with first discharging and then charging: 0.05C, 0.1C, 0.2C, 0.5C, 1C, and then returned to 0.1C. While this pressure may not entirely prevent contact loss from silicon’s 300% volume expansion,^89^ comparing the interfacial stability between coated and pristine silicon under the same conditions remains valuable.

**Figure 7:**
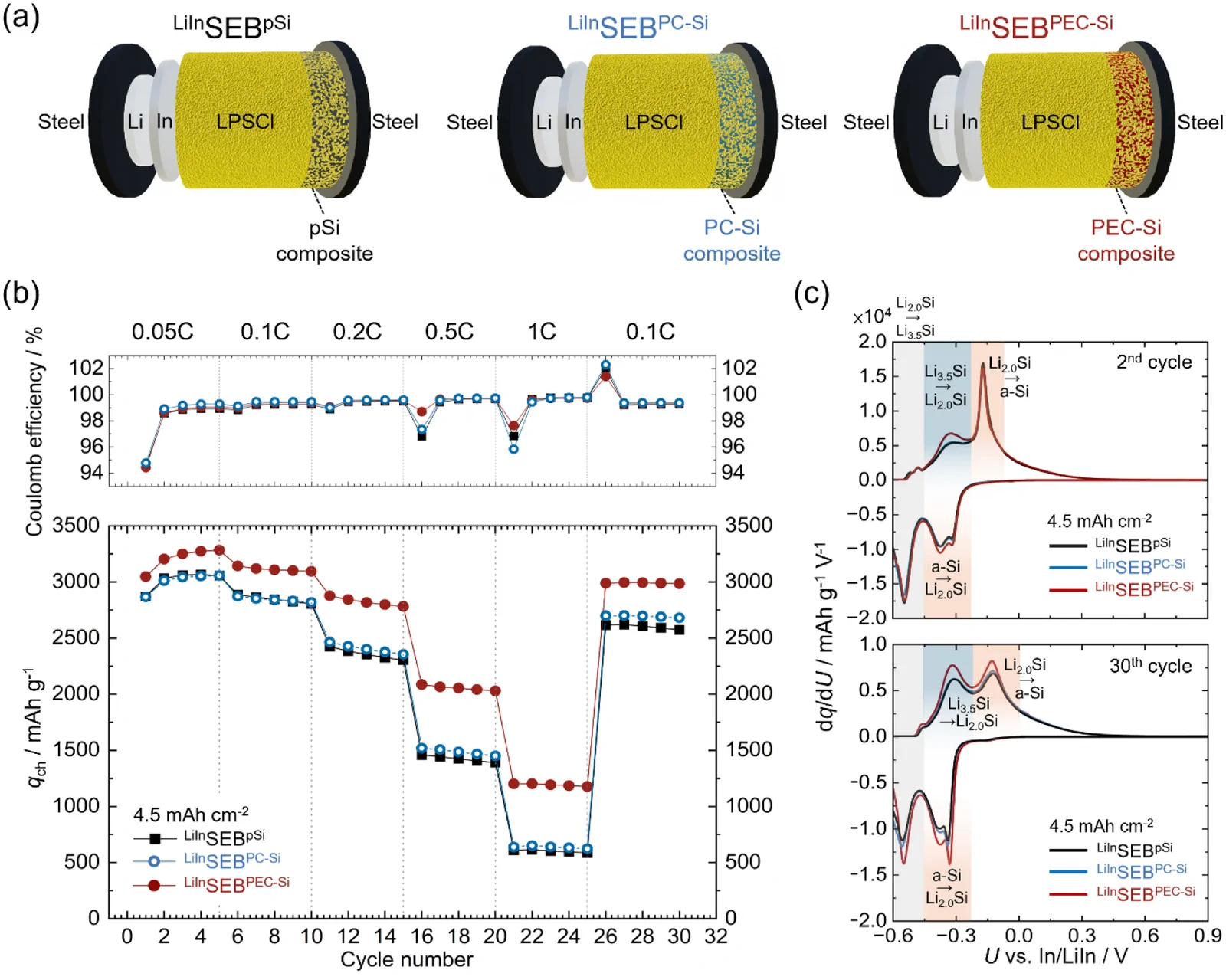
Rate capability of ^LiIn^SEB^Si^ (SS|LiIn|LPSCl|Si|VGCF|SS, 4.5 mAh·cm^–2^ and 1.3 mg·cm^–2^ of silicon), including ^LiIn^SEB^pSi^, ^LiIn^SEB^PC‑Si^, and ^LiIn^SEB^PEC‑Si^, evaluated using pellet-type cells. (a) Schematic illustration of the cell configurations. (b) Coulomb efficiency (top) and *q*_ch_ (bottom) at various C-rates (0.05C, 0.1C, 0.2C, 0.5C, 1C, and back to 0.1C). (c) d*q*/d*U* plots for the second cycle at 0.05C (top) and 30th cycle at 0.1C (bottom), highlighting the characteristic silicon phase transitions (a-Si → Li_3.5_Si ↔ Li_2.0_Si ↔ a-Si).

Figure 7b shows that ^LiIn^SEB^PEC‑Si^ improves the specific charge capacity (*q*_ch_) compared to ^LiIn^SEB^PC‑Si^ and ^LiIn^SEB^pSi^ across all C-rates. Figure S27 shows the galvanic plots of the first, second, and 30th cycles. However, ^LiIn^SEB^PC‑Si^ and ^LiIn^SEB^pSi^ exhibit similar rate capabilities. In the first cycle at 0.05C, ^LiIn^SEB^pSi^ delivers a *q*_ch_ of 2873 mAh·g^–1^, ^LiIn^SEB^PC‑Si^ gives 2867 mAh·g^–1^, and ^LiIn^SEB^PEC‑Si^ achieves 3048 mAh·g^–1^. The first-cycle Coulomb efficiency for all ^LiIn^SEB^Si^ remains consistent at around 95%, significantly higher than that of ^LiIn^SEB^LNO^ (67∼76%), which varies more widely between pristine and coated samples. This is because the main factor contributing to the first-cycle efficiency loss in ^LiIn^SEB^Si^ appears to be contact loss^32^ and/or lithium trapping in the silicon bulk material.^90^ However, a 1∼3 nm coating is not expected to mitigate contact loss caused by volume changes.

Figure 7c compares the d*q*/d*U* of ^LiIn^SEB^Si^ for the second and 30th cycles at 0.05C, while Figure S28 presents it for the first cycle. d*q*/d*U* highlights the phase transitions from crystalline silicon (c-Si) to Li_3.5_Si during the first discharge cycle below −0.45 V vs In/LiIn, and the subsequent phase transitions between Li_3.5_Si ↔ Li_2.0_Si ↔ amorphous silicon (a-Si) in continued cycles.^91^ During the first-cycle charge process and the second cycle, ^LiIn^SEB^PEC‑Si^ exhibits a more pronounced peak of delithiation from Li_3.5_Si → Li_2.0_Si, outperforming ^LiIn^SEB^PC‑Si^ and ^LiIn^SEB^pSi^, which can be attributed to the improved SEI kinetics and mitigated *m*_act_ loss.^92–94^

Furthermore, by the 30th cycle, ^LiIn^SEB^PEC‑Si^ demonstrates a more improved d*q*/d*U* and less peak shift across all phase transitions, Li_3.5_Si ↔ Li_2.0_Si ↔ a-Si, outperforming both ^LiIn^SEB^PC‑Si^ and ^LiIn^SEB^pSi^. It has been reported in the literature that the Li_2.0_Si ↔ a-Si is influenced by *m*_act_ loss.^92–94^ As a result, this suggests that the PEC coating helps mitigate *m*_act_ loss after 30 cycles. However, as the coating does not address contact loss from volume changes,^40^ the PEC coating may improve *m*_act_ utilization by limiting SEI resistance growth, which otherwise isolates the silicon particle. This observation is further discussed in Section 2.10 (XPS and TOF-SIMS), where it is shown that the decomposition of LPSCl is significantly suppressed in ^LiIn^SEB^PEC‑Si^ compared to ^LiIn^SEB^pSi^.

To further check if the rate capability is influenced by the mass loading in the silicon composite, we increased the area capacity in both ^LiIn^SEB^PEC‑Si^ and ^LiIn^SEB^pSi^ from 4.5 mAh·cm^–2^ to 9 mAh·cm^–2^ by doubling the anode composite amount, as shown in Figure S29. ^LiIn^SEB^Si^ with 9 mAh·cm^–2^ shows decreased rate capability compared to ^LiIn^SEB^Si^ with 4.5 mAh·cm^–2^. However, ^LiIn^SEB^PEC‑Si^ still improves the *q*_ch_ compared to ^LiIn^SEB^pSi^ across all C-rates, which confirms that the PEC coating can improve the rate capability even when the loading of silicon increases.

### 2.9 Cycling Stability of ^LiIn^SEB^Si^ with the PEC Coating on Si

For the first, second, and 103rd cycles, ^LiIn^SEB^pSi^ and ^LiIn^SEB^PEC‑Si^ (4.5 mAh·cm^–2^) are charged at 0.05C to 0 V (vs In/LiIn), followed by chronoamperometry at 0 V until the current drops to 2% of 0.05C. Chronoamperometry ensures stable measurements at a fixed SOC with sufficient ionic and electronic conductivity.^32^ EIS is then used to evaluate silicon anode composite resistance (*R*_anode_) from 1 MHz to 100 μHz at 0 V. The galvanic plot of the first cycle, including the chronoamperometry and EIS process, is shown in Figure 8a. From the third cycle to the 102nd cycles, ^LiIn^SEB^Si^ is cycled at 0.1C. However, we cannot access the *m*_act_ via OCP due to the potential hysteresis of Si.^95^ This may need further study in the future.

**Figure 8:**
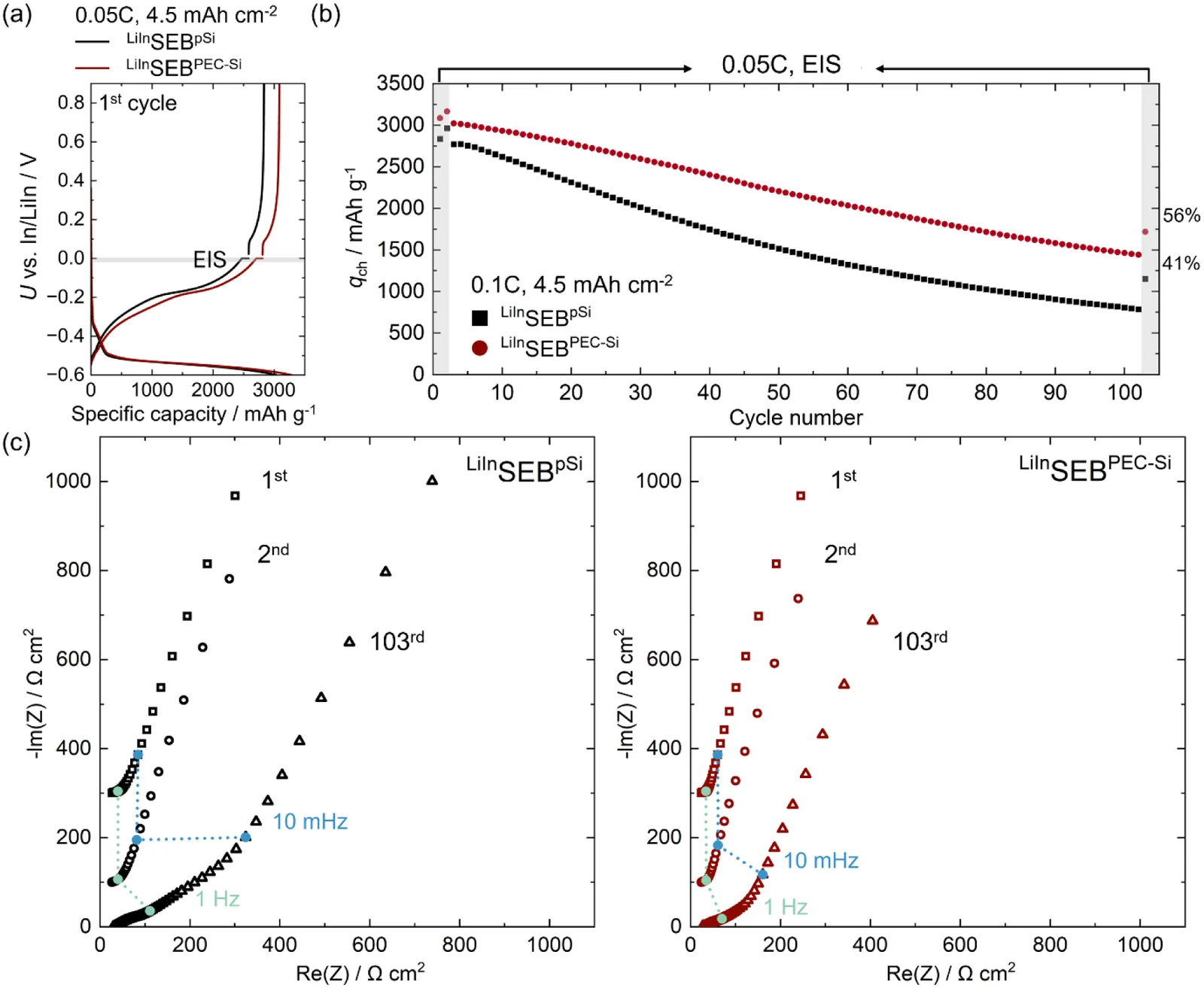
Cycling performance of ^LiIn^SEB^Si^ cells (4.5 mAh·cm^–2^ and 1.3 mg·cm^–2^ of Si), including ^LiIn^SEB^pSi^ and ^LiIn^SEB^PEC‑Si^, tested at 0.1C at 25 °C from the third to the 102nd cycle. EIS measurements were performed at 0.05C at 0 V vs In/LiIn in the first, second, and 103rd cycles. (a) Galvanostatic profiles of the first cycle: cells were discharged to −0.6 V vs In/LiIn, charged to 0 V, followed by a chronoamperometry step (gray area) and EIS, and subsequently recharged to 0.9 V vs In/LiIn. (b) Cycling stability expressed as *q*_ch_ vs cycle number. (c) Nyquist plots of the first and 103rd cycles recorded at 0 V vs In/LiIn.

Figure 8b shows that ^LiIn^SEB^PEC‑Si^ achieves a *q*_ch_ of 3086 mAh·g^–1^ in the first cycle, surpassing ^LiIn^SEB^pSi^ (2834 mAh·g^–1^). After 103 cycles, ^LiIn^SEB^PEC‑Si^ retains 56% of its initial *q*_ch_ at 0.05C, which is more than ^LiIn^SEB^pSi^ (41%), demonstrating that the PEC coating improves cycling stability. Figure S30 presents the cross-section of the anode composites after cycling, examined using ion beam milling combined with SEM after cell disassembly. Contact loss with a gap of approximately 500 nm is observed in both ^LiIn^SEB^PEC‑Si^ and ^LiIn^SEB^pSi^. Although PEC viscoelasticity may help maintain interparticle contact and thereby contribute to cycling stability, this effect is difficult to quantify because contact retention may be altered during cell disassembly. Notably, no fracture within silicon particles is observed after cycling. This may be attributed to the elastic softening effect caused by lithium incorporation into silicon, reducing the hardness (from 10.6 GPa of pure silicon to 1.5 GPa of Li_3.75_Si) and Young’s modulus (from 92 GPa of pure silicon to 12 GPa of Li_3.75_Si).^96^ Since the PEC coating cannot significantly prevent physical contact loss and particle cracking nor alter silicon intrinsic conductivity, its primary benefit likely lies in reducing side reactions at the SEI, thereby enhancing cycling stability.

To verify this SEI-stabilization mechanism, the *R*_anode_ is evaluated by EIS at 0 V vs In/LiIn (see Figure 8c), with further support from DRT analysis to deconvolute electrode processes (see Figure S31). Based on literature reported, the first-cycle *R*_anode_, including *R*_ct_ and *R*_ion_ at the SEI, can be assigned to approximately 1∼10^5^ Hz, with *R*_ct_ appearing at 1∼10^2^ Hz.^97^ *R*_ion_ at the SEI manifests toward the high-frequency side (10^3^∼10^5^ Hz). Additionally, the LiIn/LPSCl interphase impedance, as discussed previously in Section 2.6, appears at about 0.1∼10 Hz with around 5 Hz of apex frequency,^71,74,75^ overlapping with the Warburg diffusion process around 0.1∼1 Hz.^97^

However, accurately extracting *R*_anode_ from EIS by DRT becomes difficult because the Warburg diffusion and the LiIn/LPSCl interphase increasingly overlap with *R*_ct_ during cycling. Nevertheless, direct visual comparison between ^LiIn^SEB^pSi^ and ^LiIn^SEB^PEC‑Si^ remains valid because their postcycling impedance difference is pronounced, while the kinetic stability of LiIn against LPSCl should limit its impedance growth during cycling.^17,58^ After 103 cycles, *R*_anode_, specifically *R*_ct_ based on DRT results, for ^LiIn^SEB^pSi^ increases significantly compared to that for ^LiIn^SEB^PEC‑Si^. As the PEC coating does not mitigate the physical contact loss or the bulk properties of silicon, the impedance results suggest that the PEC coating improves *R*_ct_ at the SEI after 103 cycles.

### 2.10 Analysis of Interfacial Degradation in ^LiIn^SEB^Si^ by XPS and TOF-SIMS

XPS is used to semiquantitatively analyze the SEI of ^LiIn^SEB^pSi^ and ^LiIn^SEB^PEC‑Si^, focusing on the VGCF|LPSCl and Si|LPSCl interfaces after cycling. The SS|LPSCl interface could not be analyzed because part of the silicon composite anode remained attached to the SS after disassembly, leaving only the VGCF|LPSCl and Si|LPSCl interfaces in the pellet for analysis.

Figure 9 shows the XPS spectra for S 2p and P 2p after 103 cycles, indicating the chemical structure of the SEI. Figure S32 shows that the XPS spectra for S 2p and P 2p before cycling are similar for ^LiIn^SEB^pSi^ and ^LiIn^SEB^PEC‑Si^. The Cl 2p spectrum is excluded, as noted in Section 2.7.^18,81^ The S 2p spectra show Li_2_S at 160.4 eV, PS_4_^3–^ at 161.5 eV, and S_*x*_ at 163.2 eV.^18,78^ The P 2p spectra show signals at 131.9 eV (PS_4_^3–^), 132.9∼133.2 eV (oxidized P species, e.g., P_ox_), 130.4 eV (reduced P species,^18^ e.g., elemental P), and 130.1 eV (Li_3_P).^78,81^ Following the ideal reduction of the LPSCl shown in eq 2 ,^17,18^ the ideal SEI side reaction products are Li_2_S and Li_3_P.

2 $${\mathrm{LPSCl}+8\mathrm{Li}}^{+}+{8e}^{-}\to {\mathrm{LiCl}+\mathrm{Li}}_{3}{P+5\mathrm{Li}}_{2}S$$

**Figure 9:**
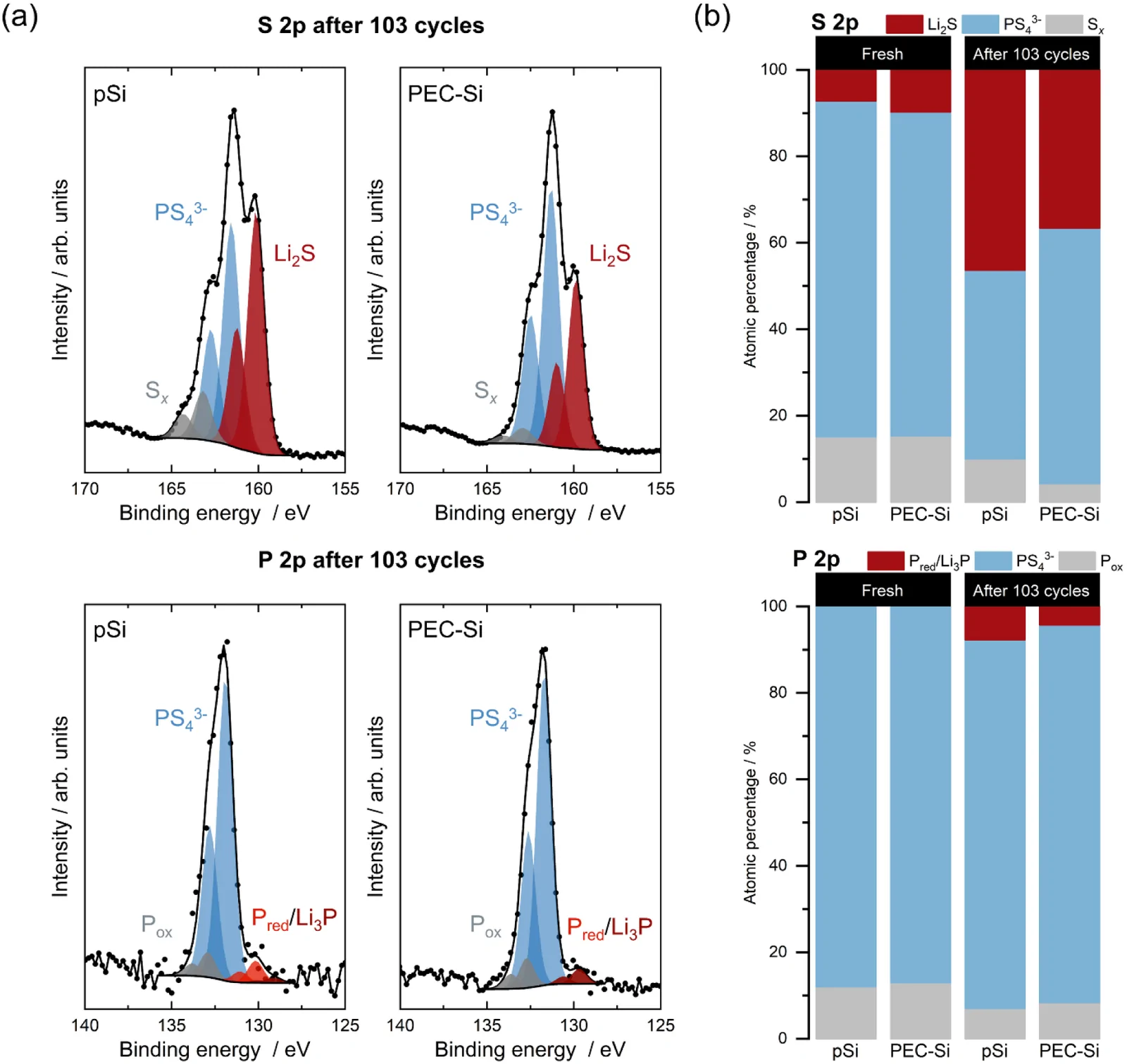
(a) S 2p and P 2p XPS spectra of ^LiIn^SEB^pSi^ (pSi) and ^LiIn^SEB^PEC‑Si^ (PEC-Si) after cycling, highlighting differences in interfacial side-reaction products. (b) Relative ratios of S- and P-containing side-reaction products extracted from the quantitative fitting of XPS spectra.

After 103 cycles, the S 2p spectrum shows that ^LiIn^SEB^pSi^ exhibits a higher Li_2_S content, increasing to 46%, compared with 37% for ^LiIn^SEB^PEC‑Si^. This confirms that the PEC coating effectively mitigates side reactions at the SEI. Interestingly, the P 2p spectrum shows a relatively low Li_3_P ratio after cycling for both cells, warranting further investigation. To support the XPS results, TOF-SIMS (see Figure S33) reveals more Li_2_S (LiS^–^ fragment) for ^LiIn^SEB^pSi^ than for ^LiIn^SEB^PEC‑Si^ after cycling, consistent with the XPS S 2p spectrum. However, Li_3_P is not detected in TOF-SIMS, aligning with the XPS P 2p spectrum. Overall, XPS and TOF-SIMS confirm that the PEC coating can effectively reduce the Li_2_S side reaction product at SEI after 103 cycles.

On the other hand, detecting PEC-derived signals by XPS and TOF-SIMS is even more challenging for ^LiIn^SEB^Si^ than for ^LiIn^SEB^LNO^, because the polymer content in the composite anode is lower than that in the composite cathode due to the lower active material fraction. Nevertheless, the TFSI-rich PEC layer may promote the in situ formation of LiF through reaction with LPSCl or reductive decomposition, and the resulting LiF-rich interphase has been demonstrated to improve the SEI stability.^88,98^

## 3 Conclusion

To improve the stability of both the CEI between nickel-rich CAMs and LPSCl, and the SEI between silicon anodes and LPSCl in solid-state batteries, we introduce a new polymer coating based on a solution-processable PEC applied to electrode particles via spray drying. In this PEC system, PC functions as a coating promoter and mechanical reinforcement component, while PA provides the lithium-ion source and facilitates lithium-ion transport. Owing to the highly delocalized anions present in both PC and PA, the resulting reduction in electrostatic interactions between the oppositely charged macro chains, together with the intentionally nonstoichiometric ion-pair ratio, PEC remains soluble in low-boiling organic solvents upon formation, making it well-suited for wet-coating processes. The PEC further exhibits rubber-like mechanical properties, enabling it to effectively accommodate chemo-mechanical stresses during cycling. High-resolution TEM coupled with STEM-EDX analysis confirms the formation of a uniform ≈1∼3 nm PEC layer on the surface of LNO and silicon particles. In full-cell tests pairing a silicon anode with an LNO cathode, the PEC coating improves capacity retention by 27% under 0.1C after 200 cycles. To isolate the coating’s influence at each interphase, half-cells with LiIn counter electrodes were assembled using coated LNO cathodes or coated silicon anodes. The PEC coating improves capacity retention by 10% on LNO and by 15% on silicon after 100 cycles in half-cells. TOF-SIMS and XPS analyses further verify that the PEC coating mitigates detrimental interfacial side reactions. Overall, these results demonstrate that polymeric surface coatings such as PEC can substantially enhance cycling performance in solid-state batteries, highlighting their value in the development of next-generation electrochemical energy storage systems.

## 4 Experimental Methods

### 4.1 Reagents and Materials

Poly­(ethylene glycol)­methyl ether methacrylate (PEGM, *M*_n_ = 500 g·mol^–1^, Aldrich), 2-cyano-2-propyl benzodithioate (CPDB, 97%, Aldrich), lithium bis­(trifluoromethanesulfonyl)­imide (LiTFSI, 99%, Acros), (vinylbenzyl)­trimethylammonium chloride (VBTA-Cl, 99%, Aldrich), sodium persulfate (Na_2_S_2_O_8_, 98%, Aldrich), ortho-xylene (*o*-xylene, anhydrous 97%, Aldrich), diethyl ether (HPLC grade 99.5%, Acros), acetone (ACS reagent 99.5%, Aldrich, and HPLC grade 99.9%, Acros), dimethylformamide (DMF, HPLC grade 99.5%, Acros), VGCF (Aldrich), silicon (particle size ≈ 1∼5 μm, 99.9% metal basis purity, Alfa Aesar), lithium hydroxide monohydrate (LiOH·H_2_O, >99%, Aldrich), and lithium carbonate (Li_2_CO_3_, 99%, Fischer Chemicals, formerly Alfa Aesar) were used as received. The Spectra/Por 1 (Spectrum Laboratories) dialysis tubing with MWCO 6000–8000 Da was used for polymer dialysis.

For the separator in ^LiIn^SEB^LNO^, LPSCl (NEI Corporation) was used as received. For the separator in ^LiIn^SEB^NCM90^, LPSClBr was synthesized according to the previously reported procedure.^65^ For the catholyte, LPSCl underwent wet milling^75,99,100^ to reduce its particle size. First, 15 g of LPSCl was mixed with 50 mL of *o*-xylene and 80 g of 1 mm ZrO_2_ balls in an 80 mL ZrO_2_ pot. Subsequently, the mixture was wet-milled by a Fritsch premium line Pulverisette 7 mill at 500 rpm for 10 min, followed by a resting period of 10 min. This cycle was repeated six times. After the ball milling was finished, the mixture was centrifuged to get the LPSCl powder. The LPSCl powder was then passed through a 100 μm mesh to remove the residual ZrO_2_ balls. The collected LPSCl was dried at 120 °C and 2 × 10^–2^ mbar for 2 days in the B-585 oven (Buchi Glass Drying Oven, Switzerland). Finally, the LPSCl was manually ground in an agate mortar for 10 min to break up aggregates, then sieved again using a 20 μm mesh. The milling instrument was located outside the glovebox, but sample transfers were protected from air exposure. The rest of the procedure was conducted entirely inside an argon-filled glovebox (LabMaster, MBraun, Garching, Germany), with O_2_ and H_2_O levels maintained below 0.1 ppm.

LiNi_0.9_Mn_0.05_Co_0.05_O_2_ (NCM90, particle size ≈ 3∼5 μm and a BET specific surface area of 0.5∼0.9 m^2^·g^–1^, MSE Supplies) was dried at 120 °C and 2 × 10^–2^ mbar for 2 days in the B-585 oven (Buchi Glass Drying Oven, Switzerland). Lithium 1-[3-(methacryloyloxy)­propylsulfonyl]-1-(trifluoromethanesulfonyl)­imide was prepared in full accordance with the procedures published previously.^55,101^ The resulting crystalline powder was dried at 25 °C and 0.5 mbar overnight and stored under an inert atmosphere in an argon-filled glovebox (MBRAUN MB-UniLAB, H_2_O and O_2_ content <0.5 ppm). 2,2’-Azobis­(isobutyronitrile) (AIBN, initiator, 98%, Aldrich) was recrystallized from methanol. Indium foil (100 μm, ChemPUR GmbH) was punched into circular electrodes with a diameter of 9 mm. Lithium foil (125 μm thick, Albemarle Rockwood Lithium GmbH) was punched into circular electrodes with a diameter of 6 mm.

### 4.2 Synthesis of Single-Crystal pLNO

LNO was prepared in accordance with the procedure published previously,^15^ although with minor changes. In brief, to obtain single-crystal LNO, 10 g of LiOH·H_2_O was manually ground in an agate mortar for 30 min. Subsequently, 16.18 g of NiO was added, and the mixture was ground for an additional 30 min. The resulting powder was loaded into alumina crucibles and annealed under an oxygen flow at 780 °C for 6 h, followed by a second annealing step at 680 °C for another 6 h. Both heating and cooling ramps were set to 100 °C·h^–1^. The resulting black brick-like pLNO precursor was then manually ground in an agate mortar for 20 min. To ensure sufficient lithiation, 6.39 g Li_2_CO_3_ was added and the mixture was manually ground for an additional 20 min. The powder was transferred to alumina crucibles and annealed under an oxygen flow at 750 °C for 40 h, followed by a second step at 680 °C for 20 h, again using heating and cooling ramps of 100 °C·h^–1^. To remove excess Li_2_CO_3_, the pLNO precursor was washed with ice-cooled deionized water (1 g of pLNO precursor with 30 mL H_2_O) by vigorous agitation for 1 min, immediately centrifuged at 4000 rpm for 2 min, and the supernatant was discarded. The washing procedure was repeated three times, with a total water contact time of <12 min. A final rinse with ethanol was performed to aid drying. The resulting pLNO precursor was then dried at 60 °C and 0.5 mbar overnight. After drying, the pLNO precursor was loaded into alumina crucibles and annealed at 680 °C for 3 h under oxygen flow (100 cm^3^·min^–1^). The obtained pLNO was manually ground for 20 min in an agate mortar and sieved through a 45 μm mesh inside an argon-filled glovebox (LabMaster, MBraun, Garching, Germany, with O_2_ and H_2_O levels maintained below 0.1 ppm). The pLNO powder was then sieved a second time using a 20 μm mesh. Yield: 13.34 g. The obtained pLNO showed a 1∼4 μm average particle size as analyzed by SEM (see Figure S14). XRD refinement confirmed its composition and crystal structure (Figure S34), indicating the presence of 8.5 wt % residual Li_2_CO_3_ and 0.6 mol % cation disordering.

### 4.3 Synthesis of Poly­(vinylbenzyl)­trimethylammonium bis­(trifluoromethanesulfonyl)­imide (PC)

PC was synthesized in two steps: (1) free-radical polymerization of VBTA-Cl, followed by (2) ion exchange with LiTFSI. For the first step, a solution of VBTA-Cl (5.00 g, 23.58 mmol) and Na_2_S_2_O_8_ (1.68 g, 7.07 mmol) in 20 mL of deionized water was purged with nitrogen for 15 min to remove dissolved oxygen, then heated to 75 °C and polymerized for 48 h. After cooling to room temperature, the viscous reaction mixture was diluted with deionized water and dialyzed for 7 days. The resulting aqueous polymer solution was concentrated to 50 mL using a rotary evaporator and used directly in the subsequent ion-exchange step.

For the ion exchange, a solution of LiTFSI (7.50 g, 26.13 mmol) in 20 mL of deionized water was added dropwise to the polymer solution under stirring. A white precipitate formed immediately, and the mixture was stirred for an additional 2 h at room temperature. PC was isolated by centrifugation as a white powder, washed three times with deionized water, and dried at 80 °C under vacuum (0.1 mbar) for 72 h in the B-585 oven (Buchi Glass Drying Oven, Switzerland). Yield: 6.88 g (64%). ^1^H NMR (400 MHz, acetone-*d*_6_): δ = 7.32 (br.s., 2H), 6.68 (br.s., 2H), 4.58 (br.s., 2H), 3.12 (br.s., 9H), 1.68 (br.s., 3H) ppm; ^13^C NMR (151 MHz, DMSO-*d*_6_): δ = 138.6–138.4 (m), 136.7, 119.4 (q, *J* = 321.8 Hz), 51.5; ^19^F NMR (564.8 MHz, CDCl_3_): δ = −81.0 (s, CF_3_) ppm; IR (ATR mode): 3044 (w), 2926 (w, ν_CH_), 1615 (w), 1489 (m, ν_CH_), 1478 (m, ν_CH_), 1347 (s, ν_asSO2_), 1327 (s, ν_asSO2_), 1229 (m, ν_CF_), 1176 (vs, ν_sSO2_), 1132 (vs), 1051 (vs, ν_CF_), 988 (w), 976 (m), 921 (w), 886 (m), 587 (w), 828 (w), 789 (m), 763 (w), 740 (m), 719 (w), 653 (w), 611 (s), 599 (s), 569 (vs), 509 (s) cm^–1^; *M*_n_ = 29500 g mol^–1^ g·mol^–1^, *M*_w_/*M*_n_ = 2.96 (by GPC); *T*_g_ = 84 °C (by DSC); *T*_onset_ = 290 °C (by TGA in air).

### 4.4 Poly­[(lithium 1-[3-(methacryloyloxy)­propylsulfonyl]-1-(trifluoromethanesulfonyl)­imide)-*R*-(poly­(ethylene glycol) methyl ether methacrylate)] Synthesis (PA)

The PA was synthesized via random RAFT copolymerization of lithium 1-[3-(methacryloyloxy)­propylsulfonyl]-1-(trifluoromethanesulfonyl)­imide and PEGM using AIBN as the initiator, in accordance with the procedures published previously^55,102^ with the exception that instead of CPAD, CPDB was used as the chain transfer agent. Briefly, a solution of Li monomer (0.50 g, 1.40 mmol), PEGM (3.26 g, 6.50 mmol), CPDB (18.5 mg, 0.083 mmol), and AIBN (2.74 mg, 0.017 mmol, [AIBN]:[CPDB] = 1:5 by mol) in anhydrous DMF (12.0 mL, 11.3 g, [DMF]:[ILM + PEGM] = 3:1 by weight) was transferred to a Schlenk flask equipped with a magnetic stirring bar. The solution was degassed via three freeze–pump–thaw cycles and flushed with argon, whereupon the flask was placed into a bath preheated to 60 °C, and polymerization was carried out at 60 °C for 48 h. The resultant viscous pink polymer solution was diluted with DMF and precipitated into an excess of diethyl ether. PA was collected by decantation of the solvents, then redissolved in acetone and precipitated in an excess of diethyl ether for the second time. The isolated copolymer represented a pink sticky mass that was dried at 60 °C and 0.1 mbar for 24 h in a B-585 oven (Buchi Glass Drying Oven, Switzerland) filled with P_2_O_5_. Yield: 2.40 g (64%). ^1^H NMR (600 MHz, CDCl_3_): δ = 4.09–4.11 (t, 13H, −CO–O–CH_2_), 3.58–3.68 (m, 175H, −CH_2_–O–CH_2_–CH_2_–O−), 3.41 (s, 16H, −O–CH_3_), 3.25 (m, 2H, −CH_2_–SO_2_–N–SO_2_CF_3_), 2.25 (m, 2H, −CH_2_–CH_2_–SO_2_–N–SO_2_CF_3_), 1.92 (m, 13H, −CH_2_–C­(CH_3_)), 0.86–1.04 (m, 19H, −CH_2_–C­(CH_3_)) ppm; ^19^F NMR (564.8 MHz, CDCl_3_): δ = −79.8 (s, CF_3_) ppm; ^7^Li NMR (155.5 MHz, CDCl_3_): δ = −0.75 (s, Li) ppm; FTIR (ATR mode): 2947 (m, ν_C–H_), 2872 (s, ν_C–H_), 1729 (vs, ν_C=O_), 1475 (m), 1455 (m, δ_C–H_, _Alk_), 1404 (m), 1379 (w), 1352 (m, ν_asSO2_), 1326 (w), 1265 (m), 1246 (m), 1227 (m), 1179 (s, ν_C–F_), 1105 (vs, ν_asC–O‑C_), 1055 (s, ν_C–F_), 947 (m), 858 (s, ν_sC–O‑C_), 842 (s), 789 (w), 749 (w) cm^–1^; *M*_n_ = 41000 g mol^–1^, *M*_w_/*M*_n_ = 1.49 (by GPC); *T*_g_ = −54 °C (by DSC); *T*_onset_ = 150 °C (by TGA in air).

### 4.5 Synthesis of PEC

A series of PECs was synthesized by mixing the acetone solutions of two oppositely charged polyelectrolytes, namely PC and PA, at different molar ratios. The typical PEC formation procedure is given by the example of PEC synthesis with a PC PA = 60:40 wt % ratio. A solution of PC (0.6 g, 1.32 mmol of cationic ion pairs, 60 wt % based on the weight of PEC) in 10 mL of acetone was added dropwise to a solution of PA (0.4 g, 0.14 mmol of anionic ion pairs, 40 wt % based on the weight of PEC) in 10 mL of acetone under continuous stirring. The mixture was stirred for 1 h at 25 °C. Afterward, the solution was poured onto a Teflon Petri dish and dried on a hot plate at 60 °C overnight. Further drying was performed in the B-585 oven (Buchi Glass Drying Oven, Switzerland) at 60 °C and 0.1 mbar for 1 week to ensure the complete removal of residual acetone. The polymer was then stored inside an argon-filled glovebox (LabMaster, MBraun, Garching, Germany), with O_2_ and H_2_O levels maintained below 0.1 ppm, to prevent the moisture or humidity influence. *T*_g_ = −14 °C (by DSC); melting temperature (*T*_m_) = −183 °C (by DSC); *T*_onset_ = 230 °C (by TGA in air).

### 4.6 Polymer-Coated AMs

A BUCHI Mini-Spray Dryer B-290 was used to coat AMs (NCM90, pLNO, and pSi) with PC or PEC. Two grams of AMs was dispersed in a solution containing 20 mg of polyelectrolyte (1 wt % relative to the AMs) in 30 mL of acetone to prepare the precursor suspension. The suspension was stirred vigorously for 1 h at room temperature. During spray drying, the inlet temperature was set to 100 °C, the vacuum pumping rate to 37 m^3^·h^–1^ and the nitrogen flow to 40 L·min^–1^. The precursor suspension was fed at 8 mL·min^–1^. These parameters were optimized to achieve a productivity of approximately 70 wt %. Following spray drying, the coated AMs were dried in the B-585 oven (Buchi Glass Drying Oven, Switzerland) at 50 °C and 0.1 mbar for 1 week.

### 4.7 Polymer-Coated VGCF

For the PEC- and PC-coated VGCF, a precursor is prepared by dissolving 10 mg of PC or PEC polymers in 20 mL of acetone. To create a VGCF solution precursor for spray drying, 100 mg of VGCF is sonicated in 20 mL of acetone and then added dropwise to the polymer solution. This mixture is ultrasonicated for 30 min to achieve a homogeneous solution. Subsequently, the coated VGCF is formed using a spray drying process under the same conditions as those used for the coated electrodes. After spray drying, the coated AMs are dried in the B-585 oven (Buchi Glass Drying Oven, Switzerland) at 50 °C and 0.1 mbar for 1 week and then stored in the glovebox.

### 4.8 Nuclear Magnetic Resonance (NMR)

NMR spectra were recorded on an AMX-600 (Bruker, Germany) spectrometer at 25 °C in the indicated deuterated solvent and are listed in ppm. The signals corresponding to the residual protons and carbons of the deuterated solvent were used as an internal standard for ^1^H and ^13^C NMR, respectively. The C_6_F_6_ (−164.9 ppm) and LiNO_3_ were utilized as external standards for ^19^F and ^7^Li, respectively.

### 4.9 Fourier-Transform Infrared Spectroscopy (FTIR)

FTIR spectra were recorded using a Thermo Fisher Scientific iD5 ATR spectrometer over a wavenumber range of 550∼4000 cm^–1^ with a total of 96 scans. All measurements were conducted inside an argon-filled glovebox (LabMaster, MBraun, Garching, Germany), with O_2_ and H_2_O levels maintained below 0.1 ppm to prevent moisture interference.

### 4.10 Gel Permeation Chromatography (GPC)

The number-average molecular weights (*M*_n_) and *M*_w_/*M*_n_ ratios for PC and PA were determined by size exclusion chromatography (SEC) or gel permeation chromatography (GPC). The GPC experiment was performed using a 1200 Infinity gel permeation chromatograph (Agilent Technologies, USA) equipped with a PLgel 5 μm MIXED-D column (Agilent Technologies, USA), a PLgel 5 μm (Agilent Technologies) precolumn, and an integrated refractive index detector. The system was operated at 50 °C and 1.0 mL·min^–1^ flow rate using a 0.1 M Li­(CF_3_SO_2_)_2_N solution in DMF as an eluent. The poly­(methyl methacrylate) standards (EasiVial PM, Agilent Technologies, *M_p_* = 550 ÷ 1558 × 10^3^) were used to perform calibration.

### 4.11 Thermal Gravimetric Analysis (TGA)

TGA was carried out in air on a TGA2 STARe System (Mettler Toledo, Switzerland), applying a heating rate of 5 °C·min^–1^. The *T*_onset_ was determined as the point on the TGA curve at which a significant deviation from the horizontal was observed. The resulting temperature was then rounded to the nearest 5 °C.

### 4.12 Differential Scanning Calorimetry (DSC)

For DSC measurements, all samples were hermetically sealed in Al pans inside the argon-filled glovebox (MBRAUN MB-Labstar, H_2_O and O_2_ content < 0.5 ppm). DSC was performed on a DSC 300 Caliris Select (Netzsch, Germany) differential calorimeter, applying a heating rate of 5 °C min^–1^ (first and second cycles) and 10 °C min^–1^ (third cycle) in the range of −120 to 240 °C. Three to four heating–cooling cycles were carried out for each sample. *T*_g_ and *T*_m_ were determined from the second heating curve.

### 4.13 Rheology

Rheology measurements were performed using a Physica MCR 302 rheometer (Anton Paar, Austria) equipped with a CTD 450 temperature control device and a disposable aluminum plate–plate (diameter: 25 mm, measurement gap: 1 mm) geometry. Measurements were recorded in oscillation mode at an imposed 1% strain amplitude (γ), ensuring that both storage G’ and loss G” moduli were obtained in the linear viscoelastic regime. All measurements were carried out at 25 °C. Tests were repeated at least twice to ensure good repeatability of the results. For PA, which represents a cold-flowing rubber, the material was placed directly between the rheometer plates. For PC and PEC, the following sample preparation procedure was applied: polymer discs (typical diameter × thickness ≈ 20 × 1.5 mm) were prepared using a MeltPrep vacuum compression molding machine (MeltPrep GmbH, Germany). Approximately 0.15 g of polymer was loaded into the mold and heated at 90 °C for 10∼15 min under a vacuum of 0.1 mbar. After heating, the mold was allowed to cool to room temperature, opened, and the polymer disk was gently released.

### 4.14 X-ray Diffraction (XRD)

The pLNO powders were loaded into glass capillaries with an inner diameter of 0.5 mm. XRD measurements were performed on an Empyrean 3 diffractometer (Malvern PANalytical, Netherlands) equipped with a Mo–Kα radiation source in Debye–Scherrer geometry. Data were collected over a 2Θ range of 5°–40° with a step size of 0.007° and a scan rate of 1°·min^–1^.

### 4.15 Scanning Electron Microscopy (SEM)

SEM imaging was performed using a GeminiSEM 560 (Carl Zeiss AG, Germany), operated at an acceleration voltage of 2 kV with a 20 μm aperture. Both backscattered electron and secondary electron images were recorded to characterize the morphology of LNO particles, LNO cathode composites, and silicon anode composites after cycling. LNO particle morphology was examined in powder form, adhered to conductive copper tape. To assess particle cracking in the cycled LNO cathode and Si anode composites, cross-sections were prepared by ion beam milling using a Leica EM TIC 3X (Leica Microsystems, Germany). Milling was conducted at 6 kV and 2.2 mA for 6 h at −100 °C.

### 4.16 (Scanning) Transmission Electron Microscopy ((S)­TEM)

TEM analyses were performed using a double Cs-corrected JEM 2200FS microscope (JEOL Ltd., Japan) operated at 200 kV. The convergence angle in the scanning mode was 15.07 mrad. Powders of the various particles were dispersed onto lacey carbon-coated copper mesh grids and subsequently pumped in a vacuum stand to remove excess solvent and loose material before being inserted into the (S)­TEM column. Bright-field TEM images were acquired using a TVIPS TemCam XF416FS camera (TVIPS GmbH, Germany), while STEM-EDX spectra were collected using a Bruker Nano XFlash detector 5060 (Bruker, Germany). STEM overview images were recorded with an annular detector covering an angular range of 70–280 mrad.

### 4.17 Time-of-Flight Secondary Ion Mass Spectrometry (TOF-SIMS)

All TOF-SIMS measurements were performed on an M6 Plus instrument (IONTOF GmbH, Germany) operated in negative ion mode. Surface spectra were acquired using the NanoProbe 50 LMIG in bunched mode with 30 keV Bi_3_^+^ cluster ions as the primary species. For silicon samples, surface spectra were collected over an area of 200 × 200 μm^2^ with a resolution of 128 × 128 pixels and a dose density of 1 × 10^12^ ions cm^–2^. Five spectra were recorded per sample. For wedge preparation of the cathode composite samples, a DSC/S sputter gun equipped with 2 kV Cs^+^ ions was used on a 300 × 100 μm^2^ area with a maximum dwell time of 200 ms. Subsequent surface spectra of the wedge cross-sections were acquired either at 400 × 400 μm^2^ and 256 × 256 pixels or at 450 × 450 μm^2^ and 512 × 512 pixels, using a dose density of 5 × 10^11^ ions cm^–2^. For data evaluation, ion images were binned to 128 × 128 pixels. A region of interest with a constant I-scale of 350 μm and 20 lines in the *x*-direction was selected. Twenty line scans extracted along the *x*-direction were summed using SurfaceLab 7.4 software and normalized to the total ion intensity for comparison.

### 4.18 X-ray Photoelectron Spectroscopy (XPS)

XPS measurements were performed using a PHI 5000 VersaProbe IV (ULVAC-PHI, Inc., Japan), equipped with an Al Kα source (1486.6 eV). The instrument was operated at 50 W beam power and 15 kV voltage, with a beam diameter of 200 μm. Survey spectra were acquired at a pass energy of 224 eV, while high-resolution spectra were collected at 55 eV. Samples were transferred to the analysis chamber using an argon-filled transfer module to minimize air exposure. Data analysis was carried out using CasaXPS software. Initial calibration of the XPS spectra was performed using the adventitious carbon signal at 284.8 eV to locate the main S 2p component (PS_4_^3–^). Subsequent spectra were calibrated relative to the PS_4_^3–^ signal at 161.6 eV.^85^

### 4.19 Ionic Conductivity

Ionic conductivity was measured by electrochemical impedance spectroscopy (EIS or complex alternating current (AC) impedance analysis) with a VSP potentiostat/galvanostat (Bio-Logic Science Instruments, France). To avoid any influence of moisture or humidity on the conductivity of polymer electrolytes (PC, PA, and PEC), the latter were preliminarily dried at 80 °C and 0.1 mbar for 12 h in the B-585 oven (Buchi Glass Drying Oven, Switzerland) filled with P_2_O_5_ and were transferred under vacuum inside an argon-filled glovebox (MBRAUN MB-Labstar, H_2_O and O_2_ content < 0.5 ppm). The samples were sandwiched between two SS electrodes. The distance between the electrodes was kept equal to 250 μm using a Teflon spacer ring with an inner area of 0.50 cm^2^. Symmetrical SS|polymer|SS cells assembly was clamped into the 2032-coin cell and afterward was taken out from the glovebox. EIS experiments were carried by applying a 10 mV perturbation in the frequency range from 10^–2^ to 2 × 10^5^ Hz. The measurements were carried out in the temperature range from 20 to 100 °C. The temperature was controlled using a programmed M-53 oven (Binder, Germany), where cells were allowed to reach thermal equilibrium for at least 45 min before each test.

### 4.20 Cyclic Voltammetry (CV)

CV was carried out using the cell configuration SS|LiIn|LPSCl|VGCF|SS to evaluate the electrochemical stability. To prepare 99 mg of the LPSCl/VGCF composite, 9 mg of VGCF was mixed with 90 mg of LPSCl and ball-milled at 30 Hz for 1 h using a mini-mill PULVERISETTE 23. For cell assembly, 80 mg of LPSCl was pressed into a 10 mm diameter pellet, serving as the separator within a PEEK insulator. Subsequently, 30 mg of the LPSCl/VGCF composite was pressed onto one side of this separator. On the opposite side, indium metal (100 μm thick, 9 mm diameter) and lithium metal (125 μm thick, 6 mm diameter) foils were placed as the counter electrode, with indium in direct contact with the LPSCl. The complete cell stack was compressed at room temperature under a force of 30 kN for 3 min using an Atlas AutoTouch automatic press. Finally, the cell was secured in an external aluminum frame, applying an additional pressure of approximately 50 MPa.

### 4.21 Open-Circuit Potential (OCP) vs Capacity Reference

To create an open-circuit potential (OCP) vs capacity reference curve, a liquid cell with pLNO as the CAM and lithium metal as the anode was prepared. The cathode slurry was prepared by mixing LNO, Super P carbon, and PVDF binder in a 94:3:3 weight ratio in NMP, with a total solid content of 56 wt %. The mixture was stirred at room temperature for 12 h and subsequently cast onto aluminum foil using a 60 μm doctor blade. The tape was dried at 120 °C and 0.1 mbar for 12 h. For the cell assembly, a cathode with a 12 mm diameter was punched, pressed at 2000 atm, and then placed on the bottom of the cell. First, a Celgard separator was placed on the catholyte tape, then a glass fiber separator was placed on top and filled with 1 M LiPF_6_ in EC:DEC (1:1 vol %) liquid electrolyte. A disk with a 14 mm diameter was punched from Li metal foil and added as the anode to the assembly. CR2032 coin cell casings with aluminum coating on the cathode side were used to avoid parasitic currents that appear, especially in the first cycles. First, two 0.1C formation cycles between 3.0 and 4.17 V vs Li^+^/Li were applied. Finally, the cell was held for 36 h at 3 V vs Li^+^/Li to ensure stable SEI formation on the anode and a high degree of lithiation in the cathode. Then, subsequent 0.1C pulses for 10 min with 2 h relaxation were applied 80 times (*U* limit 4.3 V vs Li^+^/Li). The same pulse relaxation sequence was carried out for discharge (*U* limit 3.0 V vs Li^+^/Li). A final diagnostic cycle similar to the second formation cycle was applied to confirm that no significant changes occurred during the experiment.

### 4.22 Cathode and Anode Composite Preparation

The cathode and anode composite preparation was conducted inside an argon-filled glovebox (LabMaster, MBraun, Germany) with O_2_ and H_2_O levels maintained below 0.1 ppm. The cathode composite was prepared by mixing 69.3 wt % NCM90 or LNO (pristine or coated), 29.7 wt % LPSCl, and 1 wt % VGCF. The anode composite consisted of 20 wt % silicon (pristine or coated), 70 wt % LPSCl, and 10 wt % VGCF. Both cathode and anode mixtures were homogenized by a ball-milling method using a mini-mill PULVERISETTE 23 operated at 30 Hz for 1 h. The resulting composites were employed for the ^Si^SEB^LNO^, ^LiIn^SEB^LNO^, ^LiIn^SEB^NCM90^, and ^LiIn^SEB^Si^ cell assembly.

### 4.23 ^Si^SEB^LNO^ Full-Cell Assembly and Cycling Stability Test

The ^Si^SEB^LNO^ cell configuration consisted of SS|VGCF|Si|LPSCl|LNO|VGCF|SS, where the LNO composite served as the cathode, the silicon composite as the anode, and LPSCl functioned as the anolyte, catholyte, and separator (Figure 3a). When a PEC-LNO cathode composite was paired with a PEC-Si anode composite, the resulting SEB is denoted as ^PEC‑Si^SEB^PEC‑LNO^. Likewise, pairing a pLNO cathode composite with a pSi anode composite yields the SEB denoted as ^pSi^SEB^pLNO^.

Cell assembly was performed using pellet-type asymmetric cells inside an argon-filled glovebox (LabMaster, MBraun, Germany), with O_2_ and H_2_O levels maintained below 0.1 ppm. For the separator, 100 mg of LPSCl was hand-pressed into a 10 mm diameter pellet inside a cylindrical PEEK insulator. Subsequently, 5 mg of the silicon anode composite (1 mg Si, 3.5 mg LPSCl, 0.5 mg VGCF) was hand-pressed onto one side of the separator, and 19 mg of the LNO cathode composite (13.2 mg LNO, 5.6 mg LPSCl, 0.2 mg VGCF) was hand-pressed onto the opposite side. Based on the practical capacity of LNO (≈200 mAh·g^–1^) and the theoretical capacity of silicon (≈3500 mAh·g^–1^), the cathode-specific surface capacity was calculated to be 3.3 mAh·cm^–2^, corresponding to an N/P ratio of 1.3.

After assembly, the ^Si^SEB^LNO^ cell was compressed at 30 kN for 3 min using an Atlas Autotouch automatic press (Specac Ltd., UK), resulting in an 800 μm LPSCl separator, a 65 μm composite cathode, and a 15 μm composite anode. During the cycling stability test, the cell was mounted in an external stainless-steel frame that applied a constant stack pressure of approximately 60 MPa. Cycling stability tests of ^Si^SEB^LNO^ were performed at 25 °C using a MACCOR electrochemical workstation (Maccor Inc., USA). The C-rates were calculated based on a practical capacity of ∼200 mAh·g^–1^ of LNO. The ^Si^SEB^LNO^ cell was initially charged and discharged at C-rates of 0.1C, 0.25C, 0.5C, and 1C within a potential window of 2.0∼3.7 V. Charging was performed up to 4.3 V, followed by a discharge to 2.0 V, with five cycles recorded at each C-rate. Subsequently, long-term cycling was carried out at a consistent rate of 0.1C to evaluate long-term cycling performance.

### 4.24 ^LiIn^SEB^LNO^ and ^LiIn^SEB^NCM90^ Half-Cell Assembly and Electrochemical Test

For half-cell testing of the LNO and NCM90 cathodes, a LiIn alloy was used as the anodic counter electrode. LPSCl served as the catholyte for the LNO and NCM90 cathode composites. For the separator, LPSCl and LPSClBr served as the separators for LNO and NCM90 cathode composites, respectively. Accordingly, the ^LiIn^SEB^LNO^ configuration was SS|LiIn|LPSCl|LNO|VGCF|SS (Figure 4A). When pLNO, PC-LNO, and PEC-LNO were used as the cathode active materials, the SEBs were denoted ^LiIn^SEB^pLNO^, ^LiIn^SEB^PC‑LNO^, and ^LiIn^SEB^PEC‑LNO^, respectively. Similarly, the ^LiIn^SEB^NCM90^ cell configuration was SS|LiIn|LPSClBr|LPSCl|NCM90|VGCF|SS, with the corresponding designations ^LiIn^SEB^pNCM90^ and ^LiIn^SEB^PEC‑NCM90^, when pristine or PEC-coated NCM90 was used. Cell assembly was performed using pellet-type asymmetric cells inside an argon-filled glovebox (LabMaster, MBraun, Garching, Germany) with O_2_ and H_2_O levels maintained below 0.1 ppm.

For ^LiIn^SEB^LNO^, 80 mg of LPSCl was hand-pressed into a 10 mm diameter pellet within a cylindrical PEEK insulator to form the separator. Then, 12 mg of the LNO cathode composite (8.3 mg LNO, 3.6 mg LPSCl, and 0.1 mg VGCF) was hand-pressed onto one side of the separator. For ^LiIn^SEB^NCM90^, 80 mg of LPSClBr was similarly pressed to form the separator, and 20 mg of the NCM90 cathode composite (13.8 mg NCM90, 3.6 mg LPSCl, and 0.1 mg VGCF) was applied onto one side of the separator. The counter electrode consisted of an indium foil (100 μm thick and 9 mm in diameter) placed directly against the separator, followed by a lithium metal foil (125 μm thick and 6 mm in diameter), attached to the current collector and positioned onto the other side of the separator.

After assembly, the ^LiIn^SEB^LNO^ and ^LiIn^SEB^NCM90^ were compressed at 30 kN for 3 min using an automatic Atlas Autotouch press (Specac Ltd., UK), resulting in a separator of 650 μm thickness, along with 40 μm composite LNO and 70 μm composite NCM90 layers. During electrochemical testing, each cell was mounted in an external stainless-steel frame, applying a constant stack pressure of approximately 60 MPa. Rate capability and cycling stability tests of ^LiIn^SEB^LNO^ and ^LiIn^SEB^NCM90^ cells were carried out at 25 °C using a MACCOR electrochemical workstation (Maccor Inc., USA). The potential range was set to 2.0∼3.7 V vs In/LiIn, with charging performed up to 3.7 V, followed by discharge to 2.0 V. The C-rates were calculated based on a practical capacity of ∼200 mAh·g^–1^ for both LNO and NCM90. After each charge–discharge step, a 2 h relaxation period was applied to record the OCP.

For rate capability test, ^LiIn^SEB^LNO^ (2.1 mAh·cm^–2^) was cycled at 0.1C, 0.25C, 0.5C, 1C, and finally again at 0.1C, with five cycles performed at each C-rate. ^LiIn^SEB^pLNO^ was repeated three times. ^LiIn^SEB^PC‑LNO^ was repeated three times. ^LiIn^SEB^PEC‑LNO^ was repeated two times. All the data can be found in Data Availability.

^LiIn^SEB^NCM90^ (3.5 mAh·cm^–2^) was cycled at 0.05C, 0.1C, 0.25C, 0.5C, and finally 1C, also with five cycles at each C-rate. ^LiIn^SEB^pNCM90^ was repeated three times. ^LiIn^SEB^PEC‑NCM90^ was repeated three times. All the data can be found in Data Availability.

For cycling test, ^LiIn^SEB^LNO^ (2.1 mAh·cm^–2^) was cycled at 0.1C. Chronoamperometry and EIS at 3.15 V vs In/LiIn were recorded during the first, second, 50th, and 100th cycles using a fresh cell. ^LiIn^SEB^pLNO^ was repeated two times. ^LiIn^SEB^PEC‑LNO^ was repeated three times. All the data can be found in the Data Availability.

^LiIn^SEB^NCM90^ (3.5 mAh cm^–2^) was cycled at 0.25C immediately following the rate capability sequence described above. ^LiIn^SEB^pNCM90^ was repeated three times. ^LiIn^SEB^PEC‑NCM90^ was repeated three times. All the data can be found in Data Availability.

For the EIS measurements conducted during the cycling stability test, ^LiIn^SEB^LNO^ cells were cycled at 0.1C within a potential window of 2.0 ∼ 3.7 V vs In/LiIn at 25 °C using a VMP-300 (BioLogic, France) electrochemical workstation. Prior to each EIS measurement, the cell was charged at 0.1C to 3.15 V vs In/LiIn. A chronoamperometry step was then applied at this potential until the current decayed to below 2% of the 0.1C. EIS was subsequently recorded over a frequency range of 1 MHz and 100 μHz. The sinusoidal perturbation amplitudes were set to 10 mV (1 mHz∼10 mHz), 5 mV (10 mHz∼1 mHz), and 3 mV (1 mHz∼100 μHz). These amplitude adjustments were made to ensure quasi-linear current responses and to minimize measurement errors, with smaller AC amplitudes used at low frequencies to maintain linearity. After EIS acquisition, the ^LiIn^SEB^LNO^ cell was recharged to 3.7 V vs In/LiIn, allowed to relax for 2 h, and subsequently discharged to 2.0 V vs In/LiIn, followed by another 2 h relaxation period.

### 4.25 ^LiIn^SEB^Si^ Half-Cell Assembly and Electrochemical Testing

For half-cell testing of the silicon anode, a LiIn alloy was employed as the anodic counter electrode. LPSCl functioned as both the anolyte and the separator in combination with the silicon anode composite; thus, the configuration for ^LiIn^SEB^Si^ cells was SS|LiIn|LPSCl|Si|VGCF|SS (Figure 7a). When using pSi, PC-Si, and PEC-Si as anode active materials, the corresponding SEB configurations were denoted as ^LiIn^SEB^pSi^, ^LiIn^SEB^PC‑Si^, and ^LiIn^SEB^PEC‑Si^, respectively.

Cell assembly was carried out using pellet-type asymmetric cells inside an argon-filled glovebox (LabMaster, MBraun, Garching, Germany), with O_2_ and H_2_O levels maintained below 0.1 ppm. For the separator, 80 mg of LPSCl was hand-pressed into a 10 mm diameter pellet inside a cylindrical PEEK insulator. Subsequently, either 5 mg of anode composite (1 mg Si, 3.5 mg LPSCl, 0.5 mg VGCF) or 10 mg of anode composite (2 mg Si, 7 mg LPSCl, 1 mg VGCF) was hand-pressed onto one side of the separator. The counter electrode consisted of an indium foil (100 μm thick, 9 mm diameter) placed directly on the separator side and lithium metal foil (125 μm thick, 6 mm in diameter) attached to the side of the current collector. For cells containing 10 mg of anode composite, two indium pieces and two lithium pieces were required as the counter electrode, doubling the amount used for assemblies with 5 mg composite.

After assembly, the ^LiIn^SEB^Si^ was pressed at 30 kN for 3 min using an Atlas Autotouch automatic press. This process yielded a separator with a thickness of 650 μm and an anode composite thickness of 15 or 30 μm for cells containing 5 mg or 10 mg of composite silicon, respectively. During electrochemical testing, the assembled cell was placed inside an external stainless-steel frame that applied a constant stack pressure of approximately 60 MPa.

^LiIn^SEB^Si^ cell was evaluated for rate capability and cycling stability at 25 °C using a MACCOR electrochemical workstation. The potential window was set between 0.9 and −0.6 V vs In/LiIn, where discharge proceeded to −0.6 V, followed by charge to 0.9 V. C-rates were calculated based on the theoretical specific capacity of silicon (≈3500 mAh·g^–1^). A 2 h relaxation period was applied to SEBs after each charge and discharge step.

For rate capability testing, ^LiIn^SEB^Si^ cells containing 1 mg of silicon (4.5 mAh·cm^–2^) or 2 mg of silicon (9.0 mAh·cm^–2^) were cycled at 0.05C, 0.1C, 0.25C, 0.5C, 1C and finally returned to 0.1C, with five cycles recorded at each C-rate. ^LiIn^SEB^pSi^ (4.5 mAh·cm^–2^) was repeated three times. ^LiIn^SEB^PC‑LNO^ and ^LiIn^SEB^PEC‑LNO^ (4.5 mAh·cm^–2^) were each measured once. ^LiIn^SEB^pSi^ (9 mAh·cm^–2^) was repeated three times. ^LiIn^SEB^PEC‑Si^ (9 mAh·cm^–2^) was repeated two times. All the data can be found in Data Availability.

For cycling stability evaluation, the ^LiIn^SEB^Si^ cell (4.5 mAh·cm^–2^) was cycled at 0.1C from third to 102nd cycle. Chronoamperometry and EIS measurements were conducted at 0.05C and 0 V vs In/LiIn during the first, second, and 103rd cycles. ^LiIn^SEB^PC‑LNO^ and ^LiIn^SEB^PEC‑LNO^ (4.5 mAh·cm^–2^) were each measured twice. All the data can be found in Data Availability.

For the EIS measurements performed during the cycling stability test, ^LiIn^SEB^Si^ cells were operated at 0.05C within a potential window of 0.9 V to −0.6 V vs In/LiIn at 25 °C using a VMP-300 (BioLogic, France) electrochemical workstation. Prior to each EIS measurement, the ^LiIn^SEB^Si^ cell was discharged at 0.05C to 0 V vs In/LiIn, after which chronoamperometry was continuously conducted at this potential until the current decreased to below 2% of the 0.05C.

Following chronoamperometry, EIS spectra were recorded from 1 MHz to 100 μHz. The AC perturbation amplitudes were set to 10 mV (1 MHz∼10 mHz), 5 mV (10 mHz∼1 mHz), and 3 mV (1 mHz∼ 00 μHz). These amplitude adjustments ensured an approximately linear current response and minimized measurement error, as smaller AC signals improve linearity at low frequencies. After EIS acquisition, the ^LiIn^SEB^Si^ cell was discharged to 0.9 V vs In/LiIn, allowed to relax for 2 h, then charged to −0.6 V vs In/LiIn, followed by another 2-h relaxation.

## Supplementary Material



## Data Availability

The data that support the findings of this study are available from the corresponding author upon reasonable request.

## Notes

Additional experimental details, supplementary figures, tables, and characterization data (PDF)

## References

[1] Sen S., Richter F. H. (2023). Typology of Battery Cells – From Liquid to Solid
Electrolytes. Adv. Sci.

[2] Schnell J., Günther T., Knoche T., Vieider C., Köhler L., Just A., Keller M., Passerini S., Reinhart G. (2018). All-solid-state lithium-ion and lithium metal batteries
– paving the way to large-scale production. J. Power Sources.

[3] Tan D. H. S., Meng Y. S., Jang J. (2022). Scaling up high-energy-density sulfidic
solid-state batteries: A lab-to-pilot perspective. Joule.

[4] Yu X., Chen R., Gan L., Li H., Chen L. (2023). Battery Safety:
From Lithium-Ion to Solid-State Batteries. Engineering.

[5] Janek J., Zeier W. G. (2023). Challenges in speeding up solid-state battery development. Nat. Energy.

[6] Janek J., Zeier W. G. (2016). A solid future for battery development. Nat. Energy.

[7] Randau S., Weber D. A., Kötz O., Koerver R., Braun P., Weber A., Ivers-Tiffée E., Adermann T., Kulisch J., Zeier W. G. (2020). Benchmarking the performance of all-solid-state
lithium batteries. Nat. Energy.

[8] Sen S., Trevisanello E., Niemöller E., Shi B.-X., Simon F. J., Richter F. H. (2021). The role of polymers in lithium solid-state batteries
with inorganic solid electrolytes. J. Mater.
Chem. A.

[9] Zhang Q., Cao D., Ma Y., Natan A., Aurora P., Zhu H. (2019). Sulfide-Based
Solid-State Electrolytes: Synthesis, Stability, and Potential for
All-Solid-State Batteries. Adv. Mater.

[10] Banerjee A., Wang X., Fang C., Wu E. A., Meng Y. S. (2020). Interfaces
and Interphases in All-Solid-State Batteries with Inorganic Solid
Electrolytes. Chem. Rev.

[11] Yusim Y., Trevisanello E., Ruess R., Richter F. H., Mayer A., Bresser D., Passerini S., Janek J., Henss A. (2023). Evaluation
and Improvement of the Stability of Poly­(ethylene oxide)-based Solid-state
Batteries with High-Voltage Cathodes. Angew.
Chem. Int. Ed.

[12] Chen R., Li Q., Yu X., Chen L., Li H. (2020). Approaching Practically
Accessible Solid-State Batteries: Stability Issues Related to Solid
Electrolytes and Interfaces. Chem. Rev.

[13] Chakraborty A., Kunnikuruvan S., Kumar S., Markovsky B., Aurbach D., Dixit M., Major D. T. (2020). Layered Cathode
Materials for Lithium-Ion Batteries: Review of Computational Studies
on LiNi1–x–yCoxMnyO2 and LiNi1–x–yCoxAlyO2. Chem. Mater.

[14] Julien C. M., Mauger A. N. (2020). NCM811, and the Route to Ni-Richer Lithium-Ion Batteries. Energies.

[15] Rueß R., Gomboso D., Ulherr M., Trevisanello E., Ma Y., Kondrakov A., Brezesinski T., Janek J. (2023). Single-Crystalline
LiNiO2 as High-Capacity Cathode Active Material for Solid-State Lithium-Ion
Batteries. J. Electrochem. Soc.

[16] Komatsu H., Banerjee S., Holekevi Chandrappa M.
L., Qi J., Radhakrishnan B., Kuwata S., Sakamoto K., Ong S. P. (2022). Interfacial
Stability of Layered LiNixMnyCo1–x–yO2 Cathodes with
Sulfide Solid Electrolytes in All-Solid-State Rechargeable Lithium-Ion
Batteries from First-Principles Calculations. J. Phys. Chem. C.

[17] Tan D. H. S., Wu E. A., Nguyen H., Chen Z., Marple M. A. T., Doux J.-M., Wang X., Yang H., Banerjee A., Meng Y. S. (2019). Elucidating Reversible Electrochemical Redox of Li6PS5Cl
Solid Electrolyte. ACS Energy Lett.

[18] Wang S., Tang M., Zhang Q., Li B., Ohno S., Walther F., Pan R., Xu X., Xin C., Zhang W. (2021). Lithium Argyrodite as Solid Electrolyte and Cathode
Precursor for Solid-State Batteries with Long Cycle Life. Adv. Energy Mater.

[19] Auvergniot J., Cassel A., Ledeuil J.-B., Viallet V., Seznec V., Dedryvère R. (2017). Interface Stability of Argyrodite Li6PS5Cl toward LiCoO2,
LiNi1/3Co1/3Mn1/3O2, and LiMn2O4 in Bulk All-Solid-State Batteries. Chem. Mater.

[20] Dewald G. F., Ohno S., Kraft M. A., Koerver R., Till P., Vargas-Barbosa N. M., Janek J., Zeier W. G. (2019). Experimental Assessment
of the Practical Oxidative Stability of Lithium Thiophosphate Solid
Electrolytes. Chem. Mater.

[21] Randau S., Walther F., Neumann A., Schneider Y., Negi R. S., Mogwitz B., Sann J., Becker-Steinberger K., Danner T., Hein S. (2021). On the Additive Microstructure
in Composite Cathodes and Alumina-Coated Carbon Microwires for Improved
All-Solid-State Batteries. Chem. Mater.

[22] Jung S.-K., Gwon H., Lee S.-S., Kim H., Lee J. C., Chung J. G., Park S. Y., Aihara Y., Im D. (2019). Understanding
the effects of chemical reactions at the cathode–electrolyte
interface in sulfide based all-solid-state batteries. J. Mater. Chem. A.

[23] Bartsch T., Strauss F., Hatsukade T., Schiele A., Kim A. Y., Hartmann P., Janek J., Brezesinski T. (2018). Gas Evolution
in All-Solid-State Battery Cells. ACS Energy
Lett.

[24] Zuo T.-T., Rueß R., Pan R., Walther F., Rohnke M., Hori S., Kanno R., Schröder D., Janek J. (2021). A mechanistic investigation of the Li10GeP2S12|LiNi1-x-yCoxMnyO2
interface stability in all-solid-state lithium batteries. Nat. Commun.

[25] Conforto G., Ruess R., Schröder D., Trevisanello E., Fantin R., Richter F. H., Janek J. (2021). Editors’ Choice—Quantification
of the Impact of Chemo-Mechanical Degradation on the Performance and
Cycling Stability of NCM-Based Cathodes in Solid-State Li-Ion Batteries. J. Electrochem. Soc.

[26] Yoon C. S., Jun D.-W., Myung S.-T., Sun Y.-K. (2017). Structural Stability
of LiNiO2 Cycled above 4.2 V. ACS Energy Lett.

[27] Kim M., Ahn H., Choi J., Kim W. B. (2023). A Rational Design of Silicon-Based
Anode for All-Solid-State Lithium-Ion Batteries: A Review. Energy Technol.

[28] Franco
Gonzalez A., Yang N.-H., Liu R.-S. (2017). Silicon Anode Design
for Lithium-Ion Batteries: Progress and Perspectives. J. Phys. Chem. C.

[29] Loveridge M. J., Lain M. J., Johnson I. D., Roberts A., Beattie S. D., Dashwood R., Darr J. A., Bhagat R. (2016). Towards High Capacity
Li-ion Batteries Based on Silicon-Graphene Composite Anodes and Sub-micron
V-doped LiFePO4 Cathodes. Sci. Rep.

[30] Eshetu G. G., Zhang H., Judez X., Adenusi H., Armand M., Passerini S., Figgemeier E. (2021). Production of high-energy Li-ion
batteries comprising silicon-containing anodes and insertion-type
cathodes. Nat. Commun.

[31] Xiao Z., Wang C., Song L., Zheng Y., Long T. (2022). Research progress
of nano-silicon-based materials and silicon-carbon composite anode
materials for lithium-ion batteries. J. Solid
State Electrochem.

[32] Huo H., Jiang M., Bai Y., Ahmed S., Volz K., Hartmann H., Henss A., Singh C. V., Raabe D., Janek J. (2024). Chemo-mechanical failure mechanisms of the silicon anode in solid-state
batteries. Nat. Mater.

[33] Cao D., Sun X., Li Y., Anderson A., Lu W., Zhu H. (2022). Long-Cycling
Sulfide-Based All-Solid-State Batteries Enabled by Electrochemo-Mechanically
Stable Electrodes. Adv. Mater.

[34] Jayasubramaniyan S., Kim S., Ko M., Sung J. (2024). Recent Progress of In-Depth Analysis
Techniques for Si Anodes in Sulfide-Based All-Solid-State Batteries:
A Concise Overview and Future Perspective. ChemElectroChem.

[35] Yan J., Huang H., Tong J., Li W., Liu X., Zhang H., Huang H., Zhou W. (2022). Recent progress on
the modification of high nickel content NCM: Coating, doping, and
single crystallization. Interdiscip. Mater.

[36] Duan Y., Chen S.-P., Zhang L., Guo L., Shi F.-N. (2024). Review
on Oxygen Release Mechanism and Modification Strategy of Nickel-Rich
NCM Cathode Materials for Lithium-Ion Batteries: Recent Advances and
Future Directions. Energy Fuels.

[37] Zhan X., Li M., Li S., Pang X., Mao F., Wang H., Sun Z., Han X., Jiang B., He Y.-B. (2023). Challenges
and opportunities towards silicon-based all-solid-state batteries. Energy Storage Mater.

[38] Song W., Chae O. B. (2024). Surface-Coating Strategies of Si-Negative Electrode
Materials in Lithium-Ion Batteries. Batteries.

[39] Nakamura T., Amezawa K., Kulisch J., Zeier W. G., Janek J. (2019). Guidelines
for All-Solid-State Battery Design and Electrode Buffer Layers Based
on Chemical Potential Profile Calculation. ACS
Appl. Mater. Interfaces.

[40] Culver S. P., Koerver R., Zeier W. G., Janek J. (2019). On the Functionality
of Coatings for Cathode Active Materials in Thiophosphate-Based All-Solid-State
Batteries. Adv. Energy Mater.

[41] Amin R., Nisar U., Rahman M. M., Dixit M., Abouimrane A., Belharouak I. (2024). Prospects of polymer coatings for all solid-state and
emerging Li-ion batteries. J. Mater. Chem. A.

[42] Wilson M. K., Augustin C., Abhilash A., Jinisha B., Antony A., Jayaraj M. K., Jayalekshmi S. (2024). Solid electrolyte membranes with
Al2O3 nanofiller for fully solid-state Li-ion cells. Polym. Bull.

[43] Shi B.-X., Yusim Y., Sen S., Demuth T., Ruess R., Volz K., Henss A., Richter F. H. (2023). Mitigating Contact
Loss in Li6PS5Cl-Based Solid-State Batteries Using a Thin Cationic
Polymer Coating on NCM. Adv. Energy Mater.

[44] Shi B.-X., Weber F., Yusim Y., Demuth T., Vettori K., Münchinger A., Titvinidze G., Volz K., Henss A., Berger R. (2025). Lithiated polymer coating for interface stabilization
in Li6PS5Cl-based solid-state batteries with high-nickel NCM. J. Mater. Chem. A.

[45] Meka V. S., Sing M. K. G., Pichika M. R., Nali S. R., Kolapalli V. R. M., Kesharwani P. (2017). A comprehensive review on polyelectrolyte complexes. Drug Discovery Today.

[46] van
Lange S. G. M., Te Brake D. W., Portale G., Palanisamy A., Sprakel J., van der Gucht J. (2024). Moderated ionic bonding for water-free
recyclable polyelectrolyte complex materials. Sci. Adv.

[47] Lee M., Perry S. L., Hayward R. C. (2021). Complex Coacervation of Polymerized
Ionic Liquids in Non-aqueous Solvents. ACS Polym.
Au.

[48] Meng S., Liu Y., Yeo J., Ting J. M., Tirrell M. V. (2020). Effect of mixed
solvents on polyelectrolyte complexes with salt. Colloid Polym. Sci.

[49] Jirgensons B., Straumanis M. E., Hutchison A. W. (1955). A Short Textbook of Colloid Chemistry. J. Electrochem. Soc.

[50] Wang Q., Schlenoff J. B. (2014). The Polyelectrolyte Complex/Coacervate Continuum. Macromolecules.

[51] Balding P., Borrelli R., Volkovinsky R., Russo P. S. (2022). Physical Properties
of Sodium Poly­(styrene sulfonate): Comparison to Incompletely Sulfonated
Polystyrene. Macromolecules.

[52] Brinkkötter M., Lozinskaya E. I., Ponkratov D. O., Vlasov P. S., Rosenwinkel M. P., Malyshkina I. A., Vygodskii Y., Shaplov A. S., Schönhoff M. (2017). Influence
of anion structure on ion dynamics in polymer gel electrolytes composed
of poly­(ionic liquid), ionic liquid and Li salt. Electrochim. Acta.

[53] Manoj
Lalwani S., Eneh C. I., Lutkenhaus J. L. (2020). Emerging
trends in the dynamics of polyelectrolyte complexes. Phys. Chem. Chem. Phys.

[54] Alaa
Eddine M., Nosov D. R., Lepre L. F., Serghei A., Schmidt D. F., Montarnal D., Shaplov A. S., Drockenmuller E. (2024). Dynamic Ion
Gels from the Complex Coacervation of Oppositely Charged Poly­(ionic
liquid)­s. ACS Macro Lett.

[55] Lozinskaya E. I., Ponkratov D. O., Malyshkina I. A., Grysan P., Lingua G., Gerbaldi C., Shaplov A. S., Vygodskii Y. S. (2022). Self-assembly
of Li single-ion-conducting block copolymers for improved conductivity
and viscoelastic properties. Electrochim. Acta.

[56] Xue Z., He D., Xie X. (2015). Poly­(ethylene oxide)-based electrolytes for lithium-ion
batteries. J. Mater. Chem. A.

[57] Fares H. M., Schlenoff J. B. (2017). Diffusion of Sites versus Polymers in Polyelectrolyte
Complexes and Multilayers. J. Am. Chem. Soc.

[58] Luo S., Wang Z., Li X., Liu X., Wang H., Ma W., Zhang L., Zhu L., Zhang X. (2021). Growth of lithium-indium
dendrites in all-solid-state lithium-based batteries with sulfide
electrolytes. Nat. Commun.

[59] Hong C., Leng Q., Zhu J., Zheng S., He H., Li Y., Liu R., Wan J., Yang Y. (2020). Revealing the correlation
between structural evolution and Li+ diffusion kinetics of nickel-rich
cathode materials in Li-ion batteries. J. Mater.
Chem. A.

[60] Koerver R., Aygün I., Leichtweiß T., Dietrich C., Zhang W., Binder J. O., Hartmann P., Zeier W. G., Janek J. (2017). Capacity Fade
in Solid-State Batteries: Interphase Formation and Chemomechanical
Processes in Nickel-Rich Layered Oxide Cathodes and Lithium Thiophosphate
Solid Electrolytes. Chem. Mater.

[61] Mock M., Bianchini M., Fauth F., Albe K., Sicolo S. (2021). Atomistic
understanding of the LiNiO2–NiO2 phase diagram from experimentally
guided lattice models. J. Mater. Chem. A.

[62] Levi, M. D. ; Aurbach, D. Potentiostatic and Galvanostatic Intermittent Titration Techniques. In Characterization of Materials. Wiley: 2012 pp. 1–21.

[63] Trevisanello E., Ruess R., Conforto G., Richter F. H., Janek J. (2021). Polycrystalline
and Single Crystalline NCM Cathode Materials—Quantifying Particle
Cracking, Active Surface Area, and Lithium Diffusion. Adv. Energy Mater.

[64] Waag W., Käbitz S., Sauer D. U. (2013). Experimental investigation of the
lithium-ion battery impedance characteristic at various conditions
and aging states and its influence on the application. Appl. Energy.

[65] Li S., Lin J., Schaller M., Indris S., Zhang X., Brezesinski T., Nan C.-W., Wang S., Strauss F. (2023). High-Entropy Lithium
Argyrodite Solid Electrolytes Enabling Stable All-Solid-State Batteries. Angew. Chem. Int. Ed.

[66] Ruess R., Schweidler S., Hemmelmann H., Conforto G., Bielefeld A., Weber D. A., Sann J., Elm M. T., Janek J. (2020). Influence
of NCM Particle Cracking on Kinetics of Lithium-Ion Batteries with
Liquid or Solid Electrolyte. J. Electrochem.
Soc.

[67] Touboul M., Sephar N., Quarton M. (1990). Electrical conductivity and phase
diagram of the system Li2SO4Li3PO4. Solid State
Ionics.

[68] Homma K., Liu Y., Sumita M., Tamura R., Fushimi N., Iwata J., Tsuda K., Kaneta C. (2020). Optimization of a Heterogeneous Ternary
Li3PO4–Li3BO3–Li2SO4Mixture for Li-Ion Conductivity
by Machine Learning. J. Phys. Chem. C.

[69] Weiss M., Simon F. J., Busche M. R., Nakamura T., Schröder D., Richter F. H., Janek J. (2020). From Liquid- to Solid-State Batteries:
Ion Transfer Kinetics of Heteroionic Interfaces. Electrochem. Energy Rev.

[70] Zuo T.-T., Walther F., Teo J. H., Rueß R., Wang Y., Rohnke M., Schröder D., Nazar L. F., Janek J. (2023). Impact of the Chlorination of Lithium
Argyrodites on the Electrolyte/Cathode Interface in Solid-State Batteries. Angew. Chem. Int. Ed.

[71] Hori S., Kanno R., Sun X., Song S., Hirayama M., Hauck B., Dippon M., Dierickx S., Ivers-Tiffée E. (2023). Understanding
the impedance spectra of all-solid-state lithium battery cells with
sulfide superionic conductors. J. Power Sources.

[72] Wei C., Liu C., Xiao Y., Wu Z., Luo Q., Jiang Z., Wang Z., Zhang L., Cheng S., Yu C. (2024). SnF2-Induced
Multifunctional Interface-Stabilized Li5.5PS4.5Cl1.5-Based All-Solid-State
Lithium Metal Batteries. Adv. Funct. Mater.

[73] Fukunishi G., Tabuchi M., Ikezawa A., Okajima T., Kitamura F., Suzuki K., Hirayama M., Kanno R., Arai H. (2023). AC impedance
analysis of NCM523 composite electrodes in all-solid-state three electrode
cells and their degradation behavior. J. Power
Sources.

[74] Truong T. K., Whang G., Huang J., Sandoval S. E., Zeier W. G. (2025). Probing
solid-state battery aging: evaluating calendar vs. cycle aging protocols
via time-resolved electrochemical impedance spectroscopy. J. Mater. Chem. A.

[75] Minnmann P., Quillman L., Burkhardt S., Richter F. H., Janek J. (2021). Editors’
Choice—Quantifying the Impact of Charge Transport Bottlenecks
in Composite Cathodes of All-Solid-State Batteries. J. Electrochem. Soc.

[76] Negi R. S., Yusim Y., Pan R., Ahmed S., Volz K., Takata R., Schmidt F., Henss A., Elm M. T. (2022). A Dry-Processed
Al2O3/LiAlO2 Coating for Stabilizing the Cathode/Electrolyte Interface
in High-Ni NCM-Based All-Solid-State Batteries. Adv. Mater. Interfaces.

[77] Aspinall J., Chart Y., Guo H., Shrestha P., Burton M., Pasta M. (2024). Effect of Microstructure on the Cycling Behavior of Li–In
Alloy Anodes for Solid-State Batteries. ACS
Energy Lett.

[78] Walther F., Koerver R., Fuchs T., Ohno S., Sann J., Rohnke M., Zeier W. G., Janek J. (2019). Visualization of the
Interfacial Decomposition of Composite Cathodes in Argyrodite-Based
All-Solid-State Batteries Using Time-of-Flight Secondary-Ion Mass
Spectrometry. Chem. Mater.

[79] Fantauzzi M., Elsener B., Atzei D., Rigoldi A., Rossi A. (2015). Exploiting
XPS for the identification of sulfides and polysulfides. RSC Adv.

[80] Sigar U., Weintraut T., Benz S. L., Engün S., Kremer S., Henss A., Richter F. H. (2025). Low Resistance Interphase
Formation at the PEO-LiTFSI|LGPS Interface in Lithium Solid-State
Batteries. Adv. Mater. Interfaces.

[81] Wenzel S., Sedlmaier S. J., Dietrich C., Zeier W. G., Janek J. (2018). Interfacial
reactivity and interphase growth of argyrodite solid electrolytes
at lithium metal electrodes. Solid State Ionics.

[82] Fujita Y., Motohashi K., Sakuda A., Hayashi A. (2024). Electrochemical Redox
Potential of Li2SO4 Investigated Using the Appropriate All-Solid-State
Cell Configuration. J. Phys. Chem. C.

[83] Walther F., Randau S., Schneider Y., Sann J., Rohnke M., Richter F. H., Zeier W. G., Janek J. (2020). Influence of Carbon
Additives on the Decomposition Pathways in Cathodes of Lithium Thiophosphate-Based
All-Solid-State Batteries. Chem. Mater.

[84] Vickerman, J. C. Surface Analysis – The Principal Techniques In Molecular Surface Mass Spectrometry By SIMS. Wiley: 2009, pp. 113, 10.1002/9780470721582.ch4.

[85] Walther F., Strauss F., Wu X., Mogwitz B., Hertle J., Sann J., Rohnke M., Brezesinski T., Janek J. (2021). The Working Principle of a Li2CO3/LiNbO3 Coating on NCM for Thiophosphate-Based
All-Solid-State Batteries. Chem. Mater.

[86] Lombardo T., Walther F., Kern C., Moryson Y., Weintraut T., Henss A., Rohnke M. (2023). ToF-SIMS in battery research: Advantages,
limitations, and best practices. J. Vacuum Sci.
Technol. A.

[87] Yusim Y., Moryson Y., Seipp K., Sann J., Henss A. (2024). Challenges
in XPS Analysis of PEO-LiTFSI-Based Solid Electrolytes: How to Overcome
X-Ray-Induced Photodecomposition. Batteries
Supercaps.

[88] Jing S., Wang K., Li S., Lu Y., Chen Y., Zhang K., Li F., Yin S., Zhang Z., Liu F. (2025). An all-in-one approach for sulfide solid electrolyte with bidirectional
stabilization shells enabling 4.6 V all-solid-state lithium batteries. Energy Storage Mater.

[89] He D., Yuan M., Hu B., Zou C., Zhao X., Zhao C., Wang J., Ding H. (2024). Effect of Pressure
on Si-Based Anode Performance in All-Solid-State Batteries. J. Phys.: Conf. Ser..

[90] Rehnlund D., Lindgren F., Böhme S., Nordh T., Zou Y., Pettersson J., Bexell U., Boman M., Edström K., Nyholm L. (2017). Lithium trapping in alloy forming electrodes and current
collectors for lithium based batteries. Energy
Environ. Sci.

[91] Graf M., Berg C., Bernhard R., Haufe S., Pfeiffer J., Gasteiger H. A. (2022). Effect and Progress of the Amorphization Process for
Microscale Silicon Particles under Partial Lithiation as Active Material
in Lithium-Ion Batteries. J. Electrochem. Soc.

[92] Casino S., Beuse T., Küpers V., Börner M., Gallasch T., Winter M., Niehoff P. (2021). Quantification of aging
mechanisms of carbon-coated and uncoated silicon thin film anodes
in lithium metal and lithium ion cells. J. Energy
Storage.

[93] Ulvestad A., Andersen H. F., Jensen I. J. T., Mongstad T. T., Mæhlen J. P., Prytz Ø., Kirkengen M. (2018). Substoichiometric Silicon Nitride
– An Anode Material for Li-ion Batteries Promising High Stability
and High Capacity. Sci. Rep.

[94] Ulvestad A., Andersen H. F., Mæhlen J. P., Prytz Ø., Kirkengen M. (2017). Long-term
Cyclability of Substoichiometric Silicon Nitride Thin Film Anodes
for Li-ion Batteries. Sci. Rep.

[95] Köbbing L., Latz A., Horstmann B. (2024). Voltage Hysteresis of Silicon Nanoparticles:
Chemo-Mechanical Particle-SEI Model. Adv. Funct.
Mater.

[96] Hertzberg B., Benson J., Yushin G. (2011). Ex-situ depth-sensing indentation
measurements of electrochemically produced Si–Li alloy films. Electrochem. Commun.

[97] Kitaba L. T., Nikodimos Y., Merso S. K., Taklu B. W., Serbessa G. G., Dilebo W. B., Yeh T.-I., Iskandar J. A., Valencia F., Chang C.-Y. (2025). Overcoming Chemo-Mechanical Instability at
Silicon-Solid Electrolyte Interfaces in Solid-State Batteries. ACS Appl. Mater. Interfaces.

[98] Huo H., Jiang M., Mogwitz B., Sann J., Yusim Y., Zuo T.-T., Moryson Y., Minnmann P., Richter F. H., Veer Singh C. (2023). Interface Design Enabling Stable Polymer/Thiophosphate
Electrolyte Separators for Dendrite-Free Lithium Metal Batteries. Angew. Chem. Int. Ed.

[99] Lee J. M., Park Y. S., Moon J.-W., Hwang H. (2021). Ionic and Electronic
Conductivities of Lithium Argyrodite Li6PS5Cl Electrolytes Prepared
via Wet Milling and Post-Annealing. Front. Chem..

[100] Karger L., Nunes B. N., Yusim Y., Mazilkin A., Zhang R., Zhao W., Henss A., Kondrakov A., Janek J., Brezesinski T. (2024). Protective Nanosheet Coatings for
Thiophosphate-Based All-Solid-State Batteries. Adv. Mater. Interfaces.

[101] Porcarelli L., Shaplov A. S., Salsamendi M., Nair J. R., Vygodskii Y. S., Mecerreyes D., Gerbaldi C. (2016). Single-Ion Block Copoly­(ionic liquid)­s as Electrolytes
for All-Solid State Lithium Batteries. ACS Appl.
Mater. Interfaces.

[102] Vasilevskaya V. V., Lozinskaya E. I., Lazutin A. A., Buglakov A. I., Grysan P., Nosov D. R., Schmidt D. F., Shaplov A. S. (2025). From Computer
Simulation to Synthesis of Highly Lithium-Ion Conductive Block Copolymers
with Bicontinuous Morphology. ACS Appl. Polym.
Mater.

