# Supplementary material for: Stabilizing Solid-State Battery Interfaces with a Polyelectrolyte Complex Nanocoating
Source: ACS Nano. 2026 Aug 3;20(32):22674–95. doi: 10.1021/acsnano.6c05725 (PMC13492473; doi:10.1021/acsnano.6c05725)
Supplement: Supplementary file 1 [file nn6c05725_si_001.pdf]

Supporting information

Stabilizing Solid-State Battery Interfaces with a Polyelectrolyte Complex Nanocoating

Bing-Xuan Shi,[a] Daniil R. Nosov,[b] Timo Weintraut,[c] Mariapaola Staropoli,[b] Thomas Demuth,[d,e] Felix Schnaubelt,[a] Sebastian Leonard Benz,[a] Kilian Vettori,[a] Xiuxia Zuo,[f] Jingui Yang,[g] Florian Strauss,[g] Kerstin Volz,[d,e] Anja Henss,[c] Alexander S. Shaplov,[b] Felix H. Richter*[a]

[a] Institute of Physical Chemistry & Center for Materials Research (LaMa), Justus-Liebig-University Giessen, Heinrich-Buff-Ring 17, 35392 Giessen, Germany.

[b] Luxembourg Institute of Science and Technology, Functional Polymeric and Particulate Materials Unit, 5 Avenue des Hauts-Fourneaux, 4362 Esch-sur-Alzette, Luxembourg.

[c] Institute of Experimental Physics 1 & Center for Materials Research (LaMa), Justus-Liebig-University Giessen, Heinrich-Buff-Ring 17, 35392 Giessen, Germany.

[d] Philipps-Universität Marburg, mar.quest | Marburg Center for Quantum Materials and Sustainable Technologies, 35032 Marburg, Germany.

[e] Philipps-Universität Marburg, Department of Physics, Hans-Meerwein Straße 6, 35032 Marburg, Germany.

[f] School of New Energy, Ningbo University of Technology No. 201, Fenghua Road, Jiangbei District, Ningbo, Zhejiang 315211, P. R. China; Ningbo Institute of Materials Technology and Engineering, Chinese Academy of Sciences, 1219 Zhongguan West Road, Zhenhai District, Ningbo, Zhejiang 315201, P. R. China.

[g] Institute of Nanotechnology (INT), Karlsruhe Institute of Technology (KIT), Kaiserstr. 12, 76131 Karlsruhe, Germany.

*Corresponding author: Felix.H.Richter@phys.chemie.uni-giessen.de

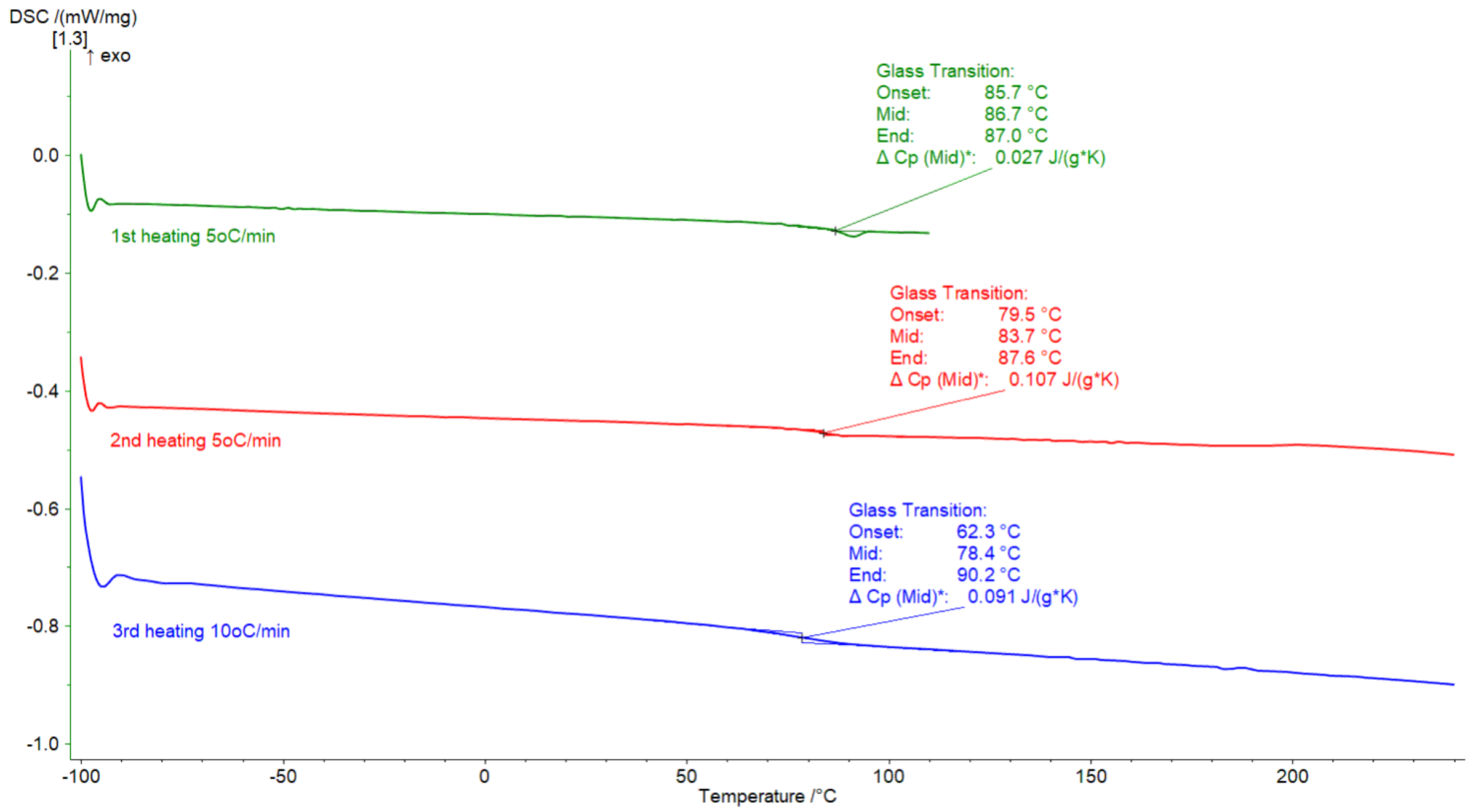

Figure S1. DSC plots (exo/up) of PC at different heating rates.

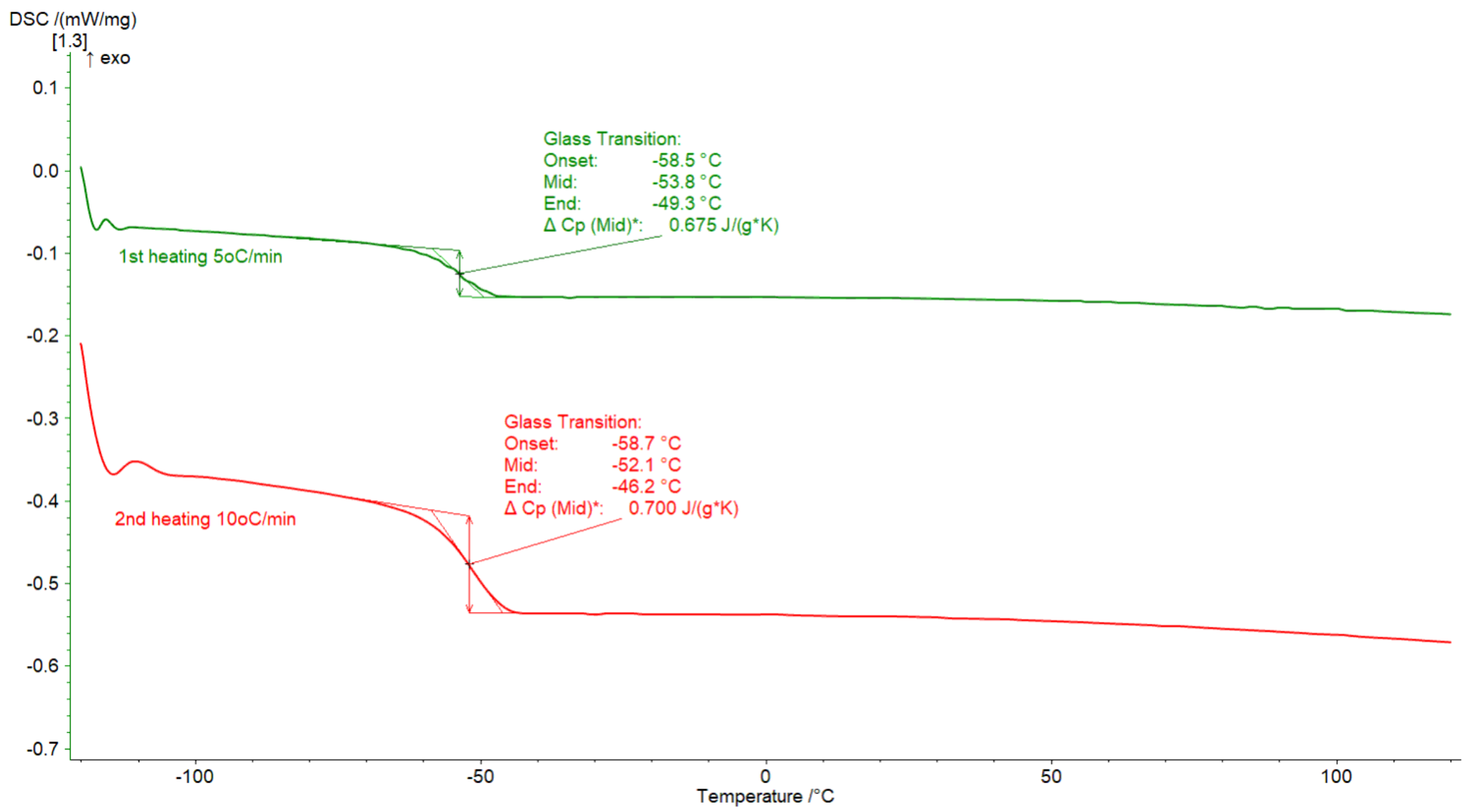

Figure S2. DSC plots (exo/up) of PA at different heating rates.

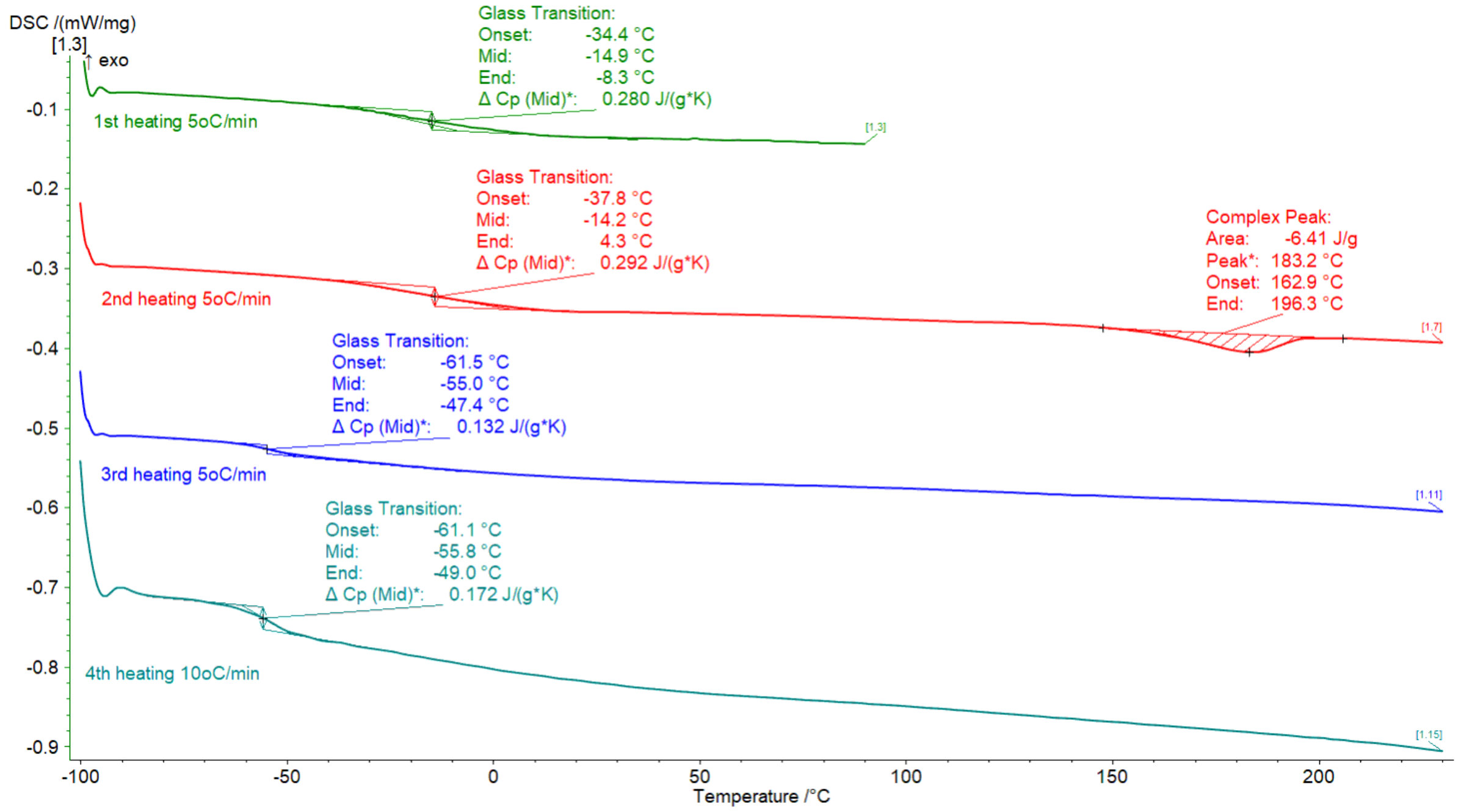

Figure S3. DSC plots (exo/up) of PEC at different heating rates.

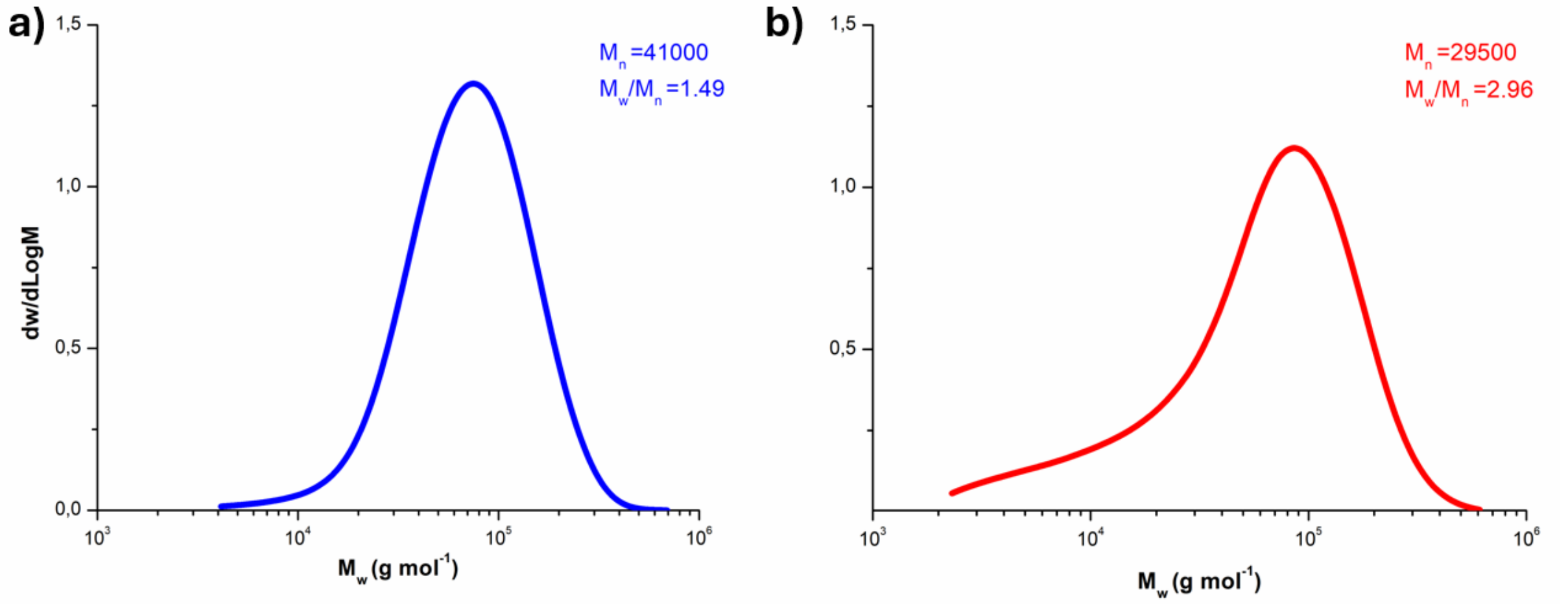

Figure S4. GPC (SEC) traces of (a) PA and (b) PC at 50 °C, 0.1 M LiTFSI in DMF, and calibration with PMMA standards.

Table S1. Selected properties of PC, PA, and PEC.

	M_n / g·mol^{-1}	M_w/M_n	T_g^{a} / °C	T_m^{a} / °C	T_{onset}^{b} / °C	G' at 1 rad·s^{-1} and 25 °C^c / kPa	σ_{DC} at 25°C^d / S·cm^{-1}
**PC**	29500	2.96	84	-	290	56.8	2.1×10^{-9}
**PA**	41000	1.49	−54	-	150	4.3×10^{-3}	8.2×10^{-7}
**PEC**	-	-	−14	183	230	109.9	8.8×10^{-7}

[a] Glass transition temperature (T_g) and melting point (T_m) by differential scanning calorimetry, DSC (5 °C·min^{-1}).

[b] Onset loss temperature (T_{onset}) by thermogravimetric analysis, TGA (5 °C·min^{-1}, in air).

[c] Storage modulus (G') determined by rheology.

[d] Ionic conductivity by electrochemical impedance spectroscopy (EIS) for PA and PEC, by broad dielectric spectroscopy (BDS) for PC.

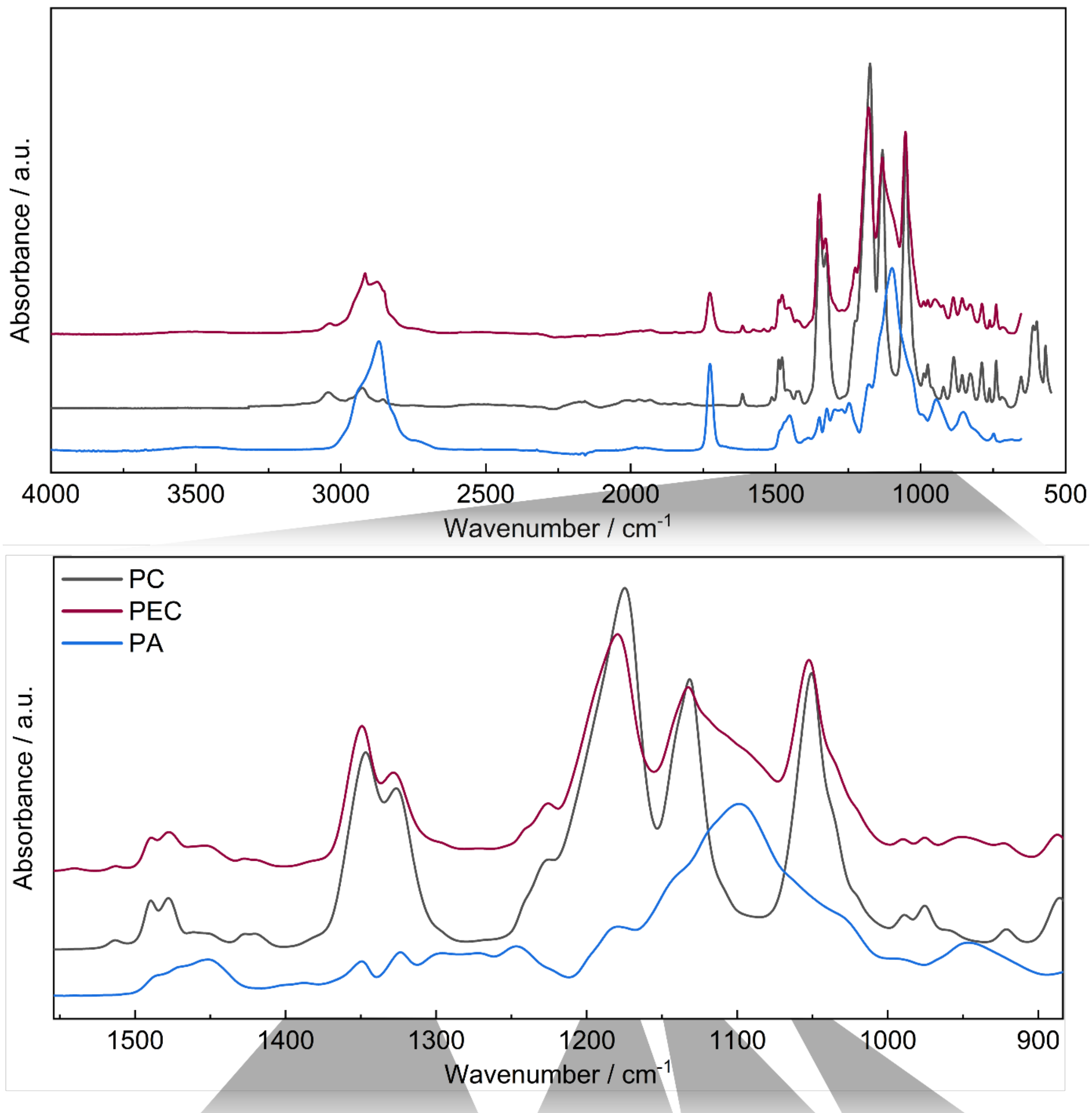

	TFSI⁻ Wavenumber / cm⁻¹				
PC	1349	1328	1179	1133	1052
PEC	1346	1327	1174	1132	1050
PA	1349	1324	1179		

Figure S5. FTIR reveals slight wavelength shifts in the TFSI⁻ group. The top figure displays the full spectrum from 4000 to 500 cm^{-1}, while the middle figure highlights the IR range specific to the TFSI⁻ group (1550 to 880 cm^{-1}). The bottom table summarizes the shift values for the TFSI⁻ group.

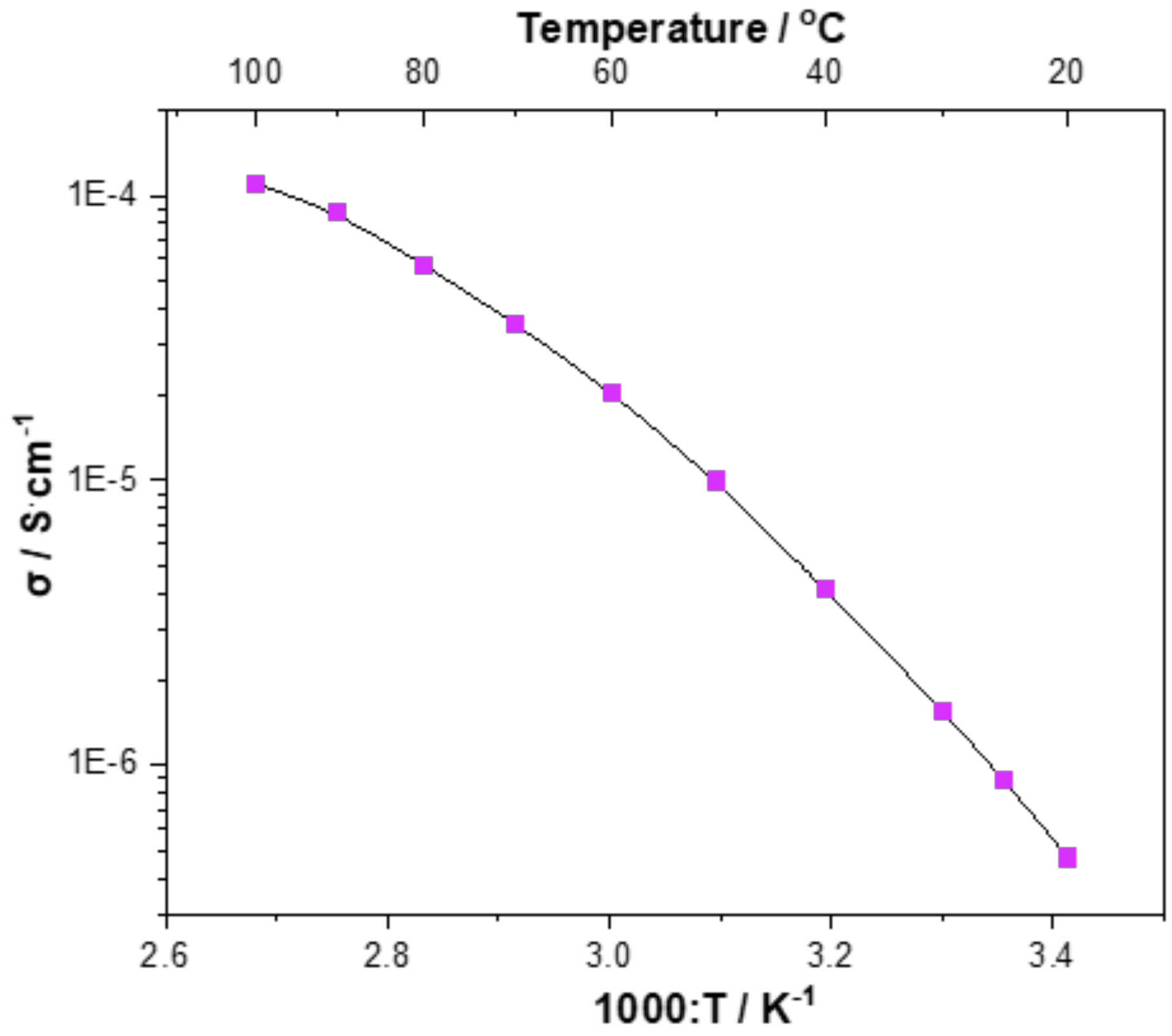

Figure S6. Reciprocal temperature dependence of ionic conductivity σ_{DC}, measured by EIS for PEC film (cell configuration: SS|PEC|SS).

The ohmic resistance (R_{Ω}) of the sample, obtained by linear fit from the Nyquist plot at the low frequency end of the semicircle, was used to calculate the ionic conductivity using the following equation:

$$\sigma = \sigma_{AC} = \frac{l}{S}\frac{1}{R_{\Omega}} \quad \text{(eq. S1)}$$

where *l* is the sample thickness and *S* is the electrode area.

The determined values of the AC conductivity for PC, PA and PEC are presented in Table S1.

The inverse temperature dependence of ionic conductivity follows a typical Vogel-Fulcher-Tammann (VFT) relationship for PEC (**Figure S4**). The experimental results were thus fitted with the VFT equation S2:

$$\sigma_{DC} = \sigma_{\infty} \times exp(-\frac{B}{(T-T_{VFT})}) \quad \text{(eq S2)}$$

where σ_{∞} is the ionic conductivity in the limit of high temperatures, B is the fitting parameter related to the activation energy of the ionic conduction, and T_{VFT} is the Vogel temperature. The obtained parameters for PEC are listed in **Table S2**.

Table S2. Parameters obtained by VFT fits of the EIS data for PEC.

	σ_{∞} / S cm^{-1}	B / K	T_{VFT} / K	$T_g - T_{VFT}$ / K	σ_{DC} at 25 °C / S cm^{-1}	E_a[#] / kJ mol^{-1}
**PEC**	0.0070	902	195	64	8.8×10^{-7}	46.9

[#] E_a: activation energy was determined from the slope of the best linear fit to the VFT data.

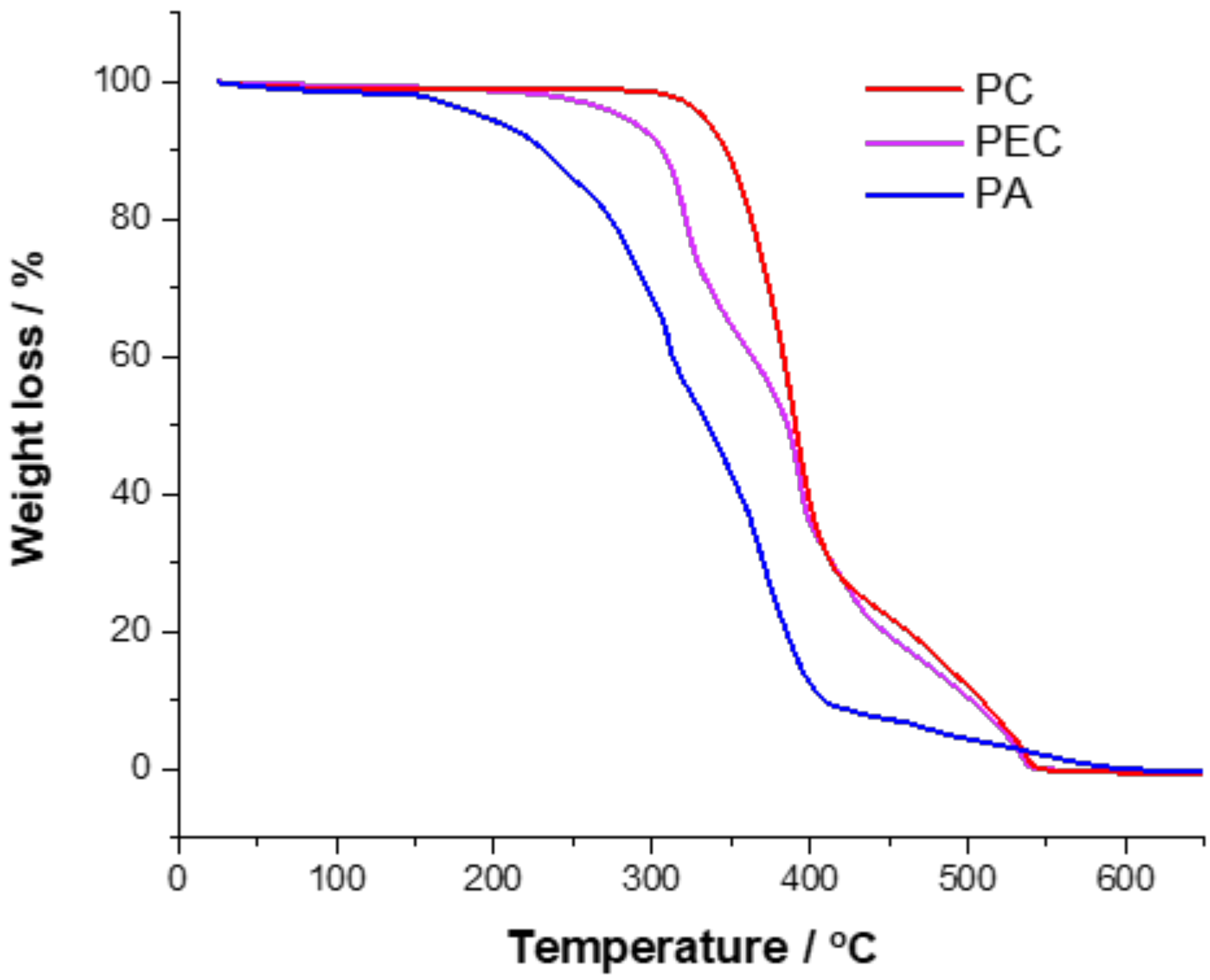

Figure S7. TGA plots of PA, PC, and PEC.

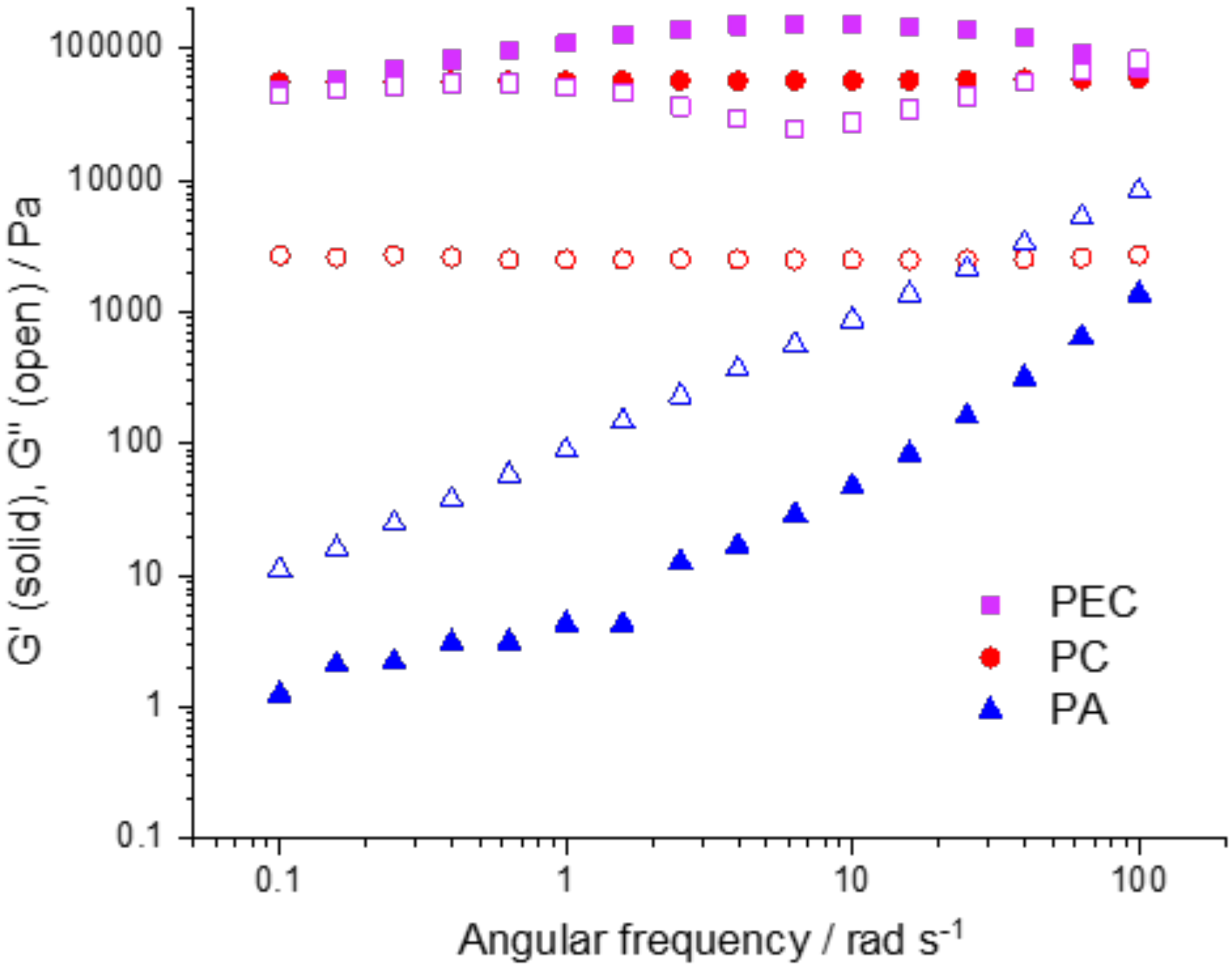

Figure S8. Frequency dependence of the storage modulus (solid symbols) and the loss modulus (open symbols) obtained at 25 °C for PA, PC, and PEC.

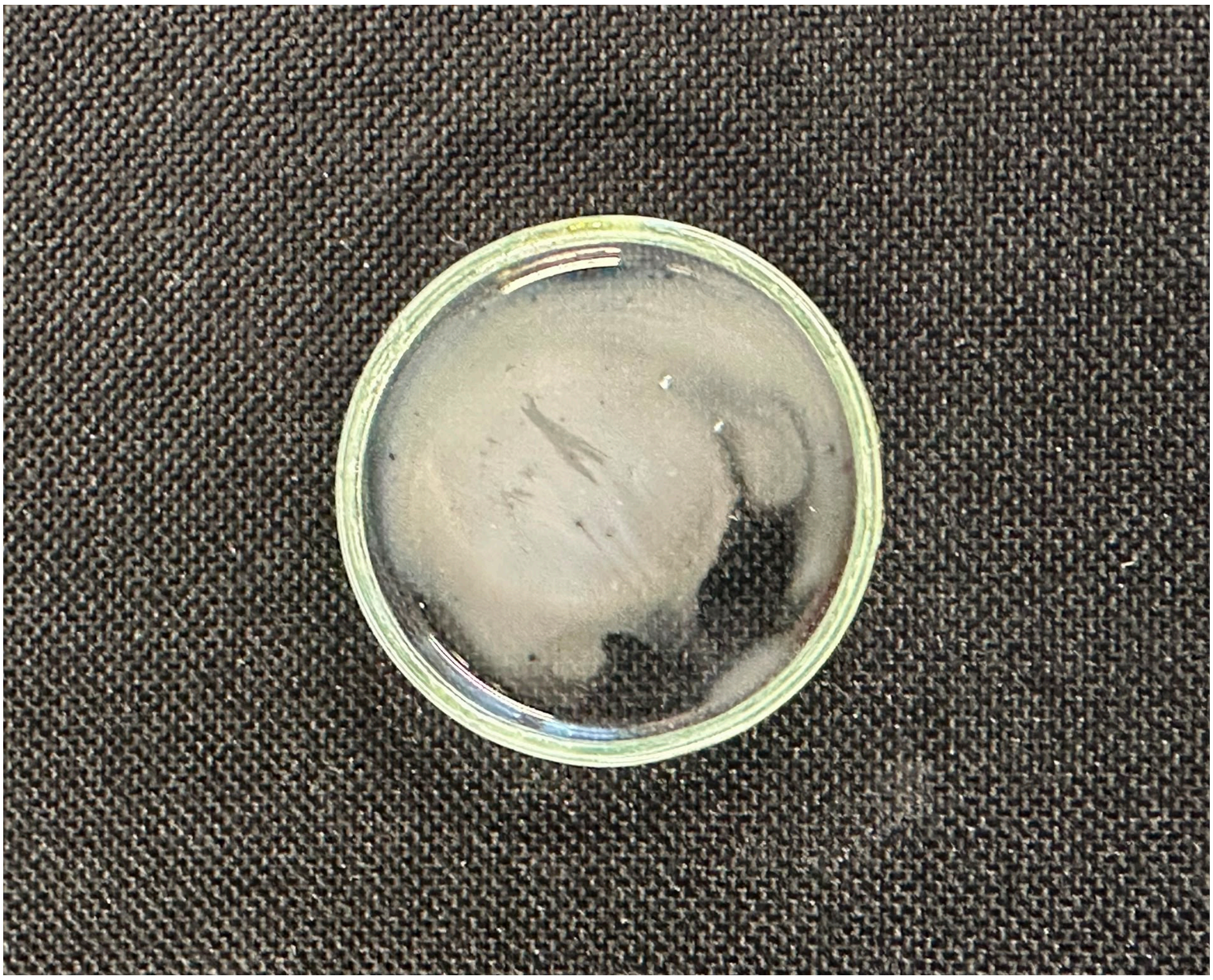

Figure S9. Visual appearance of the PC film containing LiTFSI (PC:LiTFSI = 15:1 by weight, corresponding to 9.4:1 molar ratio).

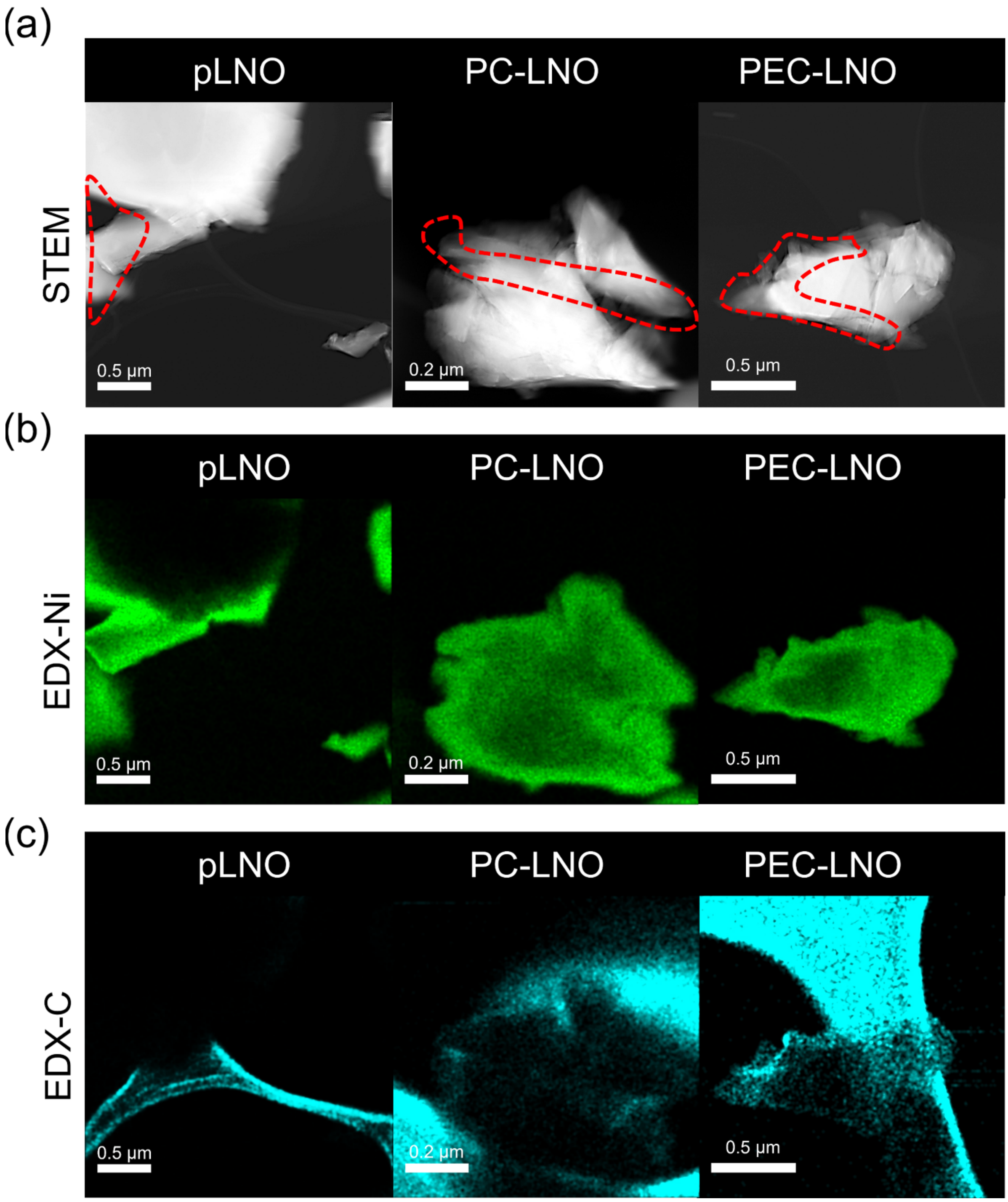

Figure S10. (a) STEM images of pLNO, PC-LNO, and PEC-LNO, in which the red region indicates the area for the EDX spectra in **Figure S11**. EDX elemental maps of pLNO, PC-LNO, and PEC-LNO highlight (b) Ni intensity from the LNO particles and (c) C intensity from the PC and PEC polymer coating layers.

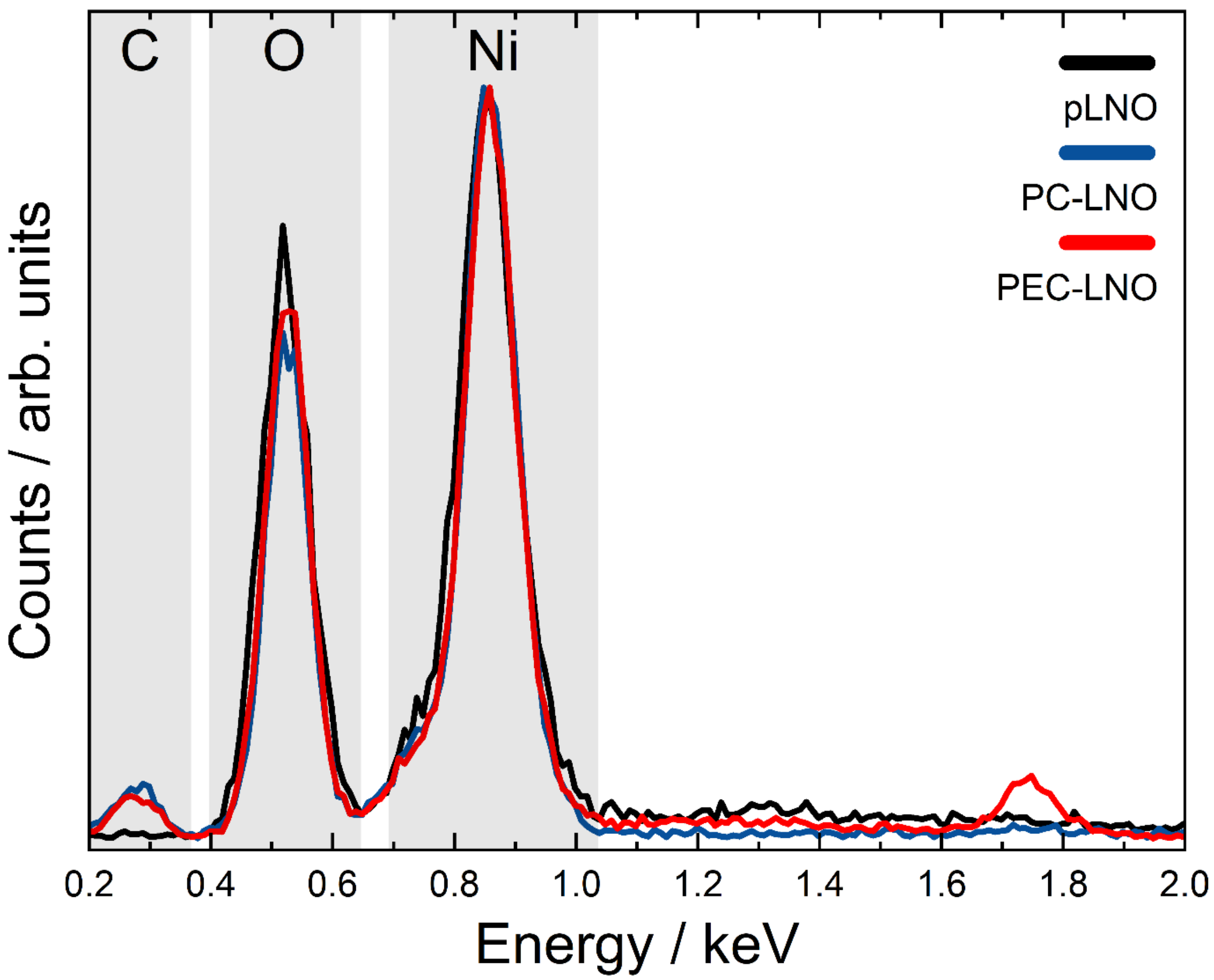

Figure S11. EDX spectra of pLNO, PC-LNO, and PEC-LNO are acquired from particle edges (red regions in **Figure S10a**). Ni represents LNO particles, while C indicates the PC and PEC polymer coating layers. The peak at 1.74 keV corresponds to Si, likely arising from impurities.

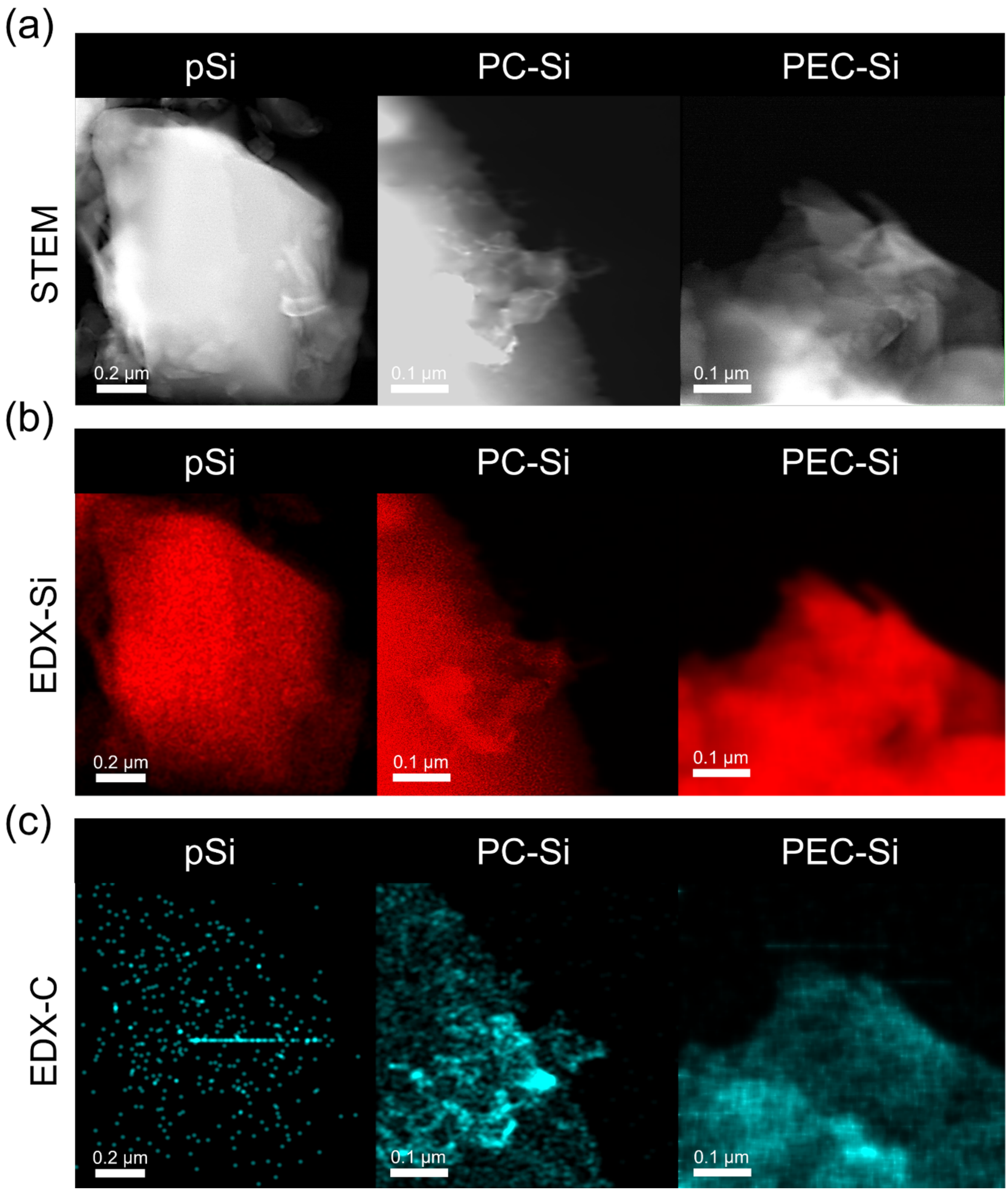

Figure S12. (a) STEM images of pSi, PC-Si, and PEC-Si, with the corresponding EDX spectra shown in **Figure S13**. EDX elemental maps of pSi, PC-Si, and PEC-Si highlight (b) Si intensity from the silicon particles and (c) C intensity from the PC and PEC polymer coating layers.

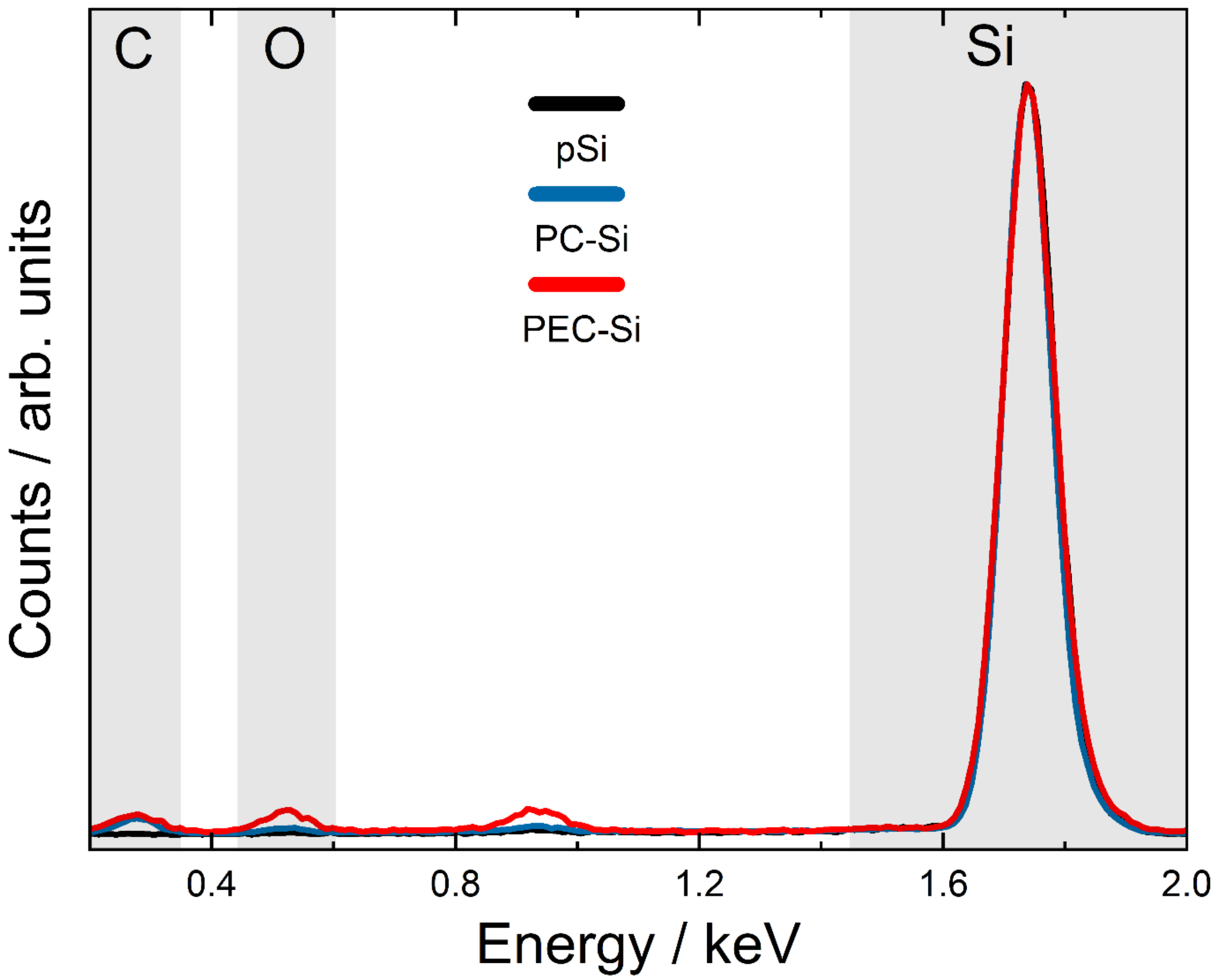

Figure S13. EDX spectra of pSi, PC-Si, and PEC-Si are acquired from particles shown in **Figure S12**. Si represents silicon particles, while C indicates the PC and PEC polymer coating layers. The peak at 0.9 keV corresponds to the Cu signal from the lacey carbon coated copper mesh grid.

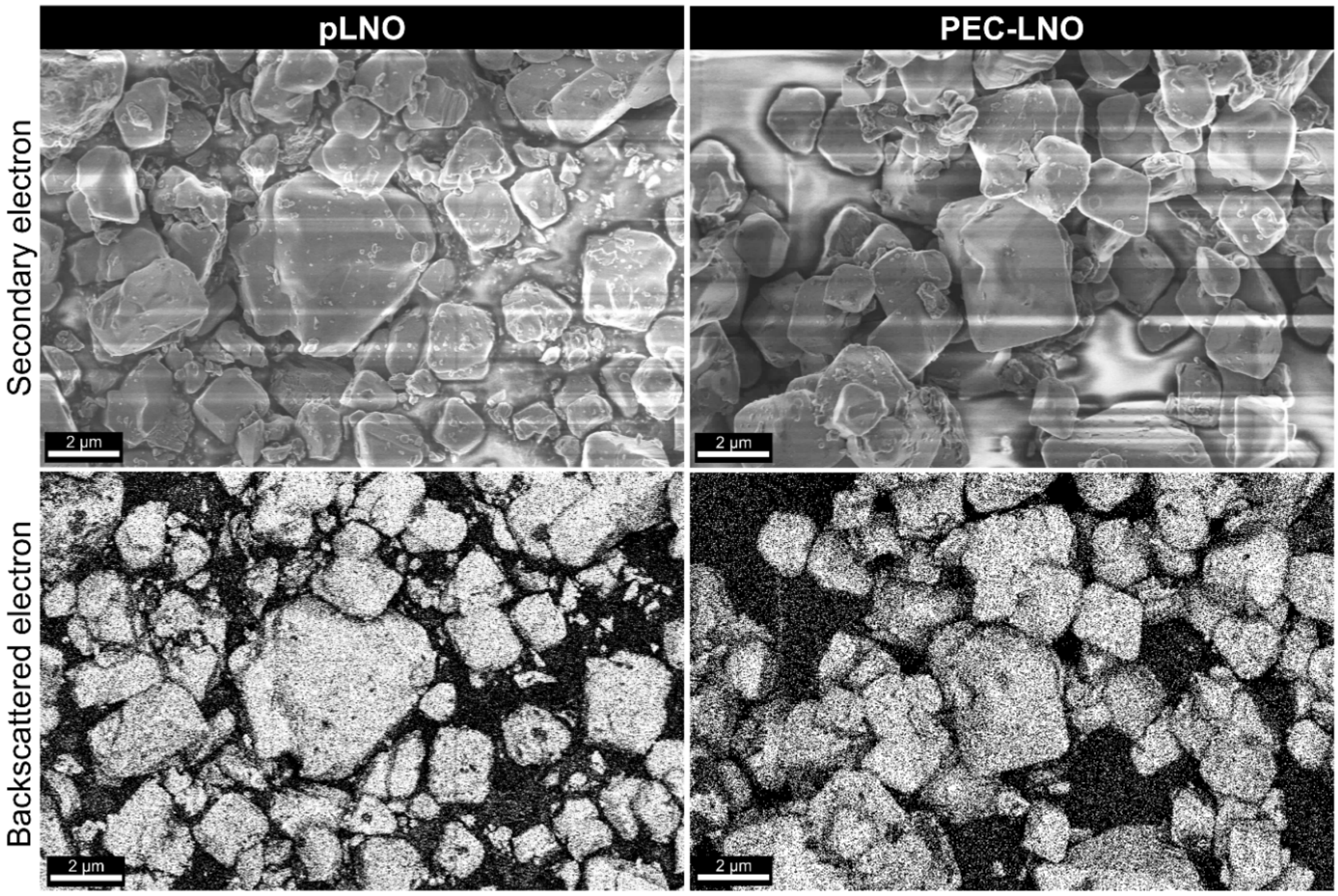

Figure S14. SEM analysis of pLNO (left) and PEC-LNO (right) is presented, with secondary electron images at the top and energy-selective backscattered electron images at the bottom.

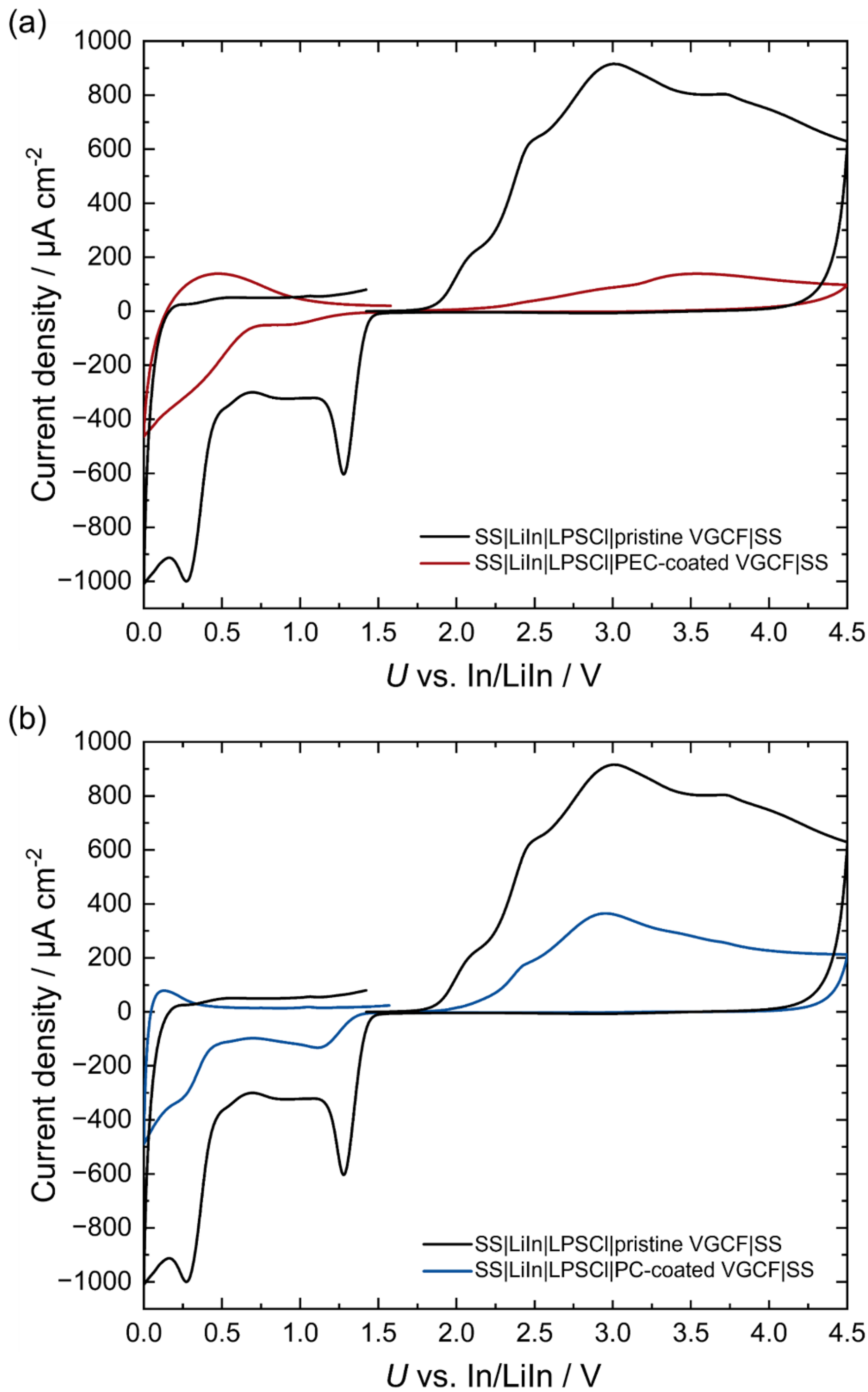

Figure S15. The electrochemical stability window is measured by CV for the range from 0 V to 4.5 V vs. In/LiIn with a scan rate of 1 mV s^{-1}, initiating from the OCP. VGCF is used as the working electrode, LPSCl is used as the separator, and LiIn alloy is used as the counter electrode. (a) PEC-coated VGCF and (b) PC-coated VGCF composite are compared with pristine VGCF.

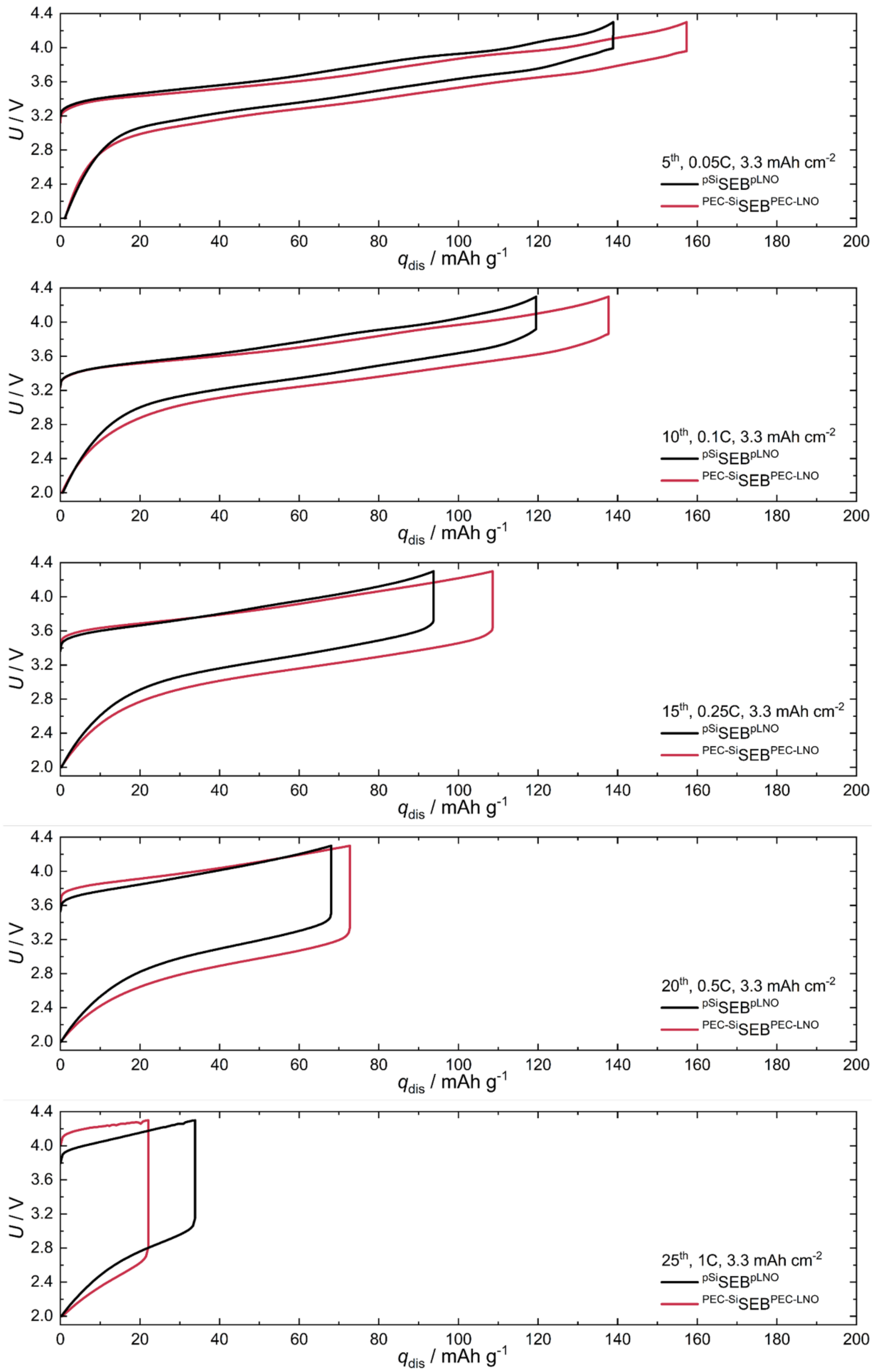

Figure S16. Galvanic charge and discharge plots of $^{pSi}SEB^{pLNO}$ and $^{PEC\text{-}Si}SEB^{PEC\text{-}LNO}$ (area capacity: 3.3 mAh·cm^{-2}, LNO: 13.2 mg, Si: 1 mg, N/P ratio: 1.3) for the 5th at 0.05C, 10th at 0.1C, 15th at 0.25C, 20th at 0.5C, and 25th cycle at 1C is presented.

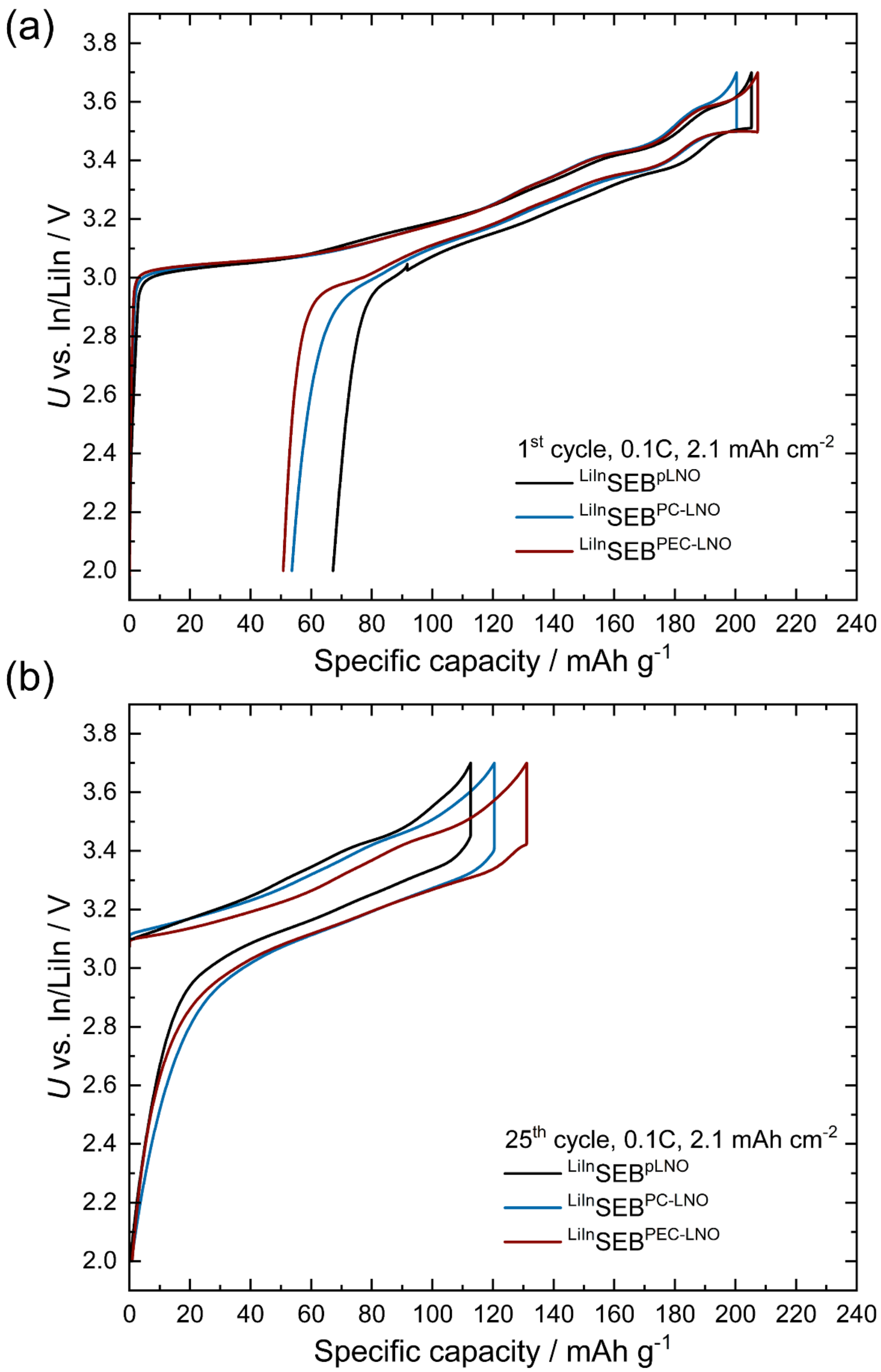

Figure S17. The galvanic plots at 0.1C of $^{LiIn}SEB^{pLNO}$, $^{LiIn}SEB^{PC\text{-}LNO}$, and $^{LiIn}SEB^{PEC\text{-}LNO}$ (area capacity: 2.1 mAh·cm^{-2}, LNO loading: 10.6 mg·cm^{-2}) for (a) the 1st cycle and (b) the 25th cycle are presented.

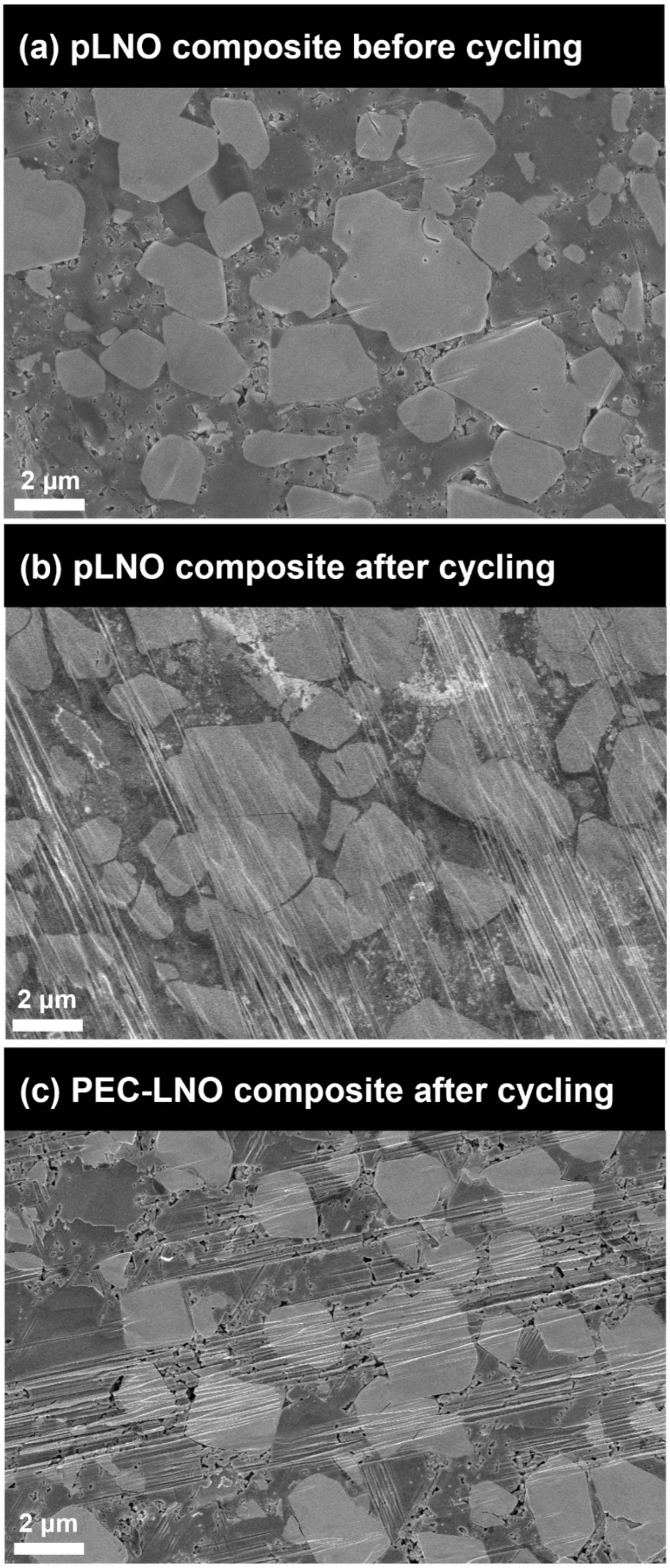

Figure S18. SEM images of cross-sectioned composite cathodes prepared by ion beam milling are shown, including (a) pLNO cathode composite before cycling, (b) pLNO cathode composite after 100 cycles at a 0.1C rate, and (c) PEC-LNO cathode composite after 100 cycles at a 0.1C rate.

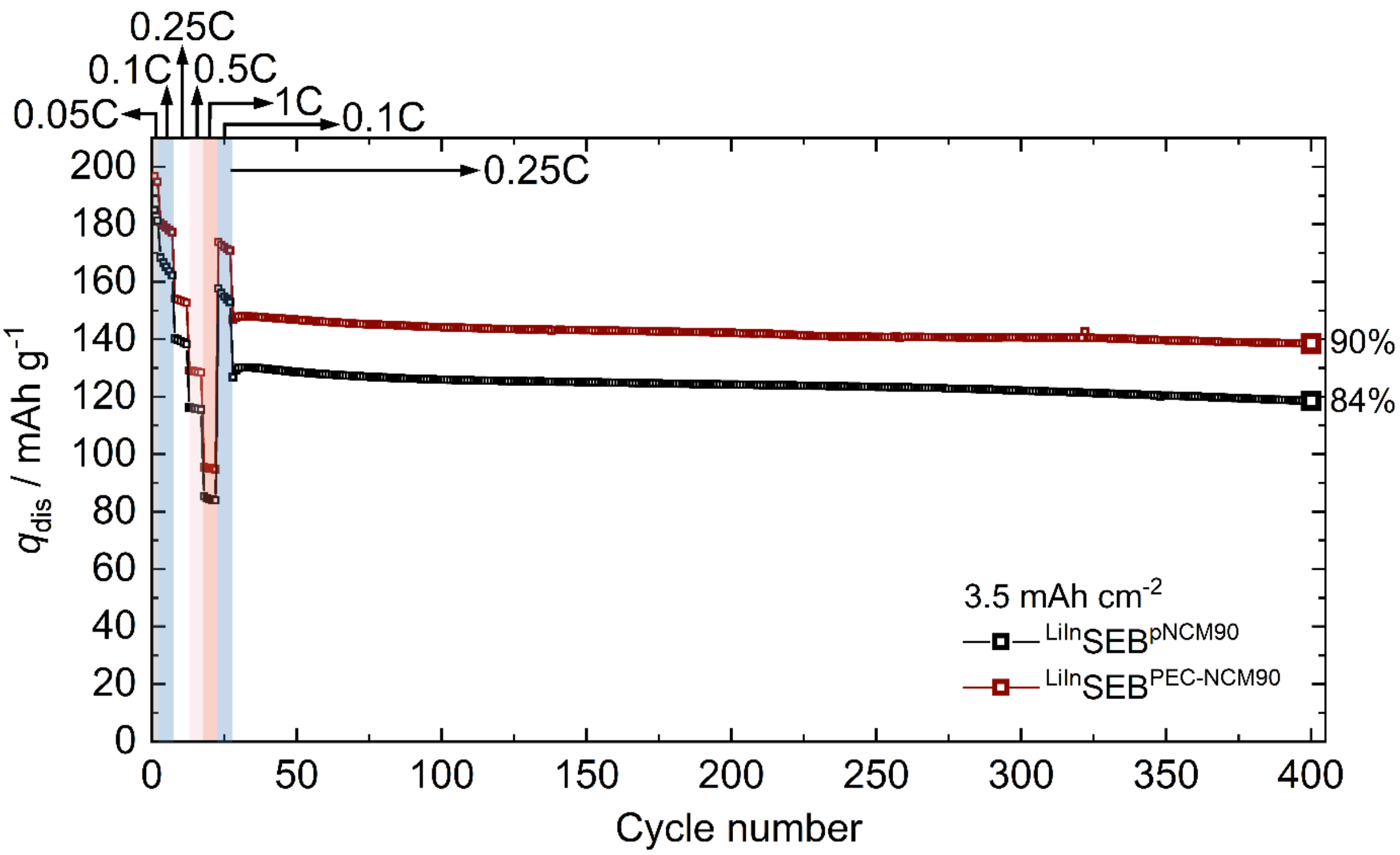

Figure S19. The electrochemical performance of $^{LiIn}SEB^{NCM90}$ with NCM90 as the CAM, LPSCl as the catholyte, $Li_{5.5}PS_{4.5}Cl_{0.8}Br_{0.7}$ (LPSClBr, 9.1 mS·cm^{-1})[1] as the separator, and LiIn as the anode is presented. 60 MPa of external pressure is applied during cycling. The PEC coated NCM90 and pNCM90 cells are denoted as $^{LiIn}SEB^{pNCM90}$ and $^{LiIn}SEB^{PEC\text{-}NCM90}$, respectively, with an area-specific capacity of 3.5 mAh·cm^{-2} and an CAM loading of 17.7 mg·cm^{-2}. The applied current is transferred to C-rates based on 200 mAh·g^{-1} of CAM. The q_{dis} is plotted over cycling sequence: 0.05C for the first two cycles, followed by 0.1C, 0.25C, 0.5C, and 1C for five cycles each, then returning to 0.1C for five cycles, and subsequently operated at 0.25C for an additional 373 cycles. Capacity retention is determined by comparing the 8th and 400th cycles.

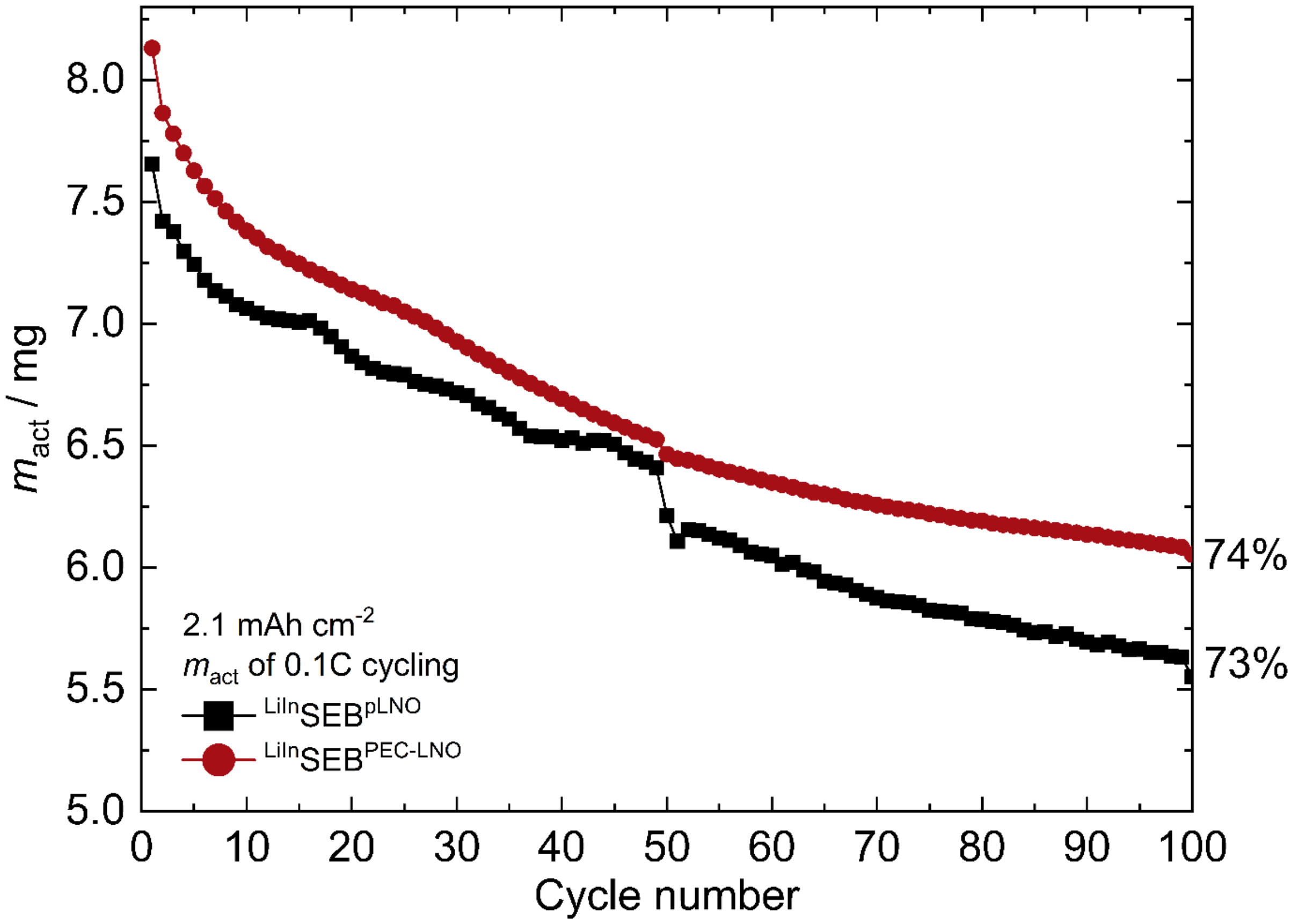

Figure S20. The m_{act} values for $^{LiIn}SEB^{pLNO}$ and $^{LiIn}SEB^{PEC\text{-}LNO}$ (area capacity: 2.1 mAh·cm^{-2}, LNO loading: 10.6 mg·cm^{-2}) after 100 cycles at 0.1C and 25 °C are calculated from their cycling performance.

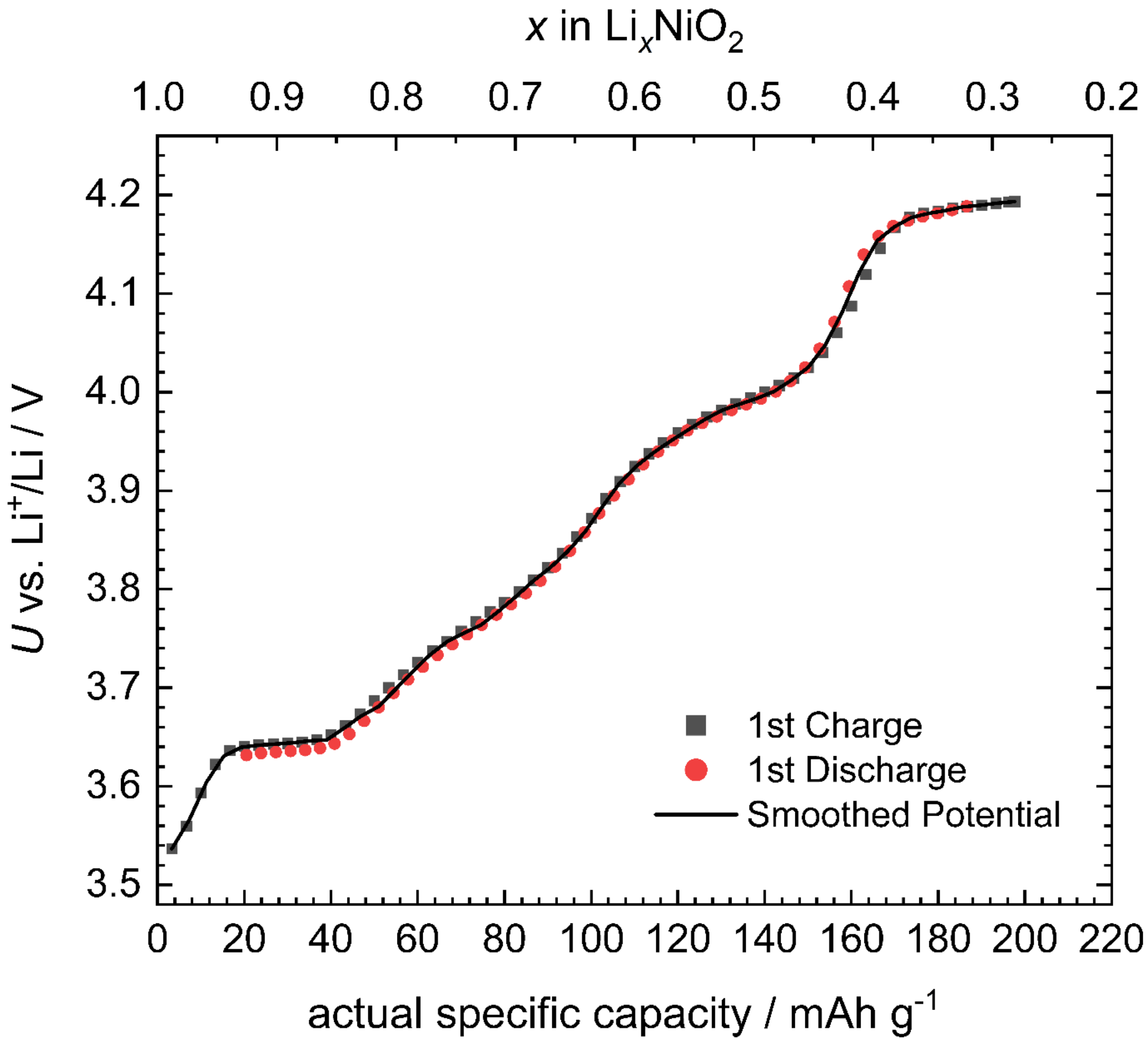

Figure S21. Relationship between OCP and the SOC, represented by the lithium content *x* in Li_xNiO_2. This is derived from the galvanostatic intermittent titration technique (GITT) of a $^{Li}LEB^{LNO}$ using 1 M $LiPF_6$ in EC: DEC (1:1 vol.%) LE, a lithium metal anode and LNO cathode sheet at a 0.1C rate. Each 10-minute charge or discharge interval is followed by a two-hour relaxation period to measure the OCP. We calculate lithium content *x* in Li_xNiO_2 by using the known capacity for each GITT pulse step.

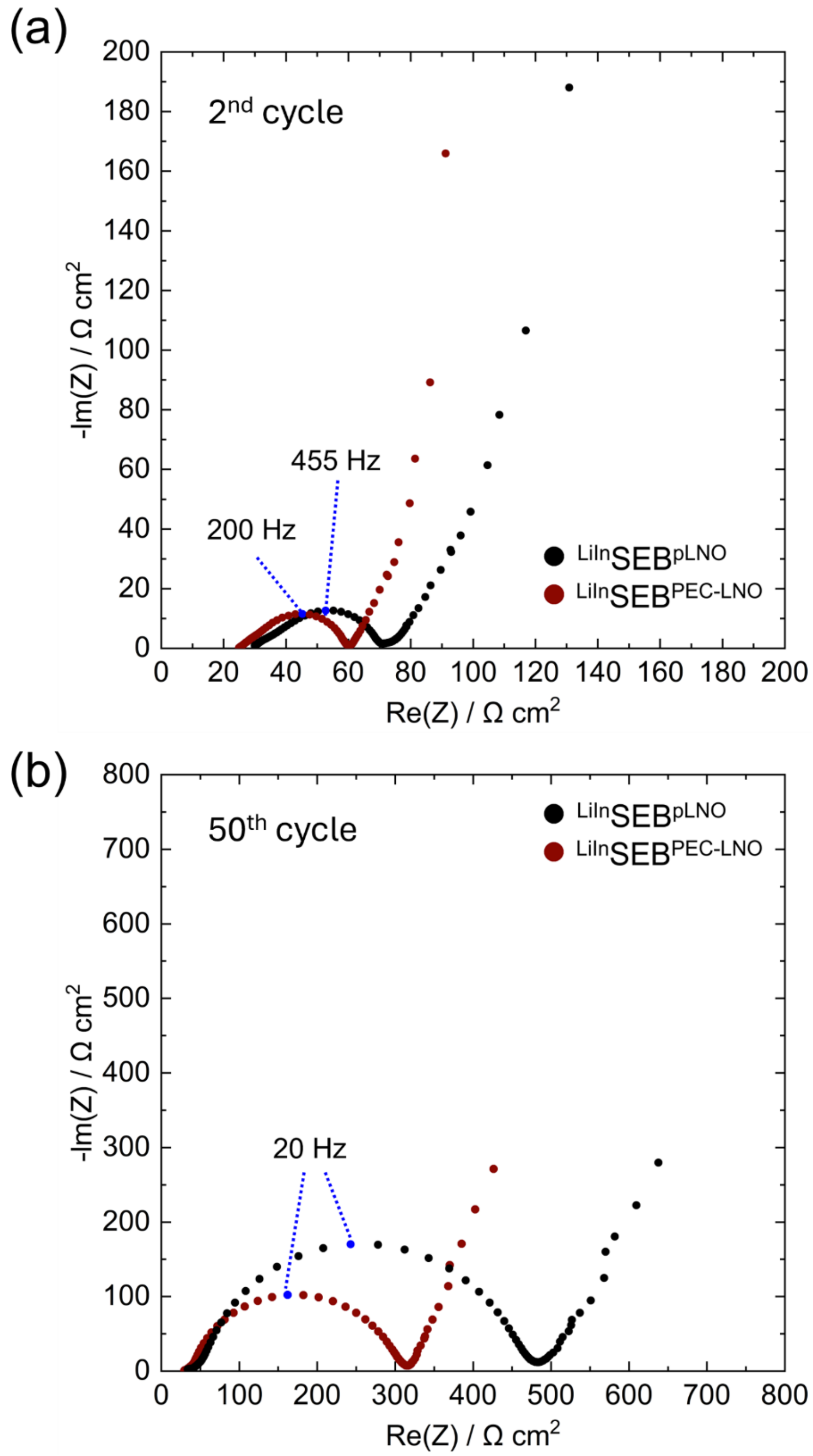

Figure S22. Nyquist plots during 0.1C cycling obtained at (a) 2nd and (b) 50th cycles of ^{LiIn}SEB^{LNO} cells (area capacity: 2.1 mAh·cm^{-2}, LNO loading: 10.6 mg·cm^{-2}), including ^{LiIn}SEB^{pLNO} and ^{LiIn}SEB$^{PEC\text{-}LNO}$, are presented. The apex frequency for both cells is highlighted in blue.

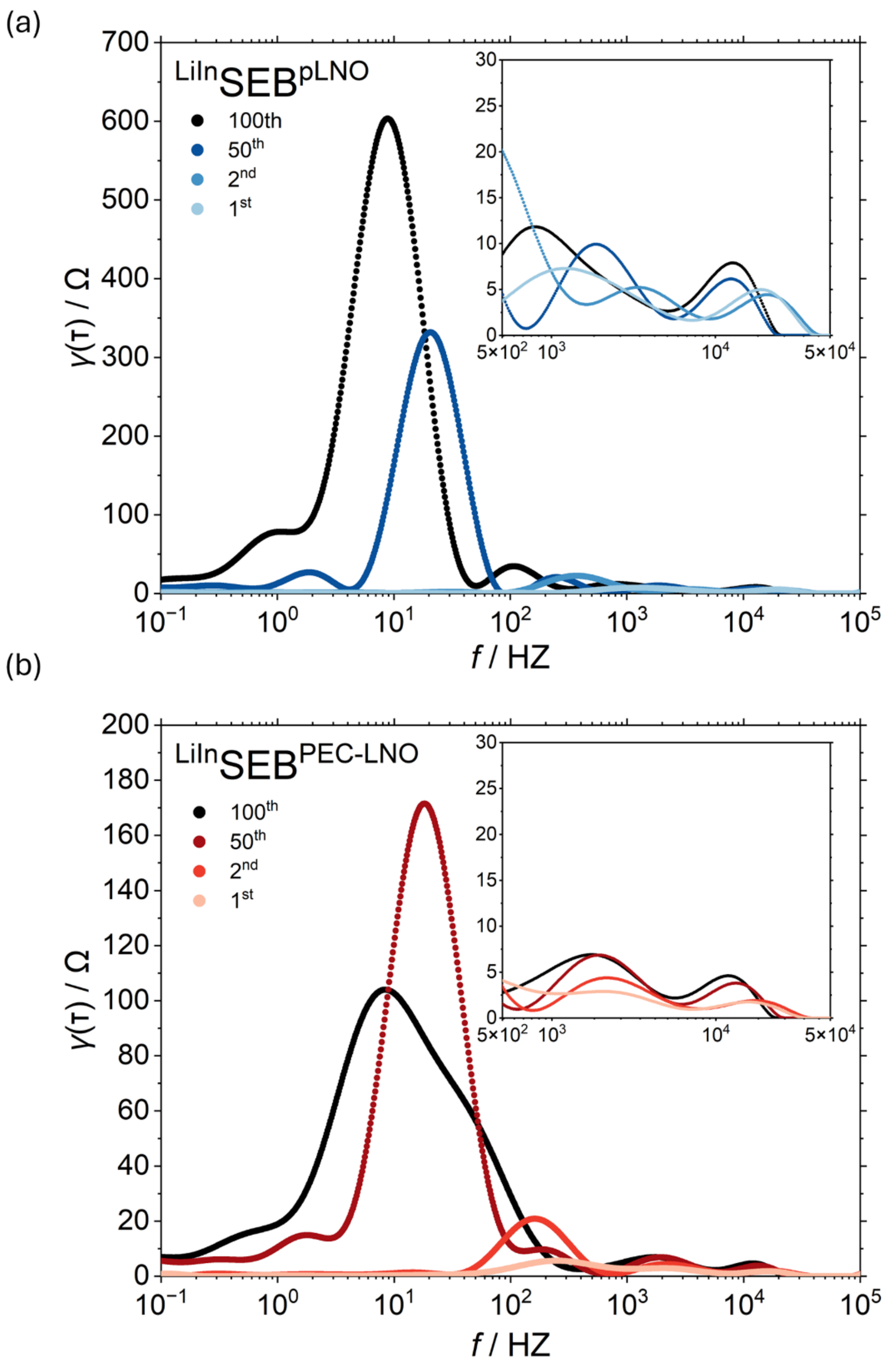

Figure S23. Distribution of relaxation times (DRT) analysis of the (a) $^{LiIn}SEB^{pLNO}$ (b) $^{LiIn}SEB^{PEC\text{-}LNO}$ impedance data shown in **Figure 5C** and **Figure S22**, plotted as $\gamma(\tau)$ / Ω versus frequency (f / Hz). Here, $\gamma(\tau)$ denotes the DRT, representing the contribution to the total resistance as a function of the time constant τ. The inset highlights the frequency range from 5×10^2 to 5×10^4 HZ.

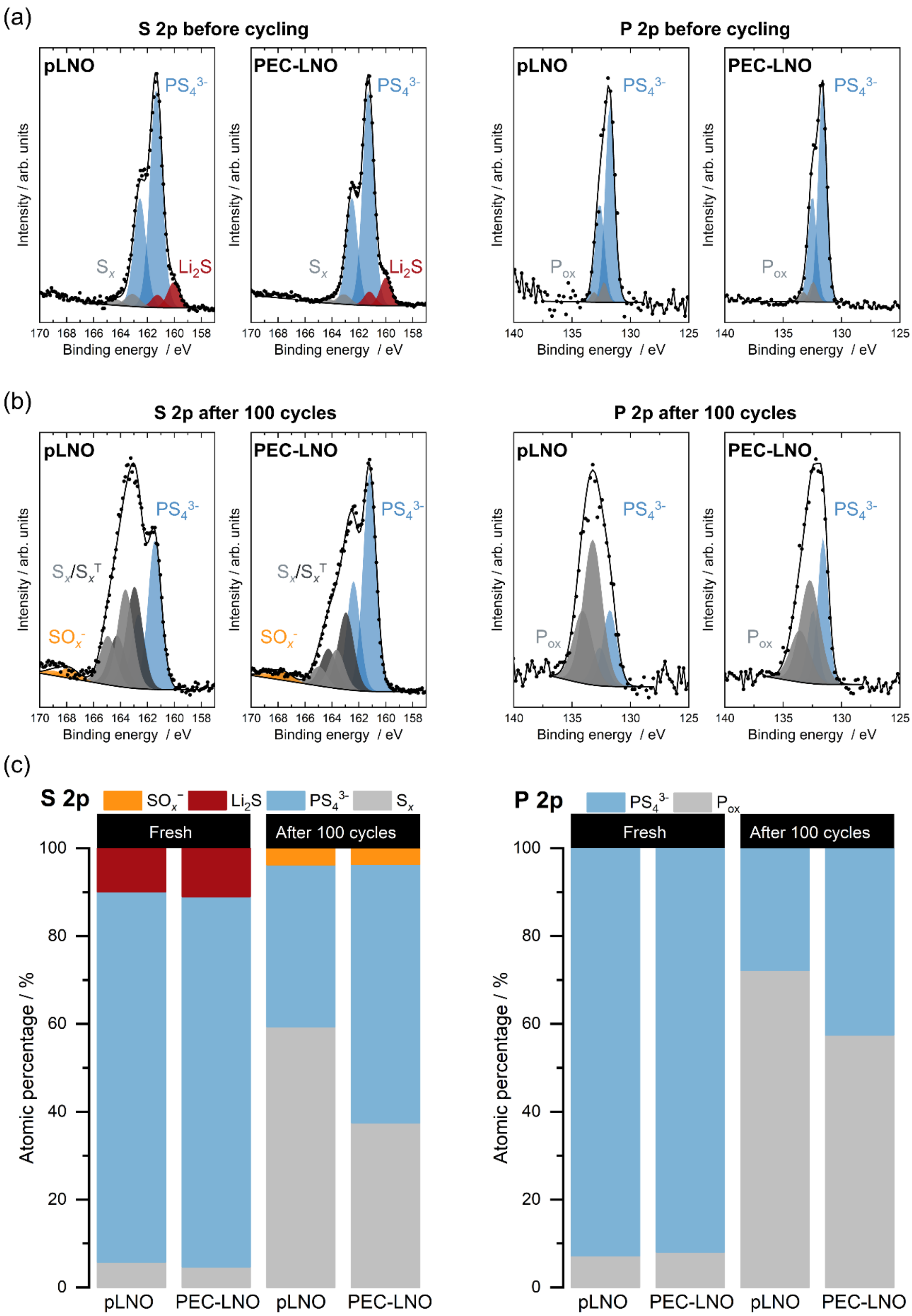

Figure S24. S 2p and P 2p XPS spectra of $^{LiIn}SEB^{pLNO}$ (pLNO) and $^{LiIn}SEB^{PEC\text{-}LNO}$ (PEC-LNO) (a) before and (b) after cycling, highlighting differences in interfacial side-reaction products. (c) Relative ratios of S- and P-containing side reaction products extracted from the quantitative fitting of XPS spectra.

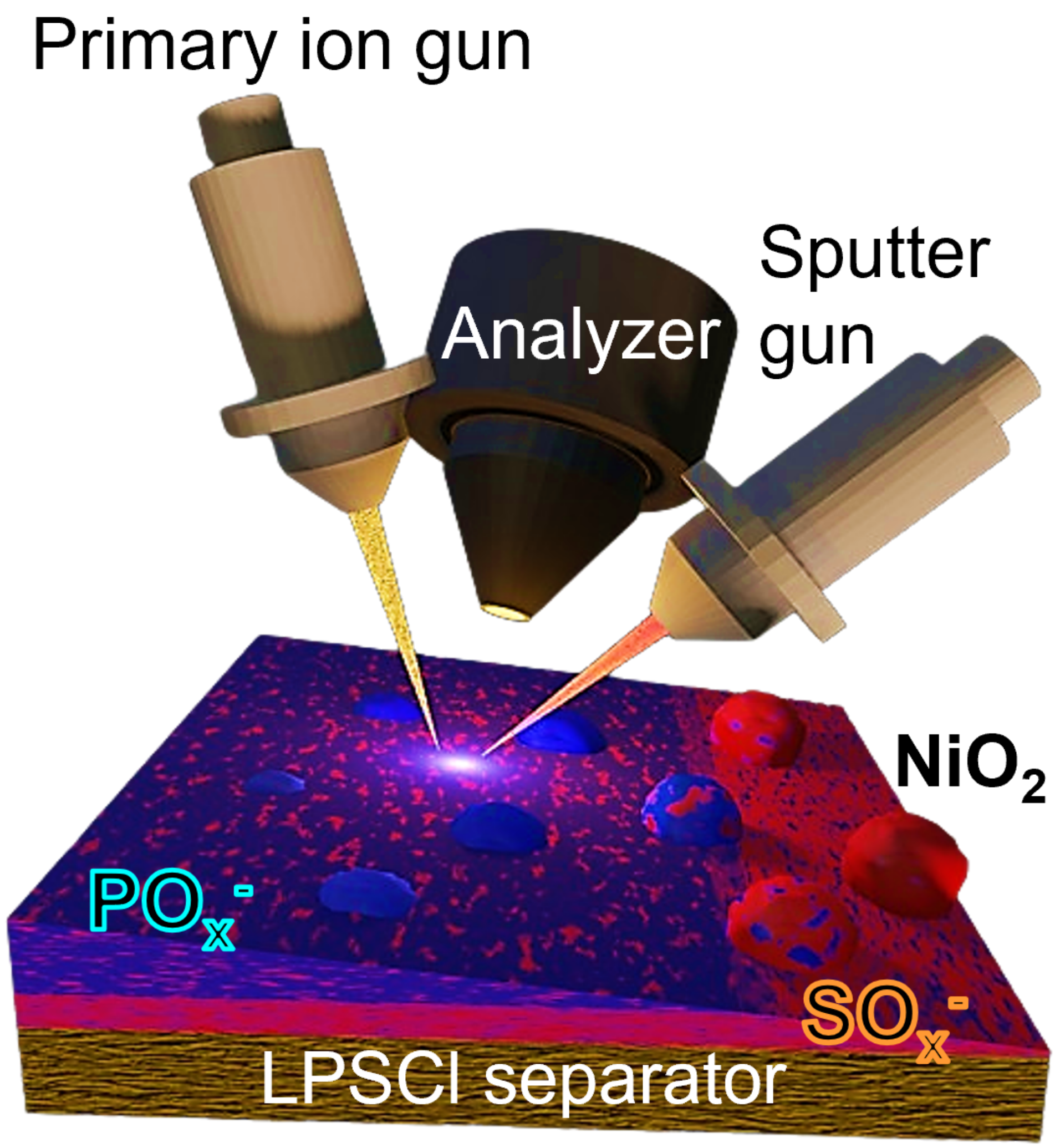

Figure S25. A schematic illustrates wedge-shaped crater formation by increasing sputter dose density from left to right. Subsequently, ToF-SIMS is used to detect the side reaction products. ToF-SIMS detects PO_x^- (PO^-, PO_2^-, and PO_3^-) and SO_x^- (SO^- and SO_2^-) decomposition products in the cathode composite of $^{LiIn}SEB^{pLNO}$ and $^{LiIn}SEB^{PEC\text{-}LNO}$ before and after cycling.

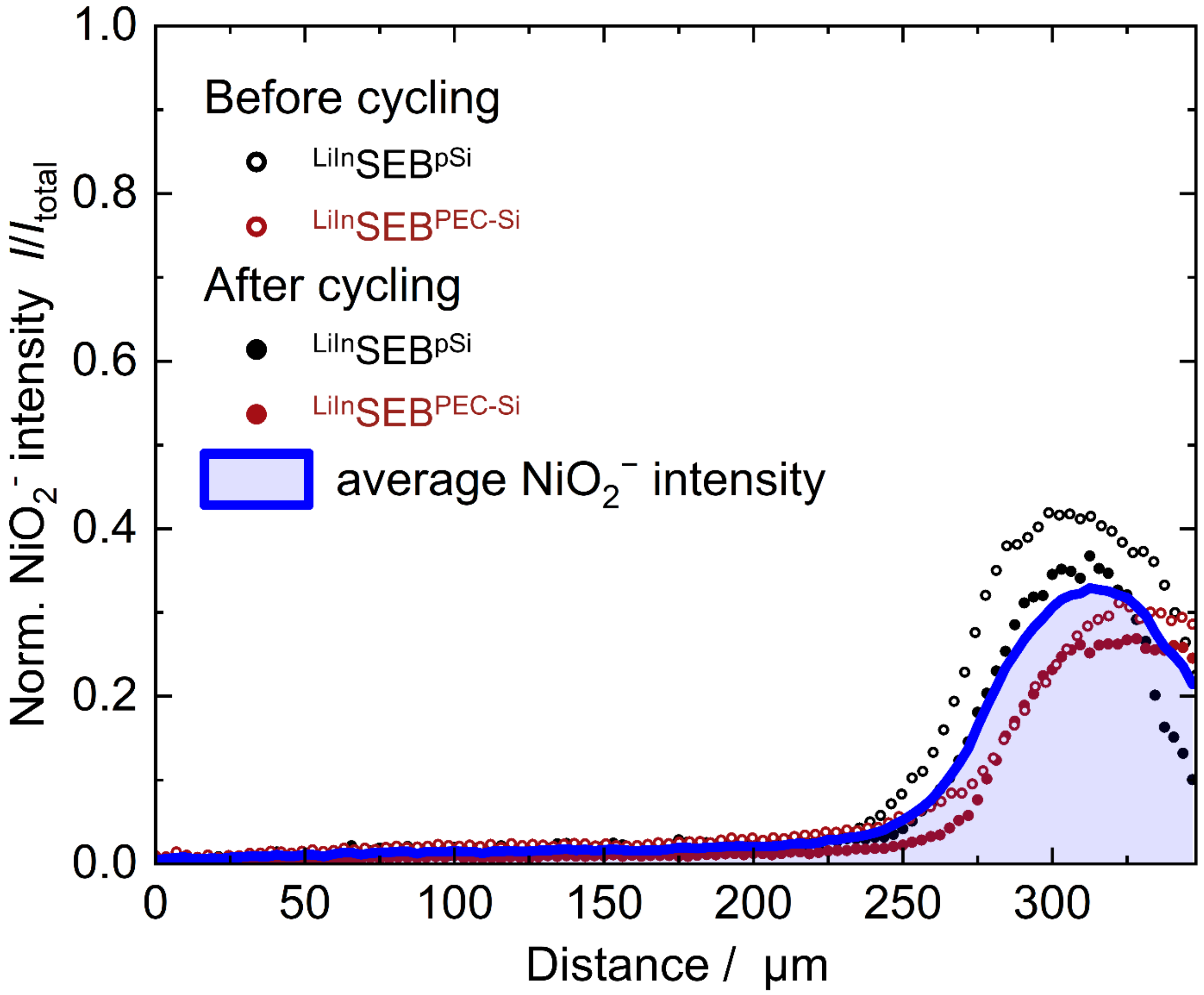

Figure S26. The NiO_2^- intensities (normalized by total intensity), in $^{LiIn}SEB^{pLNO}$ and $^{LiIn}SEB^{PEC\text{-}LNO}$ (before cycling and after 100 cycles), are measured using ToF-SIMS surface spectra after creating wedge-shaped sputter craters.

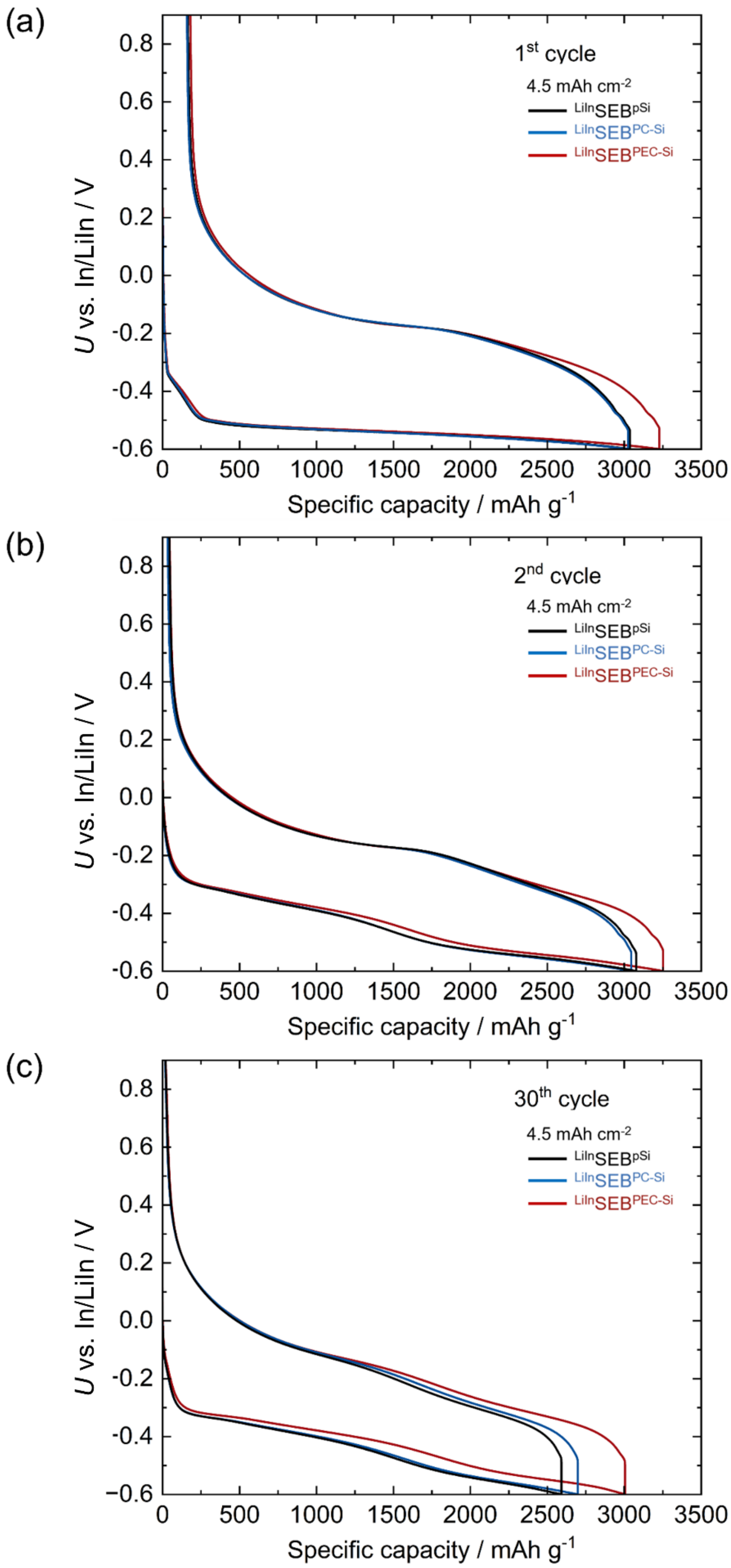

Figure S27. The galvanic plots of $^{LiIn}SEB^{Si}$ (area capacity: 4.5 mAh·cm^{-2}, Si loading: 1.3 mg·cm^{-2}), including $^{LiIn}SEB^{pSi}$, $^{LiIn}SEB^{PC\text{-}Si}$, and $^{LiIn}SEB^{PEC\text{-}Si}$, for (a) the 1st cycle at 0.05C, (b) the 2nd cycle at 0.05C, and (c) the 30th cycle at 0.1C are presented.

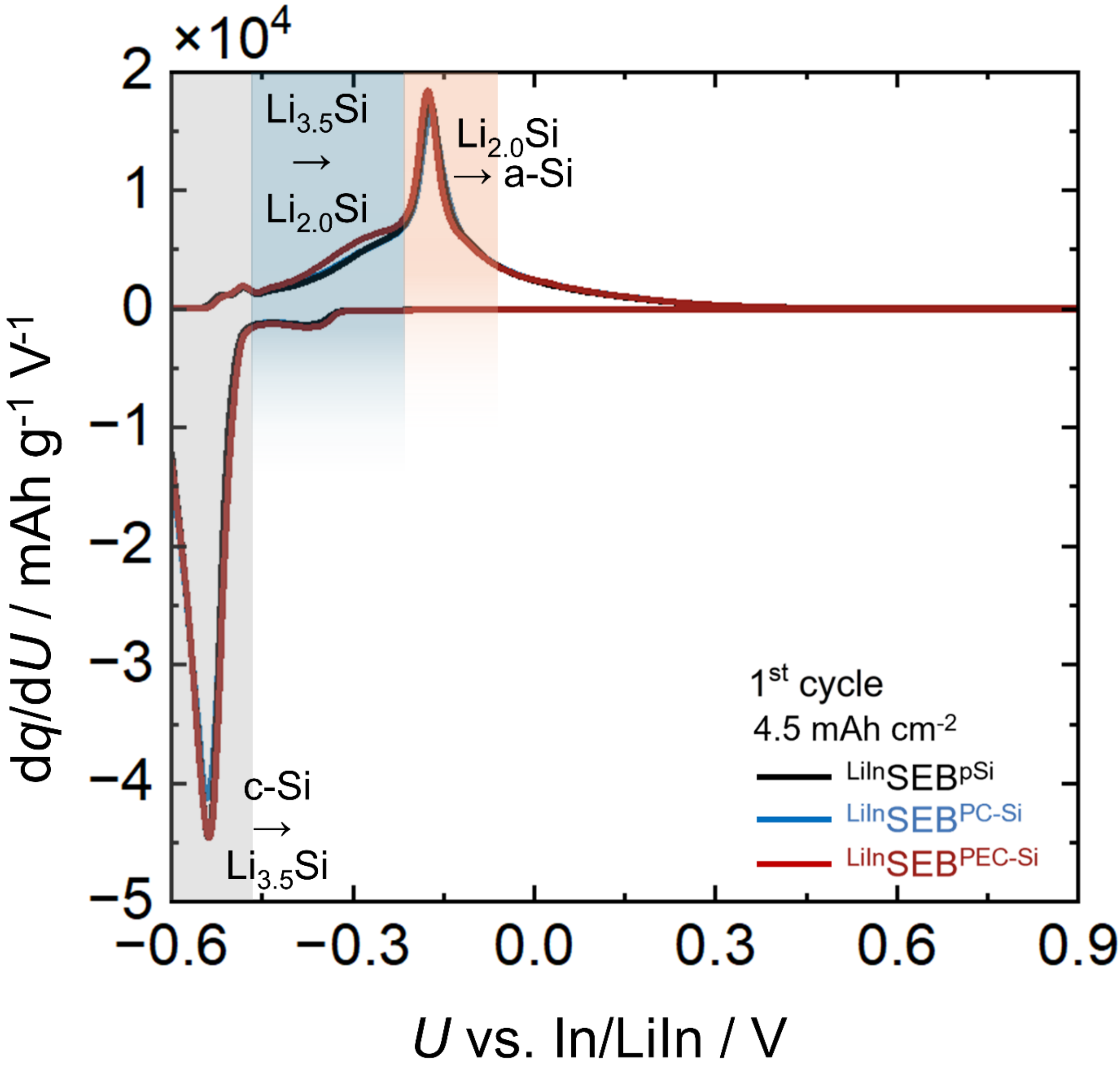

Figure S28. The d*q*/d*U* plot for the first cycle at 0.1C of $^{LiIn}SEB^{Si}$ (area capacity: 4.5 mAh·cm^{-2}, Si loading: 1.3 mg·cm^{-2}), including $^{LiIn}SEB^{pSi}$, $^{LiIn}SEB^{PC\text{-}Si}$, and $^{LiIn}SEB^{PEC\text{-}Si}$, highlighting the silicon phase transitions (c-Si→$Li_{3.5}Si$↔$Li_{2.0}Si$↔ a-Si).

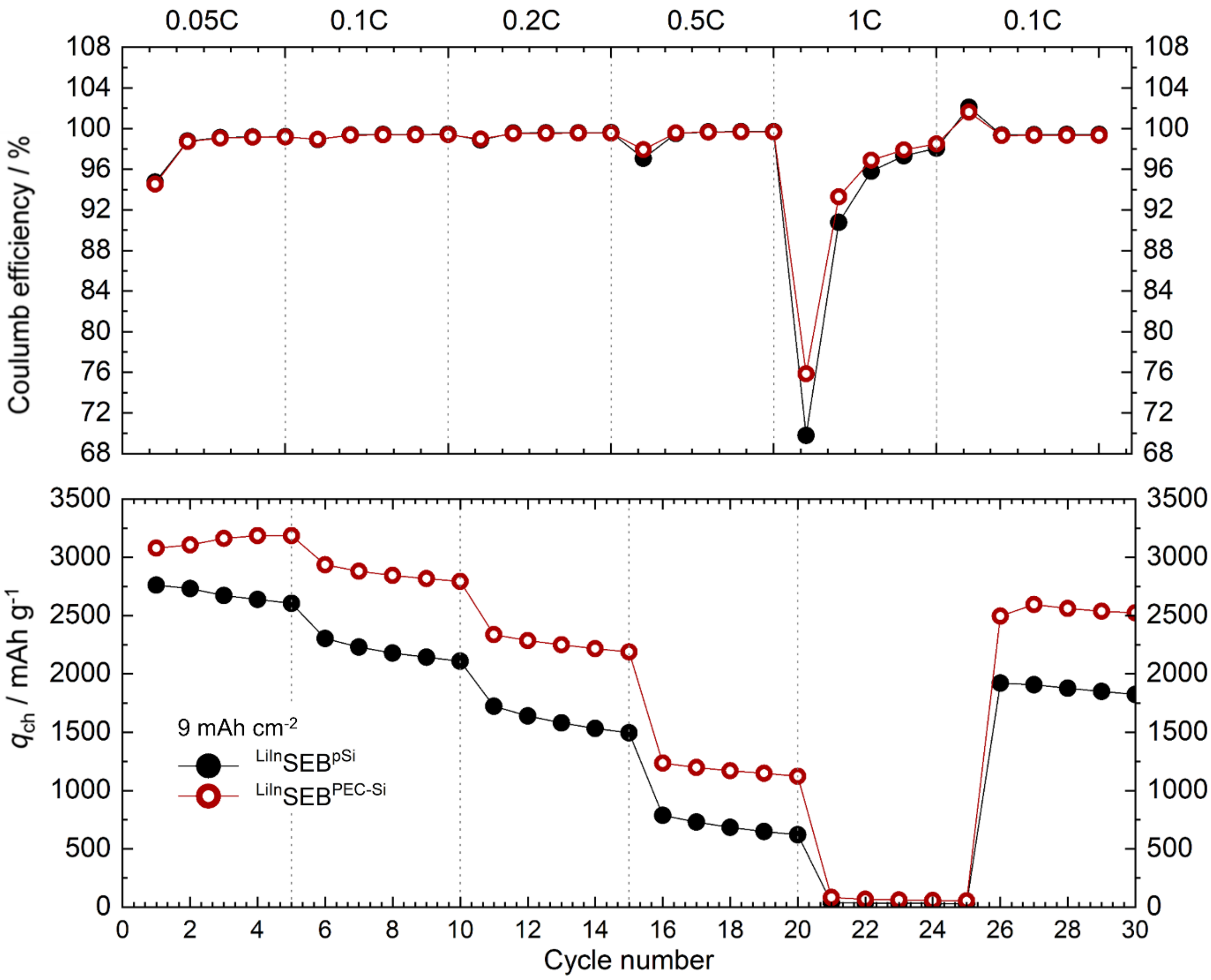

Figure S29. Rate capability of $^{LiIn}SEB^{Si}$ (area capacity: 9 mAh·cm^{-2}, Si loading: 2.6 mg·cm^{-2}), including $^{LiIn}SEB^{pSi}$ and $^{LiIn}SEB^{PEC\text{-}Si}$, is analyzed using pellet-type cells. Coulomb efficiency (top) and q_{ch} (bottom) are presented across different C-rates (0.05C, 0.1C, 0.2C, 0.5C, 1C, and back to 0.1C).

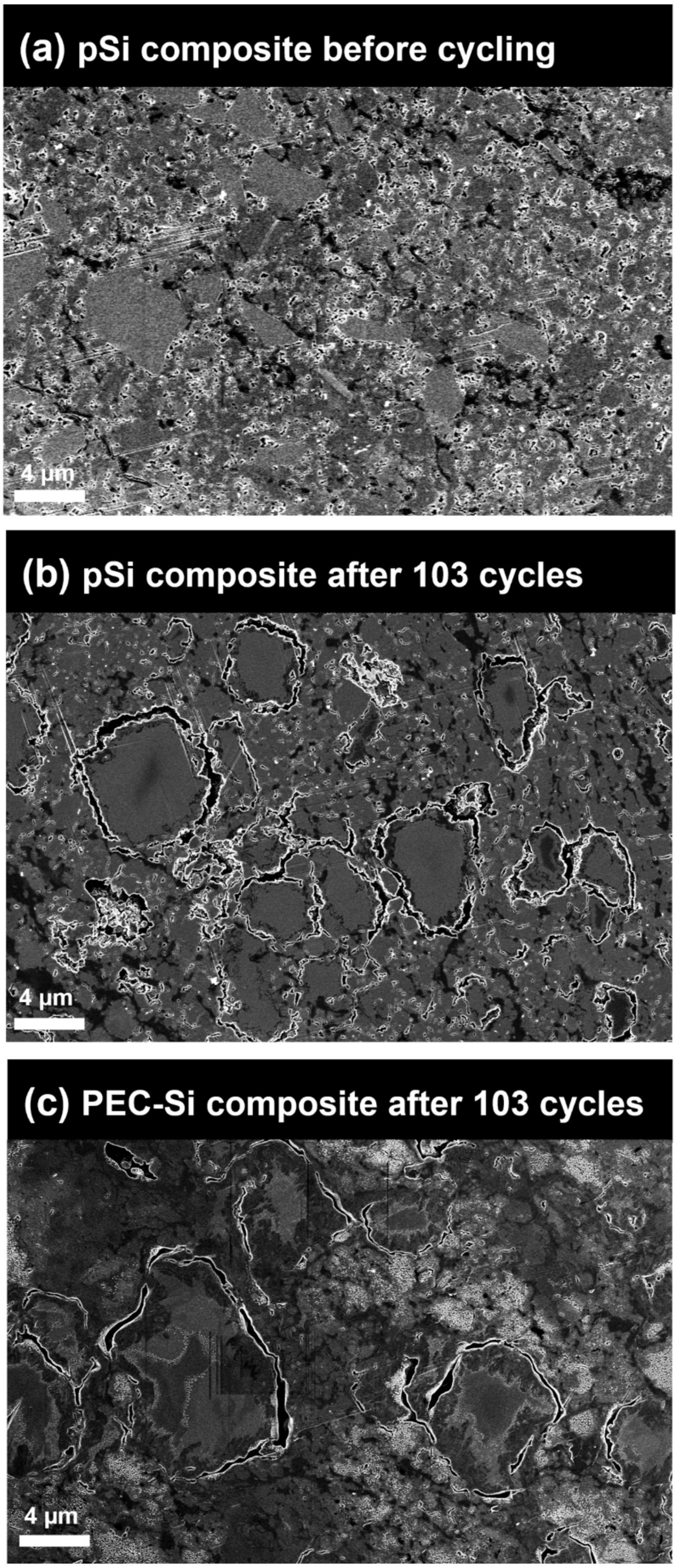

Figure S30. SEM images of cross-sectioned composite cathodes prepared by ion beam milling. (a) pSi anode composite before cycling, (b) pSi anode composite after 103 cycles, and (c) PEC-Si anode composite after 103 cycles.

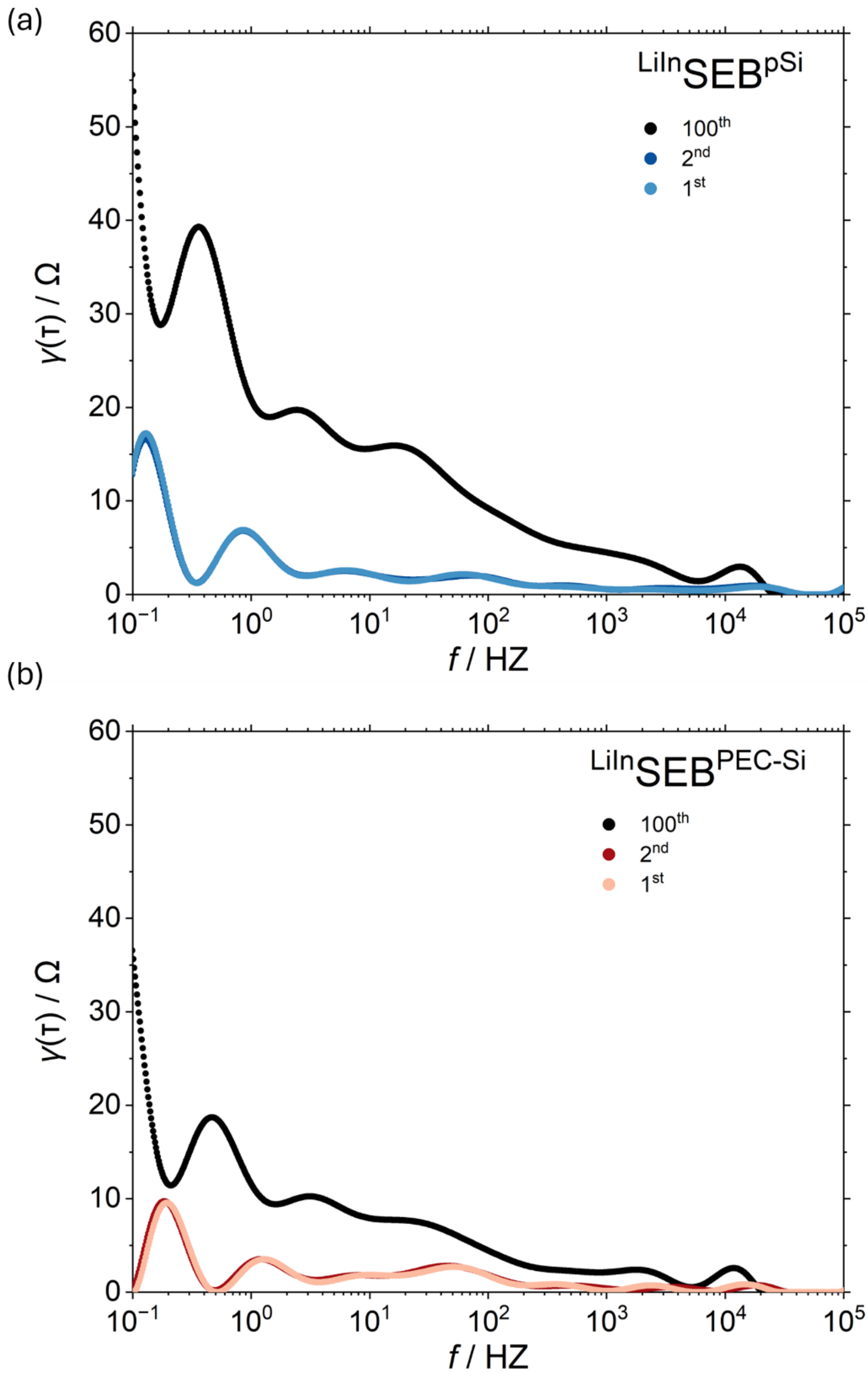

Figure S31. Distribution of relaxation times (DRT) analysis of the (a) $^{LiIn}SEB^{pSi}$ (b) $^{LiIn}SEB^{PEC\text{-}Si}$ impedance data shown in **Figure 8C**, plotted as $\gamma(\tau)$ / Ω versus frequency (f / Hz). Here, $\gamma(\tau)$ denotes the DRT, representing the contribution to the total resistance as a function of the time constant τ.

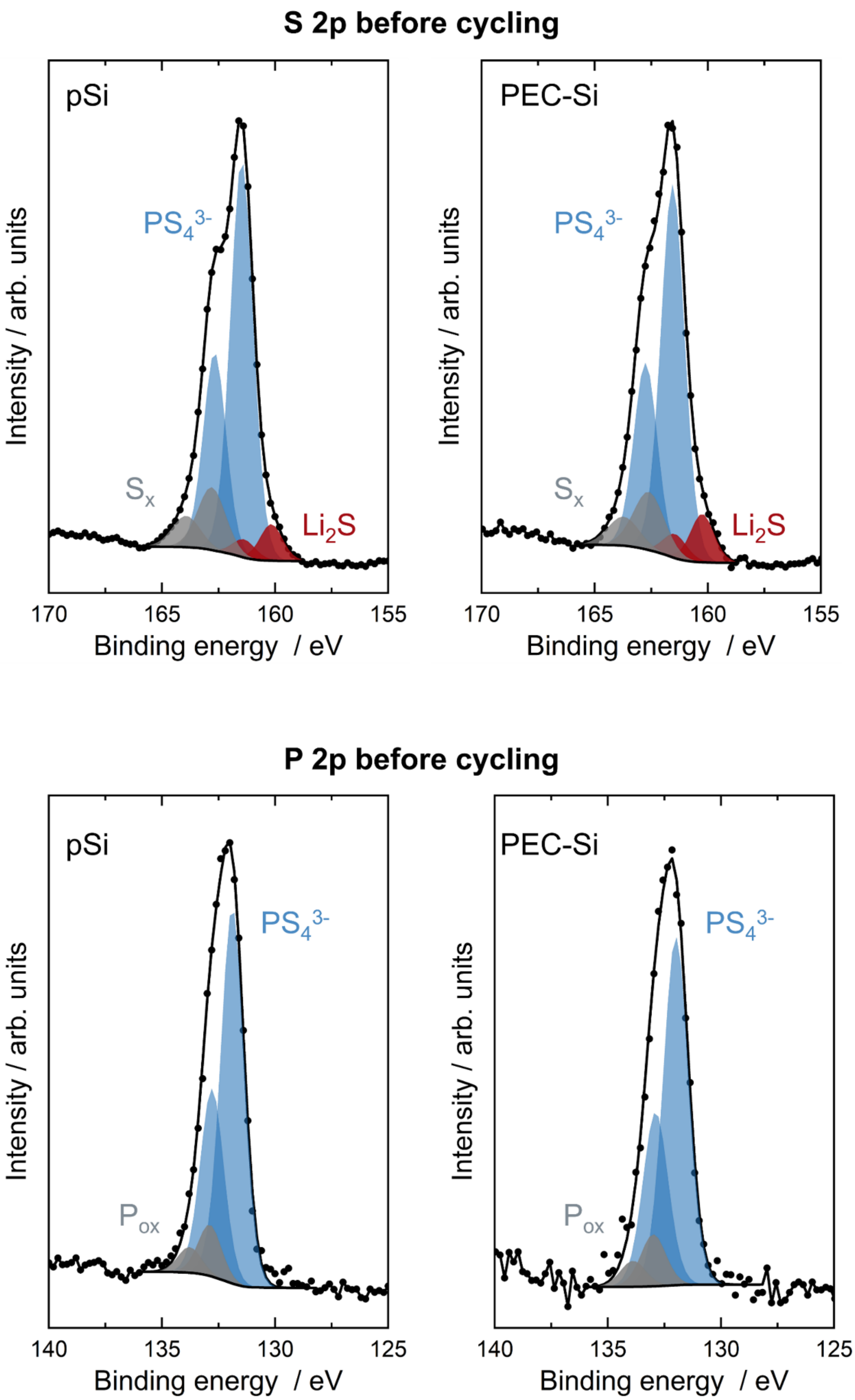

Figure S32. S 2p and P 2p XPS spectra of $^{LiIn}SEB^{pSi}$ (pSi) and $^{LiIn}SEB^{PEC\text{-}Si}$ (PEC-Si) before cycling, highlighting differences in interfacial side-reaction products.

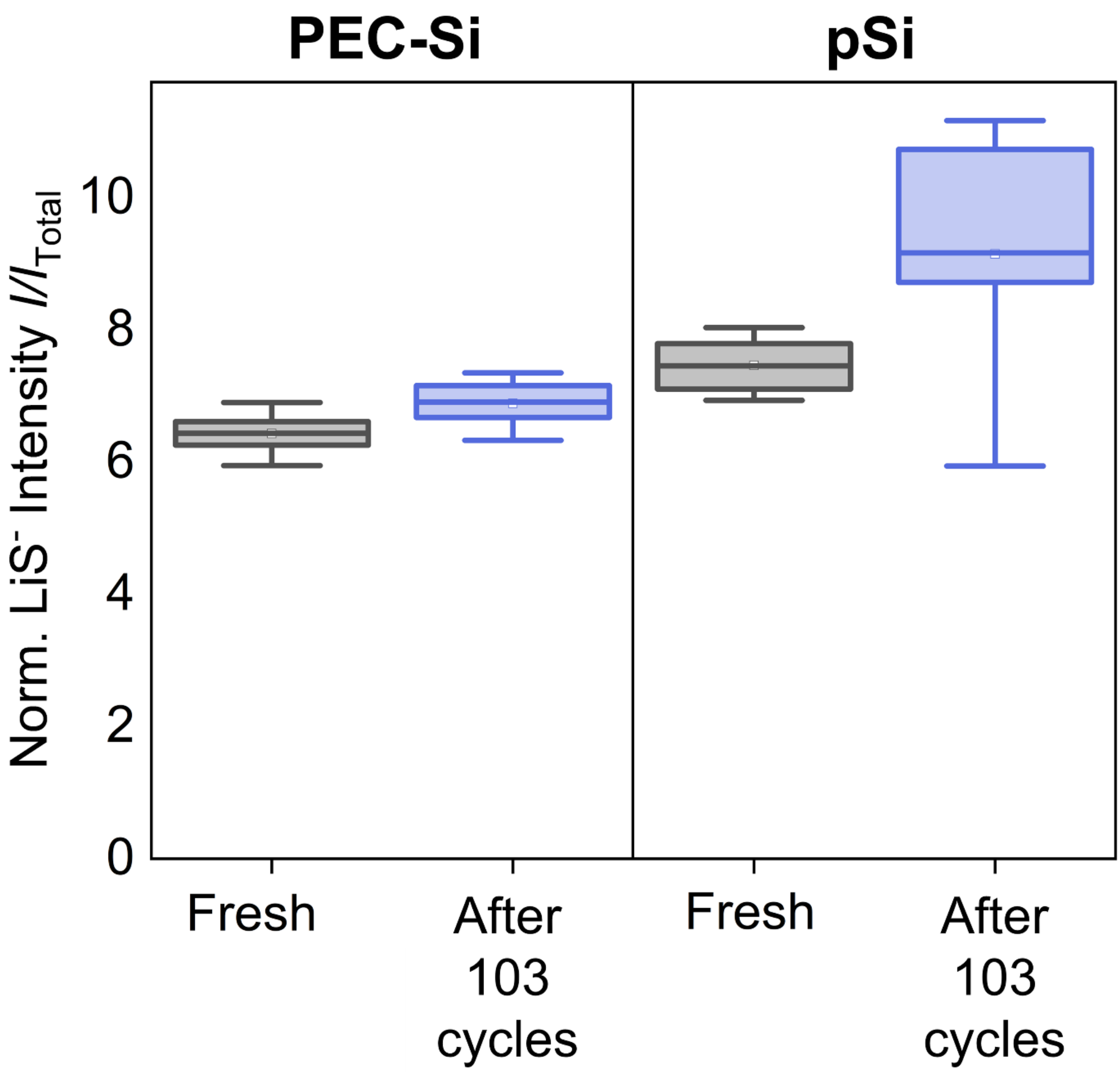

Figure S33. ToF-SIMS detects the LiS^- fragment which represenrts Li_2S decomposition products at the SEI in $^{LiIn}SEB^{pSi}$ and $^{LiIn}SEB^{PEC\text{-}Si}$ after 103 cycles, in comparison with the samples before cycling. The LiS^- fragment intensity is presented as boxplots with normalized intensities based on six data points.

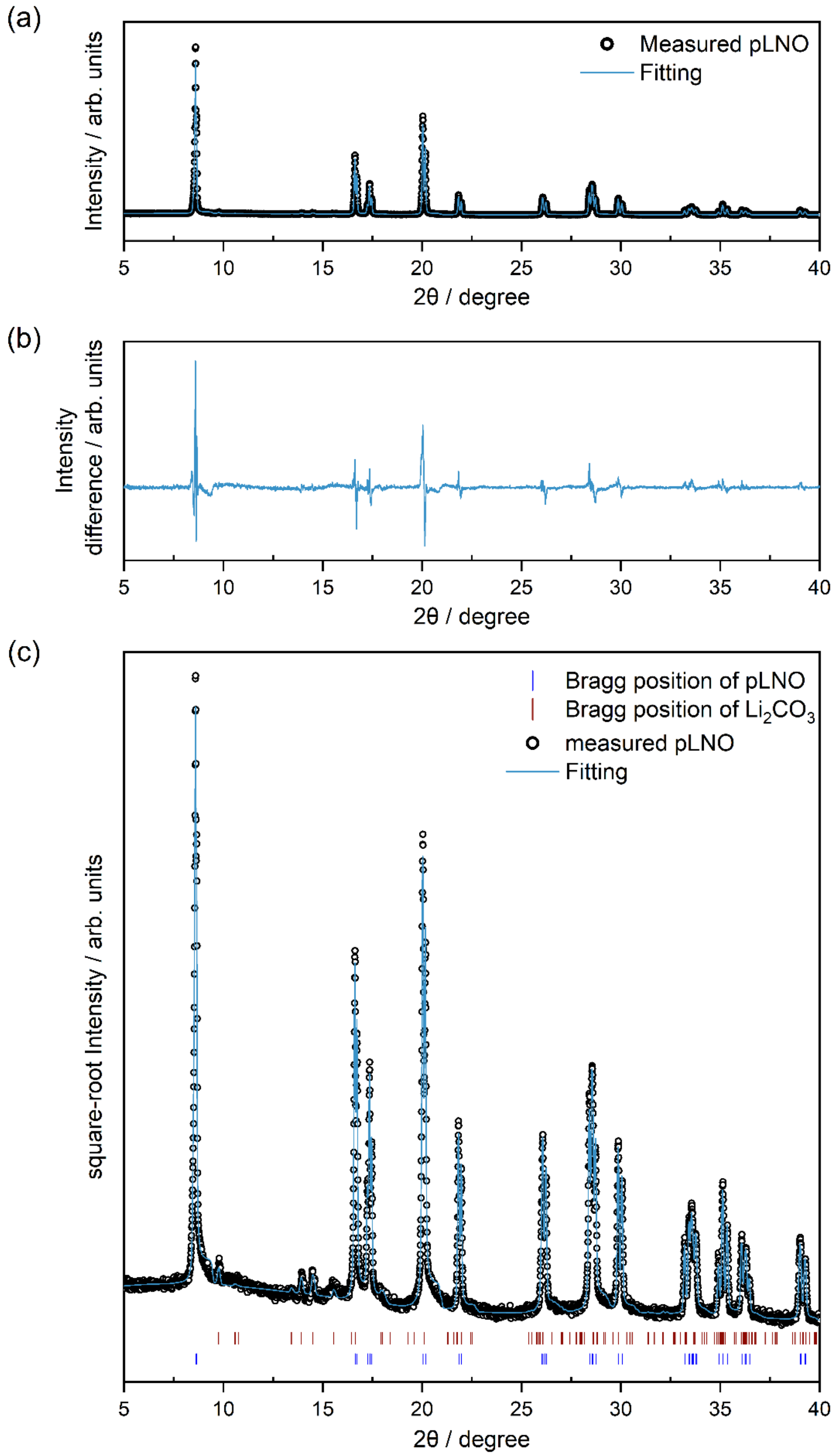

Figure S34. (a) XRD refinement pattern of pLNO with measured and fitted profiles. (b) Difference curve between the measured and fitted patterns. (c) Square-root intensity plot with Bragg positions,[2] highlighting both weak and strong peaks and confirming the pLNO crystalline structure and residual Li_2CO_3 precursor.

References

1. S. Li, J. Lin, M. Schaller, S. Indris, X. Zhang, T. Brezesinski, C.-W. Nan, S. Wang and F. Strauss, *Angewandte Chemie International Edition*, 2023, **62**, e202314155.
2. R. Stoyanova, E. Zhecheva, R. Alcántara, J. L. Tirado, G. Bromiley, F. Bromiley and T. Boffa Ballaran, *Solid State Ionics*, 2003, **161**, 197-204.
